# Potential Proteins Associated with Canine Epididymal Sperm Motility

**DOI:** 10.3390/cells15010085

**Published:** 2026-01-04

**Authors:** Marzena Mogielnicka-Brzozowska, Aleksandra Wiktoria Cichowska-Likszo, Pawel Likszo, Leyland Fraser, Weronika Popielarczyk, Julia Pieklik, Maja Kamińska, Gaia Cecilia Luvoni

**Affiliations:** 1 Department of Animal Biochemistry and Biotechnology, University of Warmia and Mazury in Olsztyn, Oczapowskiego 5, 10-719 Olsztyn, Poland; 2 Team of Reproductive Pathology and Translational Medicine, Institute of Animal Reproduction and Food Research, Polish Academy of Sciences in Olsztyn, Trylińskiego 18, 10-683 Olsztyn, Poland; 3 Department of Veterinary Medicine and Animal Sciences (DIVAS), University of Milan, 26900 Lodi, Italy

**Keywords:** epididymal sperm, proteins, sperm motility, canine

## Abstract

**Highlights:**

**What are the main findings?**

- Dog epididymal sperm with differing progressive motility exhibit unique protein profile signatures.
- Ce10 and NPC2 differed significantly between good and poor sperm motility groups and may serve as motility-related sperm proteins.

**What are the implications of the main findings?**

- A comprehensive analysis of the protein components of epididymal sperm in relation to their functional features may serve to select protein markers of epididymal semen quality.
- Ce10 and NPC2 emerge as promising candidates for molecular biomarkers associated with canine epididymal sperm motility.

**Abstract:**

The maturation and motility of epididymal sperm (ES) cells are largely driven by changes in protein expression. This study aimed to analyze the proteomic profile of canine (*Canis lupus familiaris*) ES across groups characterized by different progressive motility (PMOT) values to identify motility-related sperm proteins (MRSPs). ES were obtained from the epididymal semen of 19 dogs. The motility and movement parameters of ejaculated sperm (ES) were evaluated using computer-assisted semen analysis (CASA). Samples were classified into two groups: good sperm motility (GSM), defined as PMOT% ≥ 55%, and poor sperm motility (PSM), defined as PMOT < 55%. Principal component analysis (PCA) of the first two components could explain 88.1% of the total variance between the GSM and PSM groups. Protein profiling of ES was performed using NanoUPLC-Q-TOF/MS. Significant statistical differences were demonstrated between the GSM and PSM groups for the TMOT (*p* = 0.039) and PMOT (*p* < 0.001). For five common proteins, their abundance was estimated to be higher in the GSM group than in the PSM group: ACTB (*p* = 0.2732), CRISP2 (*p* = 0.1558), LTF (*p* = 0.2661) and significantly higher: ce10 (*p* = 0.009) and NPC2 (*p* < 0.0044). These findings may be used to develop diagnostic MRSP-based tests related to ES quality in assisted reproduction techniques in dogs.

## 1. Introduction

Good sperm motility is a key factor determining the success of fertilization, both in natural conditions and in assisted reproduction procedures [1]. In mammals, including dogs, sperm maturation and the acquisition of full motility occur in the epididymis, an integral part of the duct that transports sperm from the testicles [2]. During movement through subsequent sections of the epididymis, sperm undergo several significant morphological, biochemical and functional changes that determine their ability to move and fertilize an egg [3].

Epididymal semen can effectively replace ejaculated semen in circumstances like sudden death of the male, necessary castration, or conditions affecting ejaculation or erection [4]. In recent years, precise reproduction management has become increasingly important in dog breeding. The first important step influencing reproductive outcomes is the evaluation of male semen quality. The methods most commonly used for assessing the quality of both ejaculated and epididymal semen include the assessment of sperm motility; however, in recent years, increasing emphasis has been placed on the search for semen quality markers in the form of high- or low-molecular-weight substances found in semen and related to its quality [5]. Besides various biochemical substances, proteins found in epididymal spermatozoa (ES) significantly influence sperm physiology by controlling the maturation of sperm within the epididymis and facilitating their acquisition of motility [6]. To the best of the authors’ knowledge, only a few sperm proteins that may influence canine sperm motility have been identified. These proteins include A-kinase anchoring protein 4 (AKAP4), albumins (ALBs), metalloproteinase 2 (MMP2), osteopontin (OPN), actin cytoplasmic 1 (ACTB) and cystatin domain-containing protein (LOC607874) [7,8,9,10]. However, a broad proteomic study was first conducted in this research to analyze the full set of proteins with respect to their influence on sperm motility. Examination of canine epididymal semen using high-throughput OMICS (proteomics) and advanced molecular biology tools provides an unusual opportunity to identify proteins with potential utility as biomarkers. Conventional evaluation methods, such as semen analysis alone, may not fully reflect spermatozoa’s functional capacity [11]. Biomarkers, including hormone levels (e.g., testosterone), oxidative stress markers, and molecular indicators of sperm function (not only ejaculated sperm but also epididymal), enable a more precise assessment of reproductive potential [12]. Their application supports the early detection of subfertility or infertility, guides treatment strategies, and improves the success of breeding programs. Furthermore, biomarkers help optimize semen collection, storage, and use in assisted reproductive technologies, thereby improving genetic management and preserving valuable breeding lines [13]. In this study, a semiquantitative proteomic analysis was conducted to determine whether canine ES could serve as a potential source of biomarkers for sperm motility. This study aimed to analyze the proteomic profile of canine ES across groups characterized by different progressive motility values to identify motility-related sperm proteins (MRSPs).

## 2. Materials and Methods

### 2.1. Chemicals and Media

All chemicals, unless specified otherwise, were obtained from Sigma Chemical Company (St. Louis, MO, USA) and were of the highest purity.

### 2.2. Animals

The study involved 19 mixed-breed dogs aged 1–11 years (5.13 ± 0.73, mean ± SEM) and weighing 9–30 kg (19.13 ± 1.45, mean ± SEM). All animals were housed under uniform conditions at the Animal Shelter in Tomaryny, Poland, and fed a consistent, balanced diet. They participated in a welfare initiative focused on reducing stray populations and encouraging adoption. The authors are authorized to conduct animal experiments in accordance with the Polish Laboratory Animal Science Association (Numbers: 1432/2015; 1508/2015). An exemption letter was obtained from the Local Ethics Committee for Animal Experimentation, Olsztyn, Poland (LKE/01/2022). Additionally, a consent form was obtained from the director of the Shelter for Homeless Animals in Tomaryny, Poland. Each dog underwent a routine orchiectomy procedure performed by a qualified veterinarian.

### 2.3. Cauda Epididymal Semen Collection

After surgical removal, the testicles with epididymides from the experimental dogs were placed in sterile plastic bags containing 0.9% NaCl solution and stored in a thermobox at 4 °C. The tissues were delivered to the Department of Animal Biochemistry and Biotechnology at the University of Warmia and Mazury in Olsztyn, Poland, within one hour. Upon arrival, the gonads were thoroughly rinsed with Dulbecco’s phosphate-buffered saline (DPBS; Gibco, Grand Island, NY, USA). Using a sterile scalpel, the cauda epididymal tissue was carefully separated from the testis to prevent vascular damage. Epididymal semen was then aspirated with an automatic pipette, following the method described by Ramos Angrimani et al. [14] with modifications (epididymal semen was collected immediately after tissue delivery to the laboratory, and epididymal semen was re-suspended in DPBS). Samples from the left and right epididymides of each dog were pooled.

### 2.4. Cauda ES Quality Assessment

The ES concentration was measured using a Bürker chamber under a light microscope (Olympus BX41TF, Tokyo, Japan). Sperm motility and motion parameters were evaluated using a computer-assisted semen analysis system (CASA) system (HTM-IVOS 12.3, Hamilton-Thorne Biosciences, MA, USA). The software settings applied were as described in a previous study [15]. The analyzed motility and motion parameters included total motility (TMOT, %), progressive motility (PMOT, %), average path velocity (VAP, µm/s), straight-line velocity (VSL, µm/s), curvilinear velocity (VCL, µm/s), the amplitude of lateral head displacement (ALH, µm), beat cross frequency (BCF, Hz), straightness (STR, %), and linearity (LIN, %) for each ES sample.

Following sperm motility analysis, the samples were categorized into two experimental groups based on their PMOT values: the good sperm motility (GSM) group (n = 10), with a PMOT ≥ 55%, and the poor sperm motility (PSM) group (n = 9), with a PMOT < 55%, as defined by a previously published study [9].

### 2.5. Cluster Analysis

Cluster analysis was performed to visualize dogs of the GSM and PSM groups, using the SRplot (https://www.bioinformatics.com.cn/plot_basic_PCA_plot_034_en, accessed on 16 November 2025 [16]). Moreover, principal component analysis (PCA) was performed to visualize dogs within the sperm motility-based groups with 55% PMOT as the primary classification criterion. The PCA components included TMOT, PMOT, VCL, ALH, BCF, STR and LIN of either the GSM or PSM group.

### 2.6. Preliminary Sample Preparation

Selected epididymal semen samples were subsequently centrifuged at 800× *g* for 10 min at 4 °C to remove the epididymal fluid. The remaining supernatant (epididymal fluid) was discarded, and the resulting sperm pellet (ES) was re-suspended in 1 mL of DPBS and then centrifuged again under the same conditions to eliminate loosely bound proteins [17]. After the final centrifugation, the remaining supernatant was removed. Each ES sample was then divided into two portions: one was used for electrophoretic analysis, and the other was stored at −80 °C for two weeks for subsequent LC–MS analysis.

The ES samples were placed in an ice bath and sonicated with an Omni Sonic Ruptor 250 Ultrasonic Homogenizer (Omni International, Kennesaw, GA, USA) at 150 W for 10 min at 60 kHz. After sonication, the samples were centrifuged at 8000× *g* for 10 min at 4 °C. The resulting supernatant containing the sperm intracellular proteins (SIPs) was transferred to a separate Eppendorf tube. The remaining supernatant was treated with 1 mL of radioimmunoprecipitation assay (RIPA) buffer (composition: 50 mM Tris-HCl, 150 mM NaCl, 1% (*v*/*v*) Triton X-100, 0.5% sodium deoxycholate, 0.1% SDS, and ddH_2_O, pH 7.4). This mixture was incubated at 4 °C for 5 min, vortexed and then left overnight to extract proteins, with slight modifications to the protocols of Intasqui et al. [18] and Mogielnicka-Brzozowska et al. [19].

A protease inhibitor cocktail (catalog number: P8340, Sigma-Aldrich, St. Louis, MO, USA) was added to both the SIPs fraction and the remaining sperm pellets. The samples were subsequently centrifuged at 8000× *g* for 10 min at 4 °C to isolate a clear lysate containing sperm membrane-associated proteins (SMAPs). This lysate was collected into another Eppendorf tube and combined with the SIPs to form a complete ES protein extract (referred to as sperm extracts), which was then stored at −80 °C until further analysis.

### 2.7. Sample Preparation for Proteomic Analysis

#### 2.7.1. Sample Preparation for NanoUPLC-Q-TOF/MS

The total protein content in the ES was measured using Bradford reagent (catalog number: B6916). To obtain lipid-free protein extracts, frozen samples were thawed in 800 µL of a chilled (–18 °C) methyl tert-butyl ether-methanol mixture (3:1, *v*/*v*). The samples were then sonicated in an ice–water bath for 15 min. Next, 400 µL of water-methanol solution (3:1, *v*/*v*) was added to each tube to precipitate the proteins. The samples were subsequently centrifuged at 21,000× *g* for 7 min at 4 °C. The supernatants were transferred to new tubes, and the protein pellets were stored at −80 °C for later use.

#### 2.7.2. In-Solution Trypsin Digestion

Protein digestion was performed according to the protocol of Morelle and Michalski [20], with minor modifications. The protein pellets were re-suspended in 100 µL of 50 mM ammonium bicarbonate (NH_4_HCO_3_, pH 8.0) containing 0.1% sodium dodecyl sulfate. Freshly prepared dithiothreitol solution was added to reach a final concentration of 20 mM, and the samples were incubated at 60 °C for one hour. Subsequently, iodoacetamide was added to a final concentration of 20 mM, and the samples were incubated in the dark for another hour. The mixture was diluted twice, and 5 µg of trypsin was added for overnight digestion at 37 °C with gentle shaking. After digestion, 1% formic acid was added to adjust the pH to 3–4. The samples were desalted using Pierce™ C18 columns (Thermo Fisher Scientific, Waltham, MA, USA), vacuum-dried and re-suspended in acetonitrile for subsequent LC–MS analysis.

### 2.8. NanoUPLC-Q-TOF/MS

For each individual in the GSM and PSM groups, proteomic profiling was performed using NanoUPLC-Q-TOF/MS. Protein fractionation was achieved through the Waters Acquity M-Class UPLC system (Waters Corp., Milford, MA, USA), fitted with a Peptide BEH C18 analytical column (150 mm × 75 µm, 1.7 µm, Waters Corp.) and a Symmetry C18 precolumn (180 µm × 20 mm, 1.7 µm, Waters Corp.). Each sample was initially loaded onto the precolumn and rinsed with 99% solvent A (0.1% formic acid in water) at 5 µL/min for 5 min. Subsequently, the peptides were transferred onto the analytical column for chromatographic separation. The mobile phase was maintained at a constant flow rate of 300 nL/min. The complete analytical gradient, including the equilibration phase, lasted 75 min. The elution was performed according to the following gradient steps:

- from 0 to 2 min: 5% B (0.1% formic acid in acetonitrile);
- from 2 to 15 min: 5% to 30% B;
- from 15 to 45 min: 30% to 60% B;
- from 45 to 48 min: 60% to 85% B;
- from 45 to 58 min: held at 85% B;
- from 58 to 58.5 min: reduced back to 5% B.

Mass spectrometry was performed on a Synapt G2-Si system (Waters Corp.) equipped with a nano-electrospray ionization source operating in positive-ion mode. The setup parameters included a capillary voltage of 3.0 kV, a cone voltage of 40 V, a cone gas flow of 40 L/h, a source temperature of 100 °C, and a nanoflow gas pressure of 0.2 bar. Data were collected in data-independent acquisition mode across an m/z range of 70–1800. Leucine enkephalin (*m*/*z* 556.2771) served as the reference compound for lockspray calibration, with measurements taken every 45 s and real-time mass correction applied automatically.

Chromatographic data were processed using Byonic software v. 4.4 (Protein Metrics, Cupertino, CA, USA). Peptide identification was performed under the following parameters: trypsin digestion, maximum two miss-cleavages, and maximum three charges. The resulting peptide spectra were matched against the canine proteome database (SWISSPROT, CANLF), downloaded in February 2022. To minimize false positives, data were filtered by comparison with common contaminants and decoy sequences generated through reverse amino acid modeling of theoretical peptide cleavages.

Overall protein intensity was calculated as the sum of all peak intensities from MS/MS spectra. Protein-level statistical confidence was expressed as *p*-values representing the probability of peptide–spectrum matches occurring by random chance under a probabilistic model. For instance, a log *p*-value of −3.0 corresponds to a protein *p*-value of 0.001, or one chance in a thousand.

### 2.9. Bioinformatic Analyses and Imaging of Data

The functional enrichment of proteins present in the canine (*Canis lupus familiaris*) ES was analyzed in the GSM and PSM groups according to the following GO categories: molecular function (MF), biological process (BP), cellular component (CC), and protein class (PC), with the PANTHER Classification System v. 19.0 online tool (http://pantherdb.org, accessed on 19 August 2025). ES motility and motion parameters plots, GO plots, proteins contribution plots, box plots, Western blot plot and heatmaps were performed using GraphPad PRISM v. 10.4.2 software (GraphPad Software, Boston, MA, USA).

### 2.10. Western Blot Analysis

Proteins extracted from the sperm, each containing 50 µg of protein, were separated using 12% Sodium Dodecyl Phosphate–Polyacrylamide Gel Electrophoresis (SDS–PAGE) and subsequently transferred onto Immobilon-P polyvinylidene fluoride (PVDF) membranes (Millipore, Bedford, MA, USA). Semi-dry electrotransfer was performed at 150 mA for 1 h, as previously described [21]. To block nonspecific binding sites, the membranes were incubated in Tris-buffered saline (1 M Tris, 5 M NaCl, pH 8.0) containing 0.05% Tween 20 (MP Biomedicals LLC, Santa Ana, CA, USA) supplemented with 5% nonfat dry milk. The membranes were then incubated overnight at 4 °C with one of the following primary antibodies:

- Bcl-10 rabbit polyclonal antibody (catalog number: PA5-88067, Thermo Fisher Scientific; dilution 1:500; the gene name Bcl-10 was used as the alias name for ce10, according to the manufacturer’s information, it is an antibody that recognizes ce10),
- NPC2 rabbit polyclonal antibody (catalog number: PA5-51463, Thermo Fisher Scientific; dilution 1:500), and,
- rabbit polyclonal anti-GAPDH antibody (catalog number: G9545, Sigma-Aldrich; dilution 1:2000), as a loading control.

The membranes were then washed and incubated with Peroxidase AffiniPure Goat Anti-Rabbit secondary antibody (catalog number: 111-035-003, Jackson ImmunoResearch, Baltimore Pike, PA, USA; dilution 1:20,000) at room temperature for one hour. Chemiluminescence was developed using the ServaLight CL EOS Substrate Kit (Serva, Heidelberg, Germany), and protein bands were visualized using the ChemiDoc™ Touch Imaging System (Bio-Rad Laboratories, Hercules, CA, USA). Molecular weights were estimated using PageRuler™ Prestained Protein Ladder (catalog number: 26617, Thermo Fisher Scientific). Band intensities were quantified using Image Lab version 5.2 (Bio-Rad Laboratories), and signals were normalized to the control GAPDH expression level.

### 2.11. Statistical Analyses

GraphPad PRISM v. 10.4.2 software (GraphPad Software) was used to perform the statistical analyses. Student’s *t*-test was used to analyze differences between the GSM and PSM groups for PMOT, VAP, VSL, VCL, ALH, STR, and LIN values, while TMOT and BCF values were analyzed with the Mann–Whitney U test for pairwise comparisons. Differences in the abundance of proteins between the GSM and PSM groups were analyzed either with Student’s *t*-test or the Mann–Whitney U test. Correlations between the relative abundance (MS Intensity) of cauda ES proteins and the sperm motility and motion parameters were evaluated using Spearman’s rank correlation coefficient. Values were considered significant at *p* < 0.05.

## 3. Results

### 3.1. Cluster Analysis

**Figure 1:**
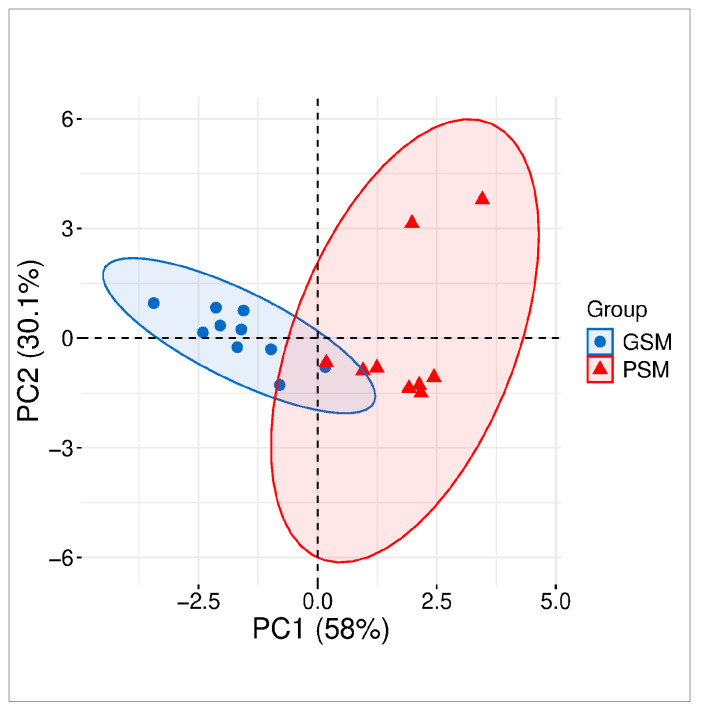
Components of principal component analysis (PCA) of parameters of motility and motion of epididymal canine sperm. Values of the analyzed parameters of sperm from dogs with good and poor sperm motility (GSM and PSM, respectively). Components included total motility (TMOT), progressive motility (PMOT), curvilinear velocity (VCL), amplitude of lateral head displacement (ALH), beat cross frequency (BCF), straightness (STR) and linearity (LIN) of either the GSM or PSM group.

Cluster analysis of the first two components showed that 88.1% of the total variance in the analyzed ES parameters in dogs was attributable to the GSM and PSM groups. Principal component analysis 1 (PCA1) could explain 58% of the total variance, whereas PCA2 confirmed 30.1% of the total variance between the GSM and PSM groups (Figure 1).

### 3.2. ES Motility and Motion Parameters in Research Groups

**Figure 2:**
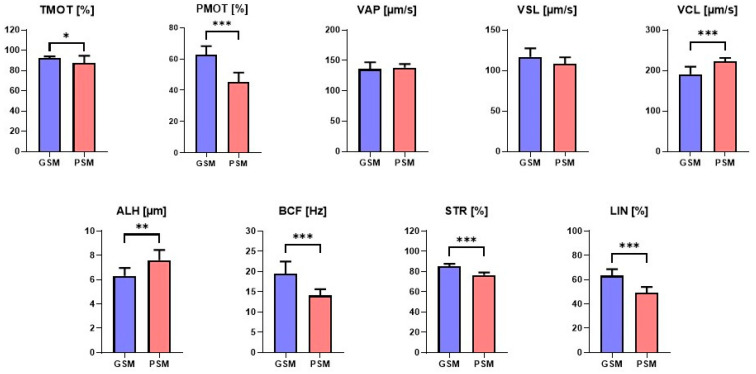
Motility and motion parameters of the epididymal spermatozoa (ES) of dogs with good sperm motility (GSM) and poor sperm motility (PSM). The values are presented as the means ± SEMs. Significant differences with *p* < 0.05 *, *p* < 0.01 ** and *p* < 0.001 ***. The abbreviations for the sperm motility and motion parameters are explained in Section 2.4.

Canine epididymal sperm motility and motion parameters were compared between the GSM and PSM groups. Significant statistical differences were demonstrated between the GSM and PSM groups for TMOT (*p* = 0.039). For the traits: PMOT (*p* < 0.001), VCL (*p* < 0.001), ALH (*p* = 0.001), BCF (*p* < 0.001), STR (*p* < 0.001), and LIN (*p* < 0.001), highly statistically significant differences were observed. There were no statistically significant differences (*p* < 0.05) between the GSM and PSM groups for VAP (*p* = 0.619) or VSL (*p* = 0.88) (Figure 2).

### 3.3. Whole ES Proteome Profiling According to Research Group

Whole proteome analysis of canine ES revealed the presence of 366 polypeptides in the GSM group and 292 in the PSM group. Moreover, 244 unique proteins (UPs) were identified in the GSM group, whereas 170 UPs were found in the PSM group. In addition, 122 polypeptides were common proteins (CPs) present in both study groups. The proteins identified in each research group are listed in Supplementary Tables S1 and S2.

#### 3.3.1. Protein Contribution in GSM and PSM Groups

The analysis focused on the percentages of individual proteins in canine ES that were ≥1% in the GSM and PSM groups. Among the canine ES proteins identified in the GSM group, the highest percentages were as follows: lactotransferrin (LTF, 18%), actin cytoplasmic 1 (ACTB, 6%), prostaglandin-H2 D-isomerase (PTGDS, 5%), abnormal spindle-like microcephaly-associated protein homolog (ASPM, 5%), CE10 protein (ce10, 3%), 60S ribosomal protein L32 (RPL32, 3%), NPC intracellular cholesterol transporter 2 (NPC2, 3%), albumin (ALB, 3%), WAP domain-containing protein (WAPdcp*, 2%), alpha fetoprotein (AFP, 2%), sacsin molecular chaperone (SACS, 2%) and structural maintenance of chromosomes protein (SMC1B, 2%).

**Figure 3:**
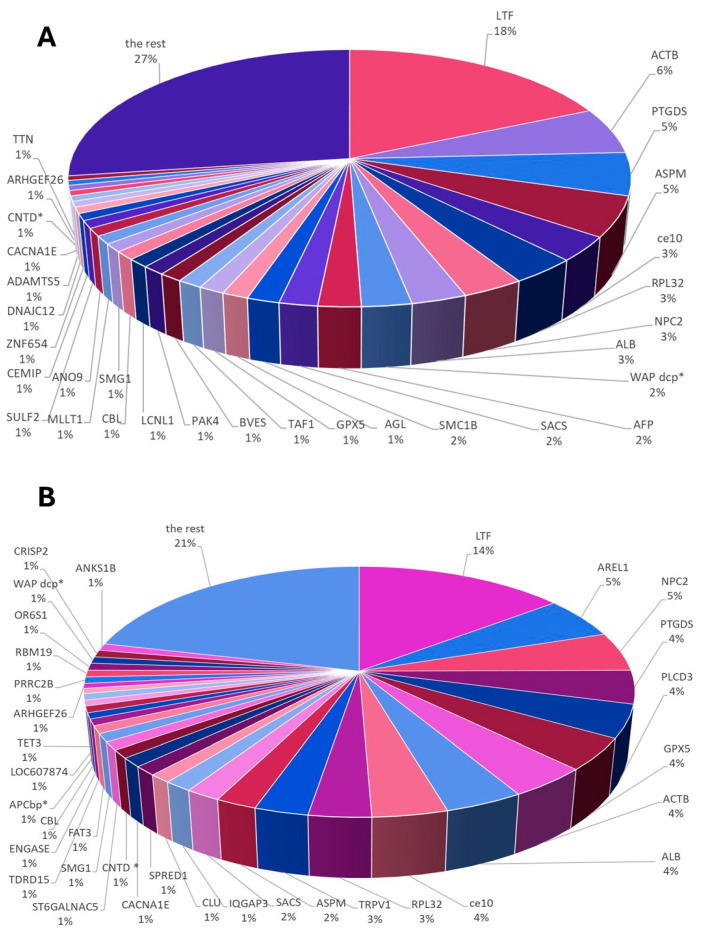
Graph showing the percentage of proteins that comprised ≥1% of the total proteins identified in the epididymal spermatozoa (ES) of dogs with (**A**) good sperm motility (GSM) and (**B**) poor sperm motility (PSM). Due to the difficulty of visualizing such a large number of proteins, the graphs show selected proteins that constitute ≥1% of the total protein pool in the study groups. An asterisk next to a protein abbreviation indicates that no official abbreviation was found in the reference databases; such abbreviations were introduced by the authors for this publication. Proteins ≤ 1% of the total protein amount (the rest) were presented in Supplementary Tables S3 and S4.

The group of GSM proteins, which constituted 1% of the entire pool of dog epididymal proteins, includes glycogen debranching enzyme (AGL, 1%), glutathione peroxidase (GPX5, 1%), transcription initiation factor TFIID subunit (TAF1, 1%), blood vessel epicardial substance (BVES, 1%), non-specific serine/threonine protein kinase (PAK4, 1%), lipocln_cytosolic_FA-bd_dom domain-containing protein (LCNL1, 1%), E3 ubiquitin-protein ligase CBL (CBL, 1%), non-specific serine/threonine protein kinase (SMG1, 1%), MLLT1 super elongation complex subunit (MLLT1, 1%), anoctamin (ANO9, 1%), sulfatase 2 (SULF2, 1%), hyaluronoglucosaminidase (CEMIP, 1%), zinc finger protein 654 (ZNF654, 1%), J domain-containing protein (DNAJC12, 1%), ADAM metallopeptidase with thrombospondin type 1 motif 5 (ADAMTS5, 1%), voltage-dependent R-type calcium channel subunit alpha (CACNA1E, 1%), cyclin N-terminal domain-containing protein (CNTD*, 1%), Rho guanine nucleotide exchange factor 26 (ARHGEF26, 1%), and titin (TTN, 1%) (Figure 3A).

In the GSM group, when analyzing 244 UPs, seven proteins accounted for ≥1% of the total protein content in canine ES. These were AFP (2%), TAF1 (1%), BVES, MLLT1, ANO9, ZNF654, and TTN (Figure 3A).

Among the canine ES proteins identified in the PSM group, the highest percentages were LTF (14%), apoptosis-resistant E3 ubiquitin protein ligase 1 (AREL1, 5%), NPC2 (5%), PTGDS (4%), phosphoinositide phospholipase C (PLCD3, 4%), GPX5 (4%), ACTB (4%), ALB (4%), ce10 (4%), RPL32 (3%), transient receptor potential cation channel subfamily V member 1 (TRPV1, 3%), ASPM (2%) and SACS (2%).

The group of PSM proteins, which constituted 1% of the entire pool of dog epididymal proteins, includes: IQ motif containing GTPase activating protein 3 (IQGAP3, 1%), clusterin (CLU, 1%), sprouty related EVH1 domain containing 1 (SPRED1, 1%), CACNA1E (1%), CNTD* (1%), ST6 N-acetylgalactosaminide alpha-2,6-sialyltransferase 5 (ST6GALNAC5, 1%), SMG1 (1%), FAT atypical cadherin 3 (FAT3, 1%), tudor domain containing 15 (TDRD15, 1%), mannosyl-glycoprotein endo-beta-N-acetylglucosaminidase (ENGASE, 1%), CBL (1%), APC-binding protein EB1 (APCbp*, 1%), cystatin domain-containing protein (LOC607874, 1%), methylcytosine dioxygenase TET (TET3, 1%), ARHGEF26 (1%), proline rich coiled-coil 2B (PRRC2B, 1%), RNA binding motif protein 19 (RBM19, 1%), olfactory receptor (OR6S1, 1%), WAPdcp* (1%), cysteine-rich secretory protein 2 (CRISP2, 1%), ankyrin repeat and sterile alpha motif domain containing 1B (ANKS1B, 1%) (Figure 3B).

In the PSM group, when analyzing 170 UPs, eight proteins accounted for ≥1% of the total protein content in canine ES. These were AREL1 (5%), IQGAP3 (1%), SPRED1 (1%), ST6GALNAC5 (1%), FAT3 (1%), APCbp* (1%), OR6S1 (1%) and ANKS1B (1%) (Figure 3B).

The exact sets of proteins that constitute <1% in both the GSM and PSM groups are presented in Supplementary Tables S3 and S4.

An asterisk next to a protein abbreviation indicates that no official abbreviation was found in the reference databases; such abbreviations were introduced by the authors for this publication.

**Figure 4:**
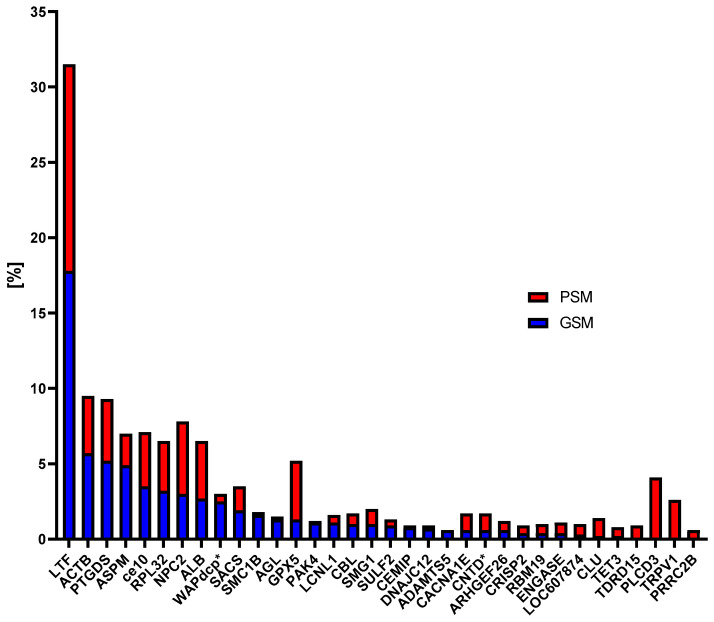
Comparison of the percentage of common proteins (CPs) in epididymal spermatozoa (ES) of dogs with good sperm motility (GSM) and poor sperm motility (PSM). LTF—lactotransferrin, ACTB—actin cytoplasmic 1, PTGDS—prostaglandin-H2 D-isomerase, ASPM—abnormal spindle-like microcephaly-associated protein homolog, ce10—CE10 protein, RPL32—60S ribosomal protein L32, NPC2—NPC intracellular cholesterol transporter 2, ALB—albumin, WAPdcp*—WAP domain-containing protein, SACS—sacsin molecular chaperone, SMC1B—structural maintenance of chromosomes protein, AGL—glycogen debranching enzyme, GPX5 –glutathione peroxidase, PAK4—non-specific serine/threonine protein kinase, LCNL1—lipocln_cytosolic_FA-bd_dom domain-containing protein, CBL—E3 ubiquitin-protein ligase CBL, SMG1—non-specific serine/threonine protein kinase, SULF2—sulfatase 2, CEMIP—hyaluronoglucosaminidase, DNAJC12—J domain-containing protein, ADAMTS5—ADAM metallopeptidase with thrombospondin type 1 motif 5, CACNA1E—voltage-dependent R-type calcium channel subunit alpha, CNTD*—cyclin N-terminal domain-containing protein, ARHGEF26—Rho guanine nucleotide exchange factor 26, CRISP2—cysteine-rich protein secretory protein 2, RBM19—RNA binding motif protein 19, ENGASE—mannosyl-glycoprotein endo-beta-N-acetylglucosaminidase, LOC607874—cystatin domain-containing protein, CLU—clusterin, TET3—methylcytosine dioxygenase TET, TDRD15—tudor domain containing 15, PLCD3—phosphoinositide phospholipase C, TRPV1—transient receptor potential cation channel subfamily V member 1, PRRC2B—proline rich coiled-coil 2B. An asterisk next to a protein abbreviation indicates that no official abbreviation was found in the reference databases; such abbreviations were introduced by the authors for this publication.

For both study groups of dogs, 122 CPs were identified. Despite their presence in both study groups, these proteins exhibited varying levels of content between the groups (GSM/PSM). The highest content was observed for the following proteins: LTF (18%/14%), ACTB (6%/4%), PTGDS (5%/4%), ASPM (5%/2%), and ce10 (3%/4%) (Figure 3). Comparing the CPs content for both study groups, statistically significant differences were found for ce10 (GSM—1093 ± 248.2 × 10^5^, PSM—391.4 ± 209.6 × 10^5^; *p* = 0.028), NPC2 (GSM—1857 ± 327.6 × 10^5^, PSM—281 ± 96.7 × 10^5^; *p* = 0.0044) and PAK4 (GSM—704.2 ± 189.1 × 10^5^, PSM—30.2 ± 11.7 × 10^5^; *p* = 0.0369) (Figure 4). Values are presented as mean ± SEM. For all three proteins, the content was higher in the GSM group.

#### 3.3.2. Gene Ontology

**Figure 5:**
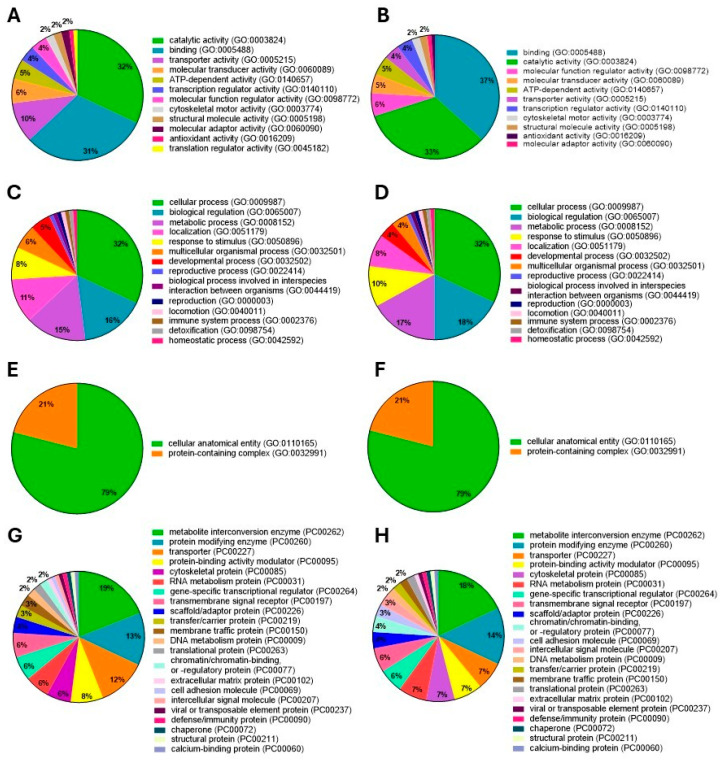
GO enrichment of the proteins present in the epididymal spermatozoa (ES) of dogs with good sperm motility (GSM) and poor sperm motility (PSM). The following GO categories were distinguished: molecular function (MF)—GSM (**A**), PSM (**B**); biological process (BP)—GSM (**C**), PSM (**D**); cellular components (CC)—GSM (**E**), PSM (**F**); and protein class (PC)—GSM (**G**), PSM (**H**).

Based on the gene ontology (GO) analysis, similar molecular functions (MFs) were observed in both groups, GSM and PSM. In the case of GSM proteins, MFs were dominated by catalytic activity (32% of proteins) and binding (31% of proteins) (Figure 5A). Among the ES proteins in the PSM group, MFs accounted for 37% of proteins with binding function, 33% showed catalytic activity, and 6% acted as molecular function regulators (Figure 5B).

According to the GO analysis, similar biological processes (BPs) were observed in both the GSM and PSM groups, although the percentages differed slightly. For proteins of both study groups, BPs were dominated by cellular processes (GSM and PSM, 32%). Additionally, the proteins were shown to participate in biological regulation (GSM—16%, PSM—18%) and in metabolic processes (GSM—15%, PSM—17%) (Figure 5C,D).

GO analysis revealed the same cellular component (CC) terms for both study groups, GSM and PSM, with no difference in percentage. In both groups, CCs were dominated by the cellular anatomical entity group, which accounted for 79% of proteins, and the protein-containing complex group accounted for 21% (Figure 5E,F).

Based on the GO analysis, similar terms were shown in the protein class (PC) category for both GSM and PSM groups. In the case of GSM, 19% of the proteins were classified as metabolite interconversion enzymes, 13% as protein-modifying enzymes, and 12% as transporters (Figure 5G). Among the ES proteins in the PSM, in the PC category, 18% were metabolite interconversion enzymes, and 14% were protein-modifying enzymes (Figure 5H).

### 3.4. Selection of Potential MRSPs

The presence of a given protein in the study group was considered significant if it met the criterion of being present in at least 50% of individuals plus 1 animal. The analysis revealed five CPs in both study groups, five UPs in the GSM group and no UPs in the PSM group.

#### 3.4.1. Analysis of the CPs Content in the Research Groups

**Figure 6:**
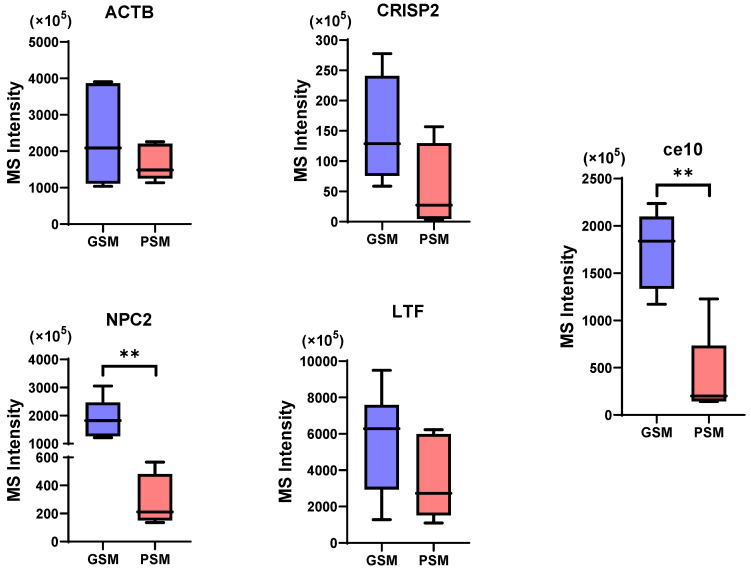
Box plots representing the content (MS Intensity) of common proteins (CPs) for both good sperm motility (GSM) and poor sperm motility (PSM). ACTB—actin cytoplasmic 1, ce10—CE10 protein, CRISP2—cysteine-rich secretory protein 2, LTF—lactotransferrin, NPC2—NPC intracellular cholesterol transporter 2. Statistically significant differences at *p* < 0.01 ** were shown, respectively.

Five proteins were observed in both examined groups. These proteins included actin cytoplasmic 1 (ACTB), CE10 protein (ce10), cysteine-rich secretory protein 2 (CRISP2), lactotransferrin (LTF) and NPC intracellular cholesterol transporter 2 (NPC2). The values are presented as the means ± SEMs. For all of the above proteins, their content was higher in the GSM group than in the PSM group. Two proteins were significantly different: ce10 (GSM—1754 ± 168.4 × 10^5^, PSM—391.4 ± 209.6 × 10^5^; *p* = 0.009) and NPC2 (GSM—1857 ± 327.6 × 10^5^, PSM—281 ± 96.72 × 10^5^; *p* = 0.0044). The remaining three proteins, ACTB (GSM—2347 ± 453.4 × 10^5^, PSM—1681 ± 222.3 × 10^5^; *p* = 0.2732), CRISP2 (GSM—148.5 ± 46.11 × 10^5^, PSM—53.78 ± 35.77 × 10^5^; *p* = 0.1558) and LTF (GSM—5425 ± 1121 × 10^5^, PSM—3548 ± 1034 × 10^5^; *p* = 0.2661), differed in content between groups, but the differences were not statistically significant (Figure 6).

#### 3.4.2. Validation of Potential MRSPs

**Figure 7:**
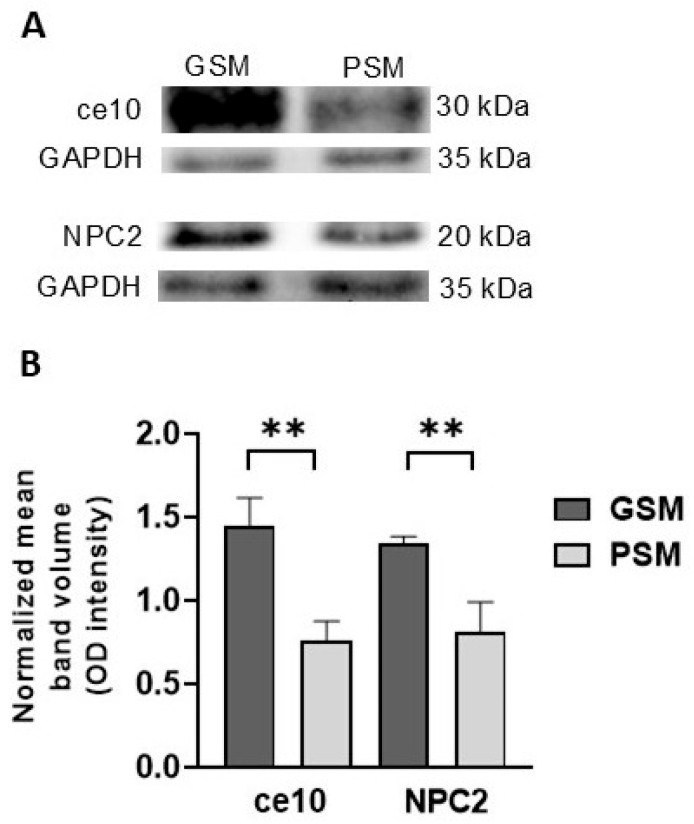
The Western blot assay was used to confirm differences in the epididymal sperm (ES) protein levels between dogs exhibiting good sperm motility (GSM) and poor sperm motility (PSM) (**A**). Each column displays the normalized mean ± SEM of protein abundance calculated from three independent replicates. Protein levels, represented by band intensity, differed significantly between the groups analyzed. A significant difference between GSM and PSM was detected at *p* < 0.01 ** (**B**). ce10—CE10 protein; NPC2—NPC intracellular cholesterol transporter 2; GAPDH—Glyceraldehyde-3-phosphate dehydrogenase.

To confirm the presence and relative abundance of the proteins detected by mass spectrometry, Western blot analysis was performed. The analysis revealed significantly higher abundances of ce10 (*p* = 0.0042) and NPC2 (*p* = 0.0076) in the GSM group than in the PSM group (Figure 7), confirming the LC–MS results.

#### 3.4.3. Correlation Between the CPs Content and ES Motility

**Figure 8:**
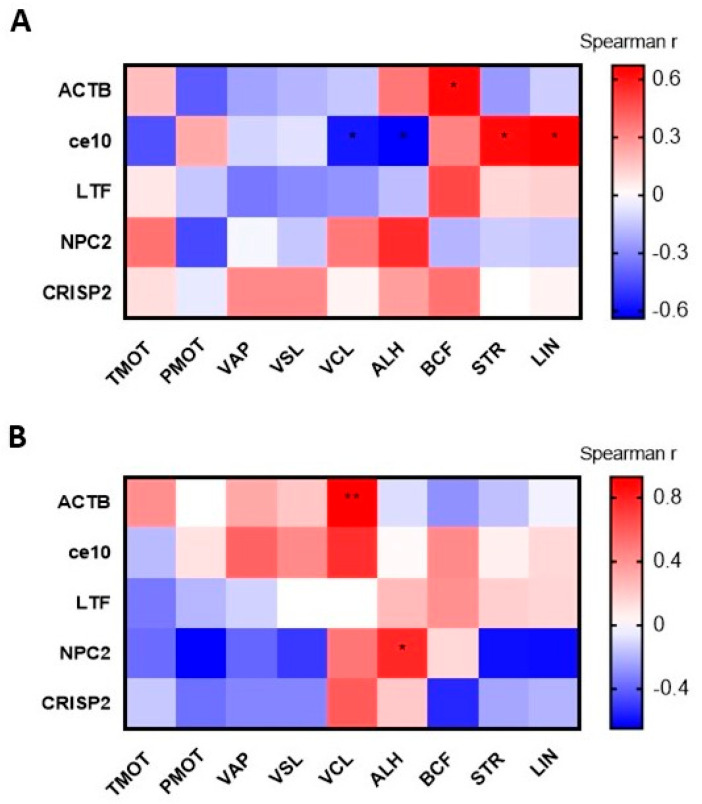
Heatmap showing Spearman’s rank correlation coefficients between the values of the dog epididymal sperm (ES) motion parameters and the abundance (MS intensity) of common proteins (CPs) for good sperm motility (GSM) group (**A**) and poor sperm motility (PSM) group (**B**). Significant correlation * *p* < 0.05; ** *p* < 0.01. ACTB—actin cytoplasmic 1; ce10—CE10 protein; LTF—lactotransferrin; NPC2—NPC intracellular cholesterol transporter 2; CRISP2—cysteine-rich secretory protein 2. The abbreviations for the sperm motility and motion parameters are explained in Section 2.4.

Correlation analysis between dog ES motility parameters and the content of CPs in the two groups revealed positive correlations in the GSM group for ACTB and BCF (r = 0.6364; *p* = 0.0402), but also for ce10 and STR (r = 0.6290; *p* = 0.0317) and ce10 and LIN (r = 0.6714; *p* = 0.0197) (Figure 8A). Additionally, negative correlations in GSM group were found for ce10 and VCL (r = −0.5734; *p* = 0.0496) and ALH (r = −0.6386; *p* = 0.0288) (Figure 8A). In the PSM group, positive correlations were found between ACTB and VCL (r = 0.9286; *p* = 0.0067) and NPC2 and ALH (r = 0.7748; *p* = 0.0492) (Figure 8B).

## 4. Discussion

The analysis and identification of protein biochemical properties play important roles in the study of mechanisms that influence sperm functions, including motility, and thus reproductive processes [1]. The identification of proteins as MRSPs has great potential, as it helps elucidate the relationship between the biochemical components that build sperm and sperm motility quality [5]. The study revealed the presence of unique proteins (UPs) in dog ES, which were only detected in one of the research groups (GSM or PSM). This finding indicates the presence of a pool of characteristic proteins associated with the progressive motility of ES.

In the GSM group, 244 UPs were identified, seven of which accounted for at least 1% of the total protein content. These proteins were AFP, TAF1, BVES, MLLT1, ANO9, ZNF654 and TTN. AFP is a serum glycoprotein with structural and physicochemical properties similar to those of albumin [22]. This protein was previously found in canine epididymal fluid, sperm and human fetal serum [9,15,22]. AFPs can bind to various membrane receptors and intracellular proteins, influencing the activation or inhibition of signaling pathways. As a result, it can modulate cell growth both in fetal life and during tumor formation, where it can act as an amplifier or an inhibitor [23,24,25,26,27]. In a study by Cichowska et al. [9] on the composition of canine epididymal fluid, AFP was a CP in both groups with good and poor sperm motility, with the protein content in the group with good motility being higher [9].

The presence of MLLT1 has previously been demonstrated in the canine ES proteome [15]. Analysis of metabolic pathways using the Kyoto Encyclopedia of Genes and Genomes (KEGG) revealed this protein’s involvement in abnormal transcriptional regulation in cancer development [15].

The remaining proteins, TAF1, BVES, ANO9, ZNF654, and TTN, were identified in the above studies for the first time in dog ES, and their functions in processes related to mammalian reproduction, including in dogs, remain unestablished. Determining their positive effects on canine ES motility requires further detailed analysis.

In the current study, 170 UPs were observed in the PSM group. Of these, eight accounted for at least 1% of the total protein content. These proteins were AREL1, IQGAP3, SPRED1, ST6GALNAC5, FAT3, APCbp*, OR6S1 and ANKS1B.

AREL1 was previously identified in canine ES, and its content is age-dependent on the age of the individual [15]. It belongs to a subfamily of proteins that inhibit apoptosis by ubiquitinating and degrading of proapoptotic proteins [28]. AREL1 acts as a general inhibitor of apoptosis in cancer cells induced by various stimuli [29]. The available literature remains insufficient to explain the direct effect of this protein on sperm motility.

The remaining proteins, IQGAP3, SPRED1, ST6GALNAC5, FAT3, APCbp*, OR6S1, and ANKS1B, were identified for the first time in dog ES, and their functions in mammalian reproduction processes, including in dogs, remain unknown. Understanding their potential negative effects on the motility of canine ES requires further detailed analyses.

The conducted studies identified 122 CPs that were present in both research groups, which differed in motility. Their dominant presence in the canine ES proteome was demonstrated, and their percentage varied across research groups. The most abundant proteins were LTF, ACTB, PTGDS, ASPM and ce10. It should be emphasized that two of the above proteins, LTF and PTGDS, are well-known regulators of sperm physiology and their key functional roles in sperm functions have been widely described in a comprehensive review by Mogielnicka-Brzozowska and Cichowska [5]. The presence of CPs in both research groups may indicate that not only the mere presence of a given protein is important but also its appropriate concentration in ES, which determines its final effect on sperm motility.

The following section describes the remaining three proteins: ACTB, ce10 and ASPM.

ACTB is a structural protein of sperm cells that forms the cytoskeleton and is present in the flagellar and acrosomal membranes, where it contributes to cell volume regulation [30,31,32]. Due to its localization, ACTB has been proposed to play a role in sperm capacitation and motility [30,31,32]. In dogs, ACTB has been identified in ES, with expression levels associated with age [15], and it has also been detected in epididymal fluid, where higher abundance is linked to good sperm motility [9]. Despite these observations, the mechanisms by which sperm- or fluid-associated ACTB influences sperm motility remain to be elucidated.

The homolog of the protein associated with spindle-shaped microcephaly (ASPM) is a protein that is structurally and functionally associated with the microtubules of cells. It plays an important role in the control and organization of the mitotic spindle during mouse oocyte meiosis [33]. This protein is localized in the canine ES and is related to the dog’s age [15]. A lack of functional ASPM may affect chromosome segregation accuracy, reducing the ability of fetal stem cells to produce neurons [34]. ASPM also plays a role in sperm tail function, likely influencing sperm motility [35].

The GO analysis indicated only minor differences in the percentage of proteins in specific GO categories between the GSM and PSM groups. However, these results may suggest that sperm motility differentiation relies not only on the presence and activity of specific ES proteins but also on the interactions among these proteins. Such interactions may play a key role in regulating reproductive processes, affecting sperm motility and fertilization.

The study results indicate that the contents of the NPC2, ce10, ACTB, LTF, and CRISP2 proteins were higher in the GSM group than in the PSM group, although statistically significant differences were observed only for NPC2 and ce10, suggesting that these proteins may be MRSPs. The higher contents of these two proteins in the GSM group were confirmed by Western blot analysis. A summary of the functions of the above-mentioned proteins in reproductive processes is shown in Table 1.

NPC2 is also called epididymal secretory protein E1. It is involved in the efflux of cholesterol from lysosomes. Its abnormal function manifests itself in humans with fatal Niemann Pick disease type C2 [36]. This disease is characterized by many neurological symptoms, such as visual palsy, dysarthria, dysphagia or progressive dementia [37]. NPC2 was found in the acrosomal and equatorial regions of the human sperm [38]. This protein contains a cholesterol-binding site and has a strong affinity for cell membrane lipids. Cholesterol is an important component of cell membranes, maintaining their stability and appropriate fluidity [39]. Additionally, it protects sperm from cold shock during cryopreservation [40]. The influence of the NPC2 protein on the efflux of cholesterol from the sperm membrane during its maturation in the mice epididymis has been demonstrated [41]. This phenomenon affects the fluidity of the sperm cytoplasmic membrane [42]. NPC2 has been identified as a potential marker of good sperm freezing because of its ability to prevent the loss of membrane lipids by cryopreserved boar sperm. Therefore, this is a condition for maintaining their viability and motility [43]. According to the above information, NPC2’s ability to regulate cholesterol content in the cell membranes of canine ES may be crucial for regulating sperm motility, and its high amount is desirable.

Ce10 protein is not well known. Canine epididymal proteins occur in the canine epididymis and are homologs of the HE (human epididymal) proteins [44,45]. Ce10 is a glycoprotein identified in the epithelium of the distal part of the epididymis, where it is assumed to participate in sperm maturation as a protease inhibitor [46,47]. The pattern of cysteine residues indicates that ce10 is a four-disulphide core protein, similar to the epididymal CE4 protein [47] but with only a single whey acidic protein (WAP) domain. WAP-domain proteins are secretory proteinase inhibitors. Ce10 has significant antibacterial, antiviral and antifungal effects [48]. To date, no direct impact of the ce10 protein on the motility of dog ES has been reported. According to a recent study, it was not detected in canine epididymal fluid [9], suggesting it may be part of the ES structure. It was found that its low amount correlates with ES aging in dogs [15]. Due to the fact that in other species the homolog of this protein is secreted by the epididymal tissue, it is unclear whether, in dogs, the ce10 protein coats the sperm surface, originates from the epididymal tissue, or is produced by the sperm. Ce10 may be one of the proteinase inhibitors that regulate motility and is build-in ES. However, proteinase inhibitors are well-known substances important for regulation of sperm motility [49]. A comprehensive understanding of the role of proteolysis in male fertilization success and other reproductive processes remains lacking.

Correlation analysis demonstrated a positive association between NPC2 protein content and the ALH parameter. It is difficult to explain this association, but lateral deflection of the sperm head (ALH) is positively correlated with fertilization rates in human sperm, suggesting its potential as a marker of the percentage of zygotes formed [50]. Relationships between the VCL, ALH, and BCF parameters and fertility in pigs have been reported [51,52]. Detailed information on these sperm motility parameters in dogs has not been widely reported in the literature.

The current research, like any other, has certain limitations. The number of individuals included in the study may appear modest, but it is acceptable for proteomic analyses. A small sample size may sometimes limit the statistical power and generalizability of the findings. A minor drawback is that the dogs included in the study represented different breeds, ages, and reproductive histories. This may influence sperm quality and proteomic profiles, thereby introducing biological variability. However, cluster analysis of the sperm parameters showed that dogs’ age and breed did not significantly influence the results. Cluster analysis of the first two components accounted for 88.1% of the total variance in the analyzed ES parameters between the GSM and PSM groups. It is worth considering that dogs used in research differ significantly from farm animals, and obtaining a sufficiently large, age-matched pool of purebred dogs for studies requiring castration is often difficult or even impossible.

It should be stressed that even though a plethora of proteins have been suggested to be involved in the different functions of epididymal or ejaculated sperm, there is still a lack of studies, particularly on proteins in canine ES, regarding the defined roles of these proteins in the sperm motility mechanism. Therefore, future research is planned to elucidate the relevance of potential MRSPs to epididymal functions, including motility and fertilization-related events. Additionally, the identified potential MRSPs require further detailed studies to confirm their suitability as biomarkers of the quality of dog ES. The authors hope that these findings will fill a knowledge gap regarding the canine ES proteome and its potential as a source of biomarkers of ES quality. In the future, MRSPs may be used to develop simple diagnostic tests for ES quality to support assisted reproduction techniques in dogs.

**Table 1:** Summary of epididymal sperm (ES) common proteins (CPs) functions in reproductive processes.

Protein	Functions in Reproductive Processes	References
ACTB (Actin, cytoplasmic 1)	- detected in epididymal sperm and epididymal fluid - involves in sperm capacitation and motility - potential marker of canine epididymal sperm aging - highly abundant in epididymal fluid of dogs with good sperm motility	[9,15,30,31,32]
ce10 (CE10 protein)	- detected in epididymis - participates in sperm maturation and motility as protease inhibitor - possesses significant antibacterial, antiviral and antifungal effects - belongs to epididymal sperm structure - its low amount correlate with epididymal sperm aging in dogs	[9,15,44,45,46,47,48,49]
CRISP2 (Cysteine-rich secretory protein 2)	- detected in testis, epididymal sperm and ejaculated spermatozoa - specifically regulates calcium flow through ryanodine receptors - is implicated in cell–cell adhesion and is capable of steroid binding - decreases in CRISP2 content in sperm is associated with infertility in humans and horses - its content is low in the epididymal sperm of very young and senile dogs	[15,53,54,55,56,57,58,59]
LTF (Lactotransferrin)	- detected in epididymal tissue and sperm, seminal plasma - regulates the availability and catalytic activity of iron - possesses antibiotic properties in the reproductive system - binds lipopolysacharides, heparin, glycosaminoglycans, DNA, and ions - its addition to cryoprotective extender significantly improves the quality of frozen-thawed ram semen	[60,61,62]
NPC2 (NPC intracellular cholesterol transporter 2)	- detected in epididymis and ejaculated sperm - involves in cholesterol efflux from lysosomes - functions as intracellular cholesterol transporter - provides protection to sperm against cryo-induced cold shock damage - potential freezability marker of semen quality	[15,36,38,39,40,43]

## 5. Conclusions

Although the ES protein profiles differed between the study groups in protein presence and abundance, LTF was dominant in both groups. This study revealed that some proteins are present in higher amounts in canine ES, indicating good progressive motility. These were ACTB, ce10, LTF, NPC2 and CRISP2. Two of these proteins, ce10 and NPC2, differed significantly in content between the GSM and PSM groups, as confirmed by Western blot analysis. Analyses of the presence and content of individual proteins in canine ES in relation to the progressive motility value are part of the search for MRSPs, which, in the future, may be used to develop diagnostic tests related to assisted reproduction in dogs.

## Data Availability

The data underlying this article are available in the article and in its online Supplementary Materials.

## Supplementary Materials

The following supporting information can be downloaded at https://www.mdpi.com/article/10.3390/cells15010085/s1, Supplementary Table S1: Proteins of the cauda epididymal sperm (ES) of dogs (*Canis lupus familiaris*) with good sperm motility (GSM); Supplementary Table S2: Proteins of the cauda epididymal sperm (ES) of dogs (*Canis lupus familiaris*) with poor sperm motility (PSM); Supplementary Table S3: The set of canine epididymal sperm (ES) proteins that constitute <1% in the good sperm motility (GSM) group; Supplementary Table S4: The set of canine epididymal sperm (ES) proteins that constitute <1% in the poor sperm motility (PSM) group.

## Abbreviations

The following abbreviations are used in this manuscript:

ACTB	Actin cytoplasmic 1
ADAMTS5	ADAM metallopeptidase with thrombospondin type 1 motif 5
AFP	alpha fetoprotein
AGL	Glycogen debranching enzyme
AKAP4	A-Kinase anchoring protein 4
ALB/ALBs	Albumin/Albumins
ALH	Amplitude of lateral head displacement
ANKS1B	Ankyrin repeat and sterile alpha motif domain-containing protein 1B
ANO9	Anoctamin-9
APCbp*	APC-binding protein EB1
AREL1	Apoptosis-resistant E3 ubiquitin protein ligase 1
ASPM	Abnormal spindle-like microcephaly-associated protein homolog
BCF	Beat cross frequency
BP	Biological process (GO category)
BVES	Blood vessel epicardial substance
CASA	Computer-assisted semen analysis
CC	Cellular component (GO category)
ce10	CE10 protein (canine epididymal protein 10)
CBL	E3 ubiquitin-protein ligase CBL
CEMIP	Hyaluronoglucosaminidase
CLU	Clusterin
CPs	Common proteins
CRISP2	Cysteine-rich secretory protein 2
CNTD*	Cyclin N-terminal domain-containing protein
DPBS	Dulbecco’s phosphate-buffered saline
ES	Epididymal sperm
ENGASE	Endo-beta-N-acetylglucosaminidase
ESI	Electrospray ionization
FAT3	FAT atypical cadherin 3
GAPDH	Glyceraldehyde-3-phosphate dehydrogenase
GO	Gene Ontology
GPX5	Glutathione peroxidase 5
GSM	Good sperm motility group
HE	Human epididymal proteins
IQGAP3	IQ motif-containing GTPase-activating protein 3
LCNL1	Lipocln_cytosolic_FA-bd_dom domain-containing protein
LC–MS	Liquid chromatography–mass spectrometry
LIN	Linearity
LOC607874	Cystatin domain-containing protein
LTF	Lactotransferrin
MF	Molecular function (GO category)
MLLT1	Myeloid/lymphoid leukemia translocated to 1
MMP2	Metalloproteinase 2
MS/MS	Tandem mass spectrometry
MRSPs	Motility-related sperm proteins
NanoUPLC-Q-TOF/MS	Nano-ultra performance liquid chromatography–quadrupole time-of-flight mass spectrometry
NPC2	NPC intracellular cholesterol transporter 2
OPN	Osteopontin
PAK4	non-specific serine/threonine protein kinase
PC	Protein class (PANTHER)
PANTHER	Protein ANalysis THrough Evolutionary Relationships
PCA	Principal component analysis
PMOT	Progressive motility
PSM	Poor sperm motility group
PVDF	Polyvinylidene fluoride membranes
PTGDS	Prostaglandin-H2 D-isomerase
RIPA	Radioimmunoprecipitation assay buffer
RPL32	60S ribosomal protein L32
SACS	Sacsin molecular chaperone
SDS-PAGE	Sodium Dodecyl Sulfate–Polyacrylamide Gel Electrophoresis
SIPs	Sperm intracellular proteins
SMAPs	Sperm membrane-associated proteins
SMC1B	Structural maintenance of chromosomes protein
SPRED1	Sprouty-related EVH1 domain-containing protein 1
STR	Straightness
ST6GALNAC5	ST6 N-acetylgalactosaminide alpha-2,6-sialyltransferase 5
SWISSPROT	Swiss-Prot protein database
TAF1	Transcription initiation factor TFIID subunit
TMOT	Total motility
TET3	Methylcytosine dioxygenase TET3
TRPV1	Transient receptor potential cation channel subfamily V member 1
TTN	Titin
UPs	Unique proteins
VAP	Average path velocity
VCL	Curvilinear velocity
VSL	Straight-line velocity
*v*/*v*	Volume per volume
WAPdcp*	WAP domain-containing protein
ZNF654	Zinc finger protein 654
