# Supplementary material for: Potential Proteins Associated with Canine Epididymal Sperm Motility
Source: Cells. 2026 Jan 4;15(1):85. doi: 10.3390/cells15010085 (PMC12786130; doi:10.3390/cells15010085)
Supplement: Supplementary file 1 [file cells-15-00085-s001.zip › Supplementary Table S1.pdf]

Supplementary Table S1. Proteins of the cauda epididymal sperm (ES) of dogs (*Canis lupus familiaris*) with good sperm motility (GSM).

Description	|Log Prob|	Best |Log Prob|	Best score	Total Intensity	# of spectra	# of unique peptides	# of mod peptides	Coverage %	# AA's in protein	Protein DB number
>tr|F1PPP9|F1PPP9_CANLF Family with sequence similarity 135 member A OS=Canis lupus familiaris OX=9615 GN=FAM135A PE=3 SV=3	0.58	0.59	34.4	2655858.4	1	1	0	1.22	1399	6815
>tr|F1PFD6|F1PFD6_CANLF Teneurin transmembrane protein 4 OS=Canis lupus familiaris OX=9615 GN=TENM4 PE=3 SV=3	0.55	0.55	34.8	74097.8	1	1	0	0.32	2852	5565
>tr|A0A5F4D463|A0A5F4D463_CANLF Roundabout guidance receptor 1 OS=Canis lupus familiaris OX=9615 GN=ROBO1 PE=4 SV=1	0.22	0.22	37.7	89484.8	1	1	0	1.42	1691	5706
>tr|A0A5F4BZW4|A0A5F4BZW4_CANLF Malonyl-CoA decarboxylase OS=Canis lupus familiaris OX=9615 GN=MLYCD PE=4 SV=1	0.19	0.19	40.9	355115	1	1	1	5.42	461	4809
>tr|A0A5F4BU36|A0A5F4BU36_CANLF Titin OS=Canis lupus familiaris OX=9615 GN=TTN PE=3 SV=1	0.52	0.54	42.2	273602.7	1	1	0	0.08	27097	33785
>tr|A0A5F4DCW5|A0A5F4DCW5_CANLF Fibrosin like 1 OS=Canis lupus familiaris OX=9615 GN=FBRSL1 PE=4 SV=1	0.53	0.52	43.8	3992072.6	2	2	2	2.3	566	32781
>tr|A0A5F4CB90|A0A5F4CB90_CANLF Nuclear cap-binding protein subunit 2 OS=Canis lupus familiaris OX=9615 PE=3 SV=1	0.58	0.58	48.1	102484.5	1	1	0	4.52	155	44031
>tr|J9P7Y2|J9P7Y2_CANLF Angiotensin-converting enzyme OS=Canis lupus familiaris OX=9615 GN=ACE2 PE=3 SV=1	0.8	0.8	53.6	4424277.3	1	1	0	1.62	804	1041

>tr|E2QUV3|E2QUV3_CANLF Alpha-2-HS-glycoprotein OS=Canis lupus familiaris OX=9615 GN=AHSG PE=4 SV=2	0.7	0.66	59.9	14914548	3	1	0	5.48	365	20747
>sp|E2QY99|MPP5_CANLF MAGUK p55 subfamily member 5 OS=Canis lupus familiaris OX=9615 GN=MPP5 PE=1 SV=1	0.19	0.19	64.5	175268.7	1	1	1	2.22	675	246
>tr|A0A5F4CLN6|A0A5F4CLN6_CANLF DLG associated protein 4 OS=Canis lupus familiaris OX=9615 GN=DLGAP4 PE=3 SV=1	0.43	0.43	69.4	139918.9	1	1	0	3.04	461	7940
>tr|E2QXS1|E2QXS1_CANLF Lipase A, lysosomal acid type OS=Canis lupus familiaris OX=9615 GN=LIPA PE=4 SV=3	0.51	0.51	70.2	7158421.9	1	1	1	3.6	500	2185
>tr|A0A5F4DA89|A0A5F4DA89_CANLF Sorting nexin 25 OS=Canis lupus familiaris OX=9615 GN=SNX25 PE=3 SV=1	0.4	0.41	70.9	267500.8	1	1	1	1.37	950	8417
>tr|A0A5F4BQA2|A0A5F4BQA2_CANLF Dermatan sulfate epimerase OS=Canis lupus familiaris OX=9615 GN=DSE PE=3 SV=1	0.46	0.44	73.9	420173.8	2	1	0	2.6	308	8924
>tr|A0A5F4DG16|A0A5F4DG16_CANLF Myosin XVIIIA OS=Canis lupus familiaris OX=9615 GN=MYO18A PE=3 SV=1	0.41	0.41	74.3	162216.5	1	1	0	0.45	2426	21190
>tr|E2RHP1|E2RHP1_CANLF T cell activation inhibitor, mitochondrial OS=Canis lupus familiaris OX=9615 GN=TCAIM PE=4 SV=1	0.24	0.2	84.4	14970549.4	3	1	0	2.01	497	25418
>tr|A0A5F4CRP9|A0A5F4CRP9_CANLF Tumor suppressor candidate 3 OS=Canis lupus familiaris OX=9615 GN=TUSC3 PE=3 SV=1	0.42	0.3	84.9	38762611.3	7	1	0	3.18	314	6225
>tr|A0A5F4CDY8|A0A5F4CDY8_CANLF Kinesin family member 16B OS=Canis lupus familiaris OX=9615 GN=KIF16B PE=3 SV=1	0.95	0.02	85.8	85362610.9	61	1	1	0.96	1245	3757

>tr|E2R269|E2R269_CANLF Ubiquitinyl hydrolase 1 OS=Canis lupus familiaris OX=9615 GN=VCPIP1 PE=4 SV=1	0.99	0.95	87.7	8805102.3	3	1	0	1.39	1220	40849
>tr|F1PL17|F1PL17_CANLF TNF receptor superfamily member 13C OS=Canis lupus familiaris OX=9615 GN=TNFRSF13C PE=4 SV=3	0.55	0.55	93.7	4386567.6	1	1	0	3.15	286	13040
>tr|E2R6E0|E2R6E0_CANLF Lipocln_cytosolic_FA-bd_dom domain-containing protein OS=Canis lupus familiaris OX=9615 GN=LCNL1 PE=3 SV=2	2.36	1.74	95.8	17961992.8	3	2	0	7.69	299	1932
>tr|J9P6K3|J9P6K3_CANLF PHD finger protein 2 OS=Canis lupus familiaris OX=9615 GN=PHF2 PE=4 SV=1	0.2	0.16	97.7	265542.8	3	1	1	1.28	1096	24614
>tr|A0A5F4CBG5|A0A5F4CBG5_CANLF Ubiquitin specific peptidase 47 OS=Canis lupus familiaris OX=9615 GN=USP47 PE=4 SV=1	0.58	0.54	102.8	350029.6	3	1	0	1.62	1299	1284
>tr|J9NW72|J9NW72_CANLF Sperm associated antigen 8 OS=Canis lupus familiaris OX=9615 GN=SPAG8 PE=4 SV=1	0.79	0.69	103.3	28470734	6	1	0	4.07	442	10371
>tr|F1Q075|F1Q075_CANLF Nuclear receptor subfamily 1 group I member 2 OS=Canis lupus familiaris OX=9615 GN=NR1I2 PE=3 SV=3	0.28	0.26	109.7	10900461.8	2	1	1	2.12	471	44187
>tr|A0A5F4CCD0|A0A5F4CCD0_CANLF Cysteine rich secretory protein 2 OS=Canis lupus familiaris OX=9615 GN=CRISP2 PE=3 SV=1	0.64	0.64	109.9	117327.5	1	1	0	4.82	311	11017
>tr|E2RFA3|E2RFA3_CANLF RNA helicase OS=Canis lupus familiaris OX=9615 GN=DDX18 PE=3 SV=2	0.82	0.82	112.1	245821.5	1	1	0	3.44	669	32049
>tr|E2RHS5|E2RHS5_CANLF tRNA (guanine-N(7)-)-methyltransferase non-catalytic subunit WDR4 OS=Canis lupus familiaris OX=9615 GN=WDR4 PE=3 SV=1	0.5	0.36	112.1	11547415.2	8	1	0	1.72	406	22738

>sp|Q9XS65|PTGDS_CANLF Prostaglandin-H2 D-isomerase OS=Canis lupus familiaris OX=9615 GN=PTGDS PE=2 SV=1	0.24	0.18	119.4	31259064.7	5	2	1	10.47	191	165
>tr|E2QUM4|E2QUM4_CANLF Pseudopodium enriched atypical kinase 1 OS=Canis lupus familiaris OX=9615 GN=PEAK1 PE=4 SV=3	0.52	0.52	126.8	16893351.7	3	2	2	1.73	1674	5482
>tr|F1P9Y3|F1P9Y3_CANLF Complex I-30kD OS=Canis lupus familiaris OX=9615 GN=NDUFS3 PE=3 SV=2	0.4	0.4	135.2	328949.1	1	1	0	4.04	322	18622
>sp|F1PRN2|MYO1D_CANLF Unconventional myosin-Id OS=Canis lupus familiaris OX=9615 GN=MYO1D PE=1 SV=2	0.82	0.74	171.1	18091275.2	5	1	0	0.3	1006	763
>sp|B8K1W2|ABCBB_CANLF Bile salt export pump OS=Canis lupus familiaris OX=9615 GN=Abcb11e PE=1 SV=1	0.56	0.54	178.5	7250369.8	2	1	0	0.3	1325	527
>tr|F1PJY1|F1PJY1_CANLF Mannosyl-glycoprotein endo-beta-N-acetylglucosaminidase OS=Canis lupus familiaris OX=9615 GN=ENGASE PE=3 SV=3	0.25	0.02	183.3	58690567.6	13	2	2	1.74	690	32761
>tr|A0A5F4DGF5|A0A5F4DGF5_CANLF Alkaline phosphatase OS=Canis lupus familiaris OX=9615 GN=ALPL PE=3 SV=1	1.54	1.54	195	5468783.8	1	1	0	2.45	572	6357
>tr|F1PJ71|F1PJ71_CANLF Glutathione peroxidase OS=Canis lupus familiaris OX=9615 GN=GPX5 PE=3 SV=2	0.63	0.57	215.9	4480646.2	4	1	1	6.33	221	19009
>sp|O18840|ACTB_CANLF Actin, cytoplasmic 1 OS=Canis lupus familiaris OX=9615 GN=ACTB PE=2 SV=3	2.1	1.13	223.6	1148831.5	6	3	0	12	375	642
>tr|F1PR54|F1PR54_CANLF Lactotransferrin OS=Canis lupus familiaris OX=9615 GN=LTF PE=3 SV=1	5.24	1.63	235.1	127971923.9	27	8	0	12.71	708	40436
>tr|E2RCT1|E2RCT1_CANLF WAP domain-containing protein OS=Canis lupus familiaris OX=9615 PE=4 SV=2	3.82	2.59	249	28068068.5	5	2	0	14.66	116	21717

>tr|E2RG75|E2RG75_CANLF Inactive ribonuclease-like protein 9 OS=Canis lupus familiaris OX=9615 GN=RNASE9 PE=3 SV=2	2.11	2.05	300.5	9687786.3	4	1	1	4.55	198	41734
>tr|F2Z4Q6|F2Z4Q6_CANLF Alpha fetoprotein OS=Canis lupus familiaris OX=9615 GN=AFP PE=4 SV=2	20.91	4.01	308.9	651306681.1	130	17	2	33.28	637	24990
>tr|Q9XSV4|Q9XSV4_CANLF CE10 protein OS=Canis lupus familiaris OX=9615 GN=ce10 PE=2 SV=1	5.37	3.36	320.2	223705143.2	44	3	0	12.73	110	41542
>sp|P49822|ALBU_CANLF Albumin OS=Canis lupus familiaris OX=9615 GN=ALB PE=1 SV=3	13.42	2.87	336.3	18325290.9	58	12	1	23.85	608	490
>tr|A0A5F4CWK7|A0A5F4CWK7_CANLF Ig-like domain-containing protein OS=Canis lupus familiaris OX=9615 PE=4 SV=1	2.46	2.42	346.4	5731538.2	3	1	1	17	100	33283
>sp|Q28894|WFDC2_CANLF WAP four-disulfide core domain protein 2 OS=Canis lupus familiaris OX=9615 GN=WFDC2 PE=2 SV=1	3.06	2.68	393.1	96221301.8	20	1	0	6.45	124	53
>sp|Q28895|NPC2_CANLF NPC intracellular cholesterol transporter 2 OS=Canis lupus familiaris OX=9615 GN=NPC2 PE=2 SV=1	8.08	3.25	414	187319398.5	27	4	0	30.2	149	153
>tr|F1P7F0|F1P7F0_CANLF DENN domain containing 2A OS=Canis lupus familiaris OX=9615 GN=DENND2A PE=4 SV=3	0.1	0.01	34.3	80662709.3	7	1	0	0.45	1104	2294
>tr|A0A5F4CEY4|A0A5F4CEY4_CANLF ELL associated factor 2 OS=Canis lupus familiaris OX=9615 GN=EAF2 PE=3 SV=1	0.1	0.02	35.6	721015.9	1	1	0	0.74	543	6686
>tr|F1PCW0|F1PCW0_CANLF Golgin A4 OS=Canis lupus familiaris OX=9615 GN=GOLGA4 PE=4 SV=3	0.18	0.02	35.6	134233498.2	13	2	0	0.45	2239	2331
>tr|A0A5F4CL05|A0A5F4CL05_CANLF Chloride channel protein OS=Canis lupus familiaris OX=9615 GN=CLCN7 PE=3 SV=1	0.35	0.27	38.3	62752256	5	1	0	0.59	844	10399

>tr|A0A5F4BU36|A0A5F4BU36_CANLF Titin OS=Canis lupus familiaris OX=9615 GN=TTN PE=3 SV=1	0.23	0.03	38.3	175131771.3	15	2	0	0.04	27097	33785
>tr|J9JHT4|J9JHT4_CANLF Unc-79 homolog, NALCN channel complex subunit OS=Canis lupus familiaris OX=9615 GN=UNC79 PE=4 SV=2	0.36	0.27	38.3	74913627.1	6	1	0	0.19	2622	39915
>tr|A0A5F4CF26|A0A5F4CF26_CANLF S1 RNA binding domain 1 OS=Canis lupus familiaris OX=9615 GN=SRBD1 PE=4 SV=1	0.1	0	40	1975157.3	2	1	0	0.5	1003	6216
>tr|A0A5F4DHH0|A0A5F4DHH0_CANLF ATP binding cassette subfamily A member 1 OS=Canis lupus familiaris OX=9615 GN=ABCA1 PE=4 SV=1	0.1	0.03	41.2	12918943.9	1	1	0	0.23	2175	3709
>tr|F1PIS0|F1PIS0_CANLF Ryanodine receptor 1 OS=Canis lupus familiaris OX=9615 GN=RYR1 PE=4 SV=3	0.1	0.03	41.2	12918943.9	1	1	0	0.1	5038	8330
>tr|J9NT31|J9NT31_CANLF Thymocyte selection associated family member 2 OS=Canis lupus familiaris OX=9615 GN=THEMIS2 PE=3 SV=1	0.1	0.01	42	830275.7	1	1	0	0.78	642	9120
>tr|J9P9B1|J9P9B1_CANLF Mitochondrial translational initiation factor 2 OS=Canis lupus familiaris OX=9615 GN=MTIF2 PE=4 SV=2	0.2	0.02	43.1	132212898.4	12	1	0	0.75	668	1755
>tr|E2RIK1|E2RIK1_CANLF Phosphatidylinositol-4-phosphate 3-kinase OS=Canis lupus familiaris OX=9615 GN=PIK3C2G PE=3 SV=3	0.48	0.44	55.6	59105479.1	3	1	0	0.42	1445	5924
>tr|A0A5F4D3E3|A0A5F4D3E3_CANLF Poly [ADP-ribose] polymerase OS=Canis lupus familiaris OX=9615 GN=PARP2 PE=4 SV=1	0.1	0.03	56.3	4850691.2	1	1	0	2.16	602	5219
>tr|A0A5F4BZ61|A0A5F4BZ61_CANLF G_PROTEIN_RECEP_F1_2 domain-containing protein OS=Canis lupus familiaris OX=9615 GN=OR5D13 PE=4 SV=1	0.1	0	57.1	18121586.8	2	1	0	1.7	294	37537

>tr|E2RKA1|E2RKA1_CANLF Tyrosine-protein kinase receptor OS=Canis lupus familiaris OX=9615 GN=NTRK2 PE=3 SV=2	0.2	0.18	58	54282136.4	2	1	0	0.73	822	20603
>tr|A0A5F4CR89|A0A5F4CR89_CANLF Voltage-dependent R-type calcium channel subunit alpha OS=Canis lupus familiaris OX=9615 GN=CACNA1E PE=3 SV=1	0.1	0.01	58.2	195241904.1	11	1	0	0.19	2688	1145
>tr|J9PAQ2|J9PAQ2_CANLF Cyclin N-terminal domain-containing protein OS=Canis lupus familiaris OX=9615 PE=3 SV=1	0.1	0.01	58.2	195241904.1	11	1	0	1.25	400	2385
>tr|J9P3H8|J9P3H8_CANLF ATM interactor OS=Canis lupus familiaris OX=9615 GN=ATMIN PE=4 SV=2	0.1	0.02	58.3	8764056.9	9	1	0	0.58	863	882
>sp|Q9XSU7|RL27_CANLF 60S ribosomal protein L27 OS=Canis lupus familiaris OX=9615 GN=RPL27 PE=2 SV=3	0.62	0.02	60.2	117000186.3	78	1	0	3.68	136	314
>tr|E2RHV3|E2RHV3_CANLF Tripartite motif containing 23 OS=Canis lupus familiaris OX=9615 GN=TRIM23 PE=4 SV=3	0.1	0	61.9	1484135.6	1	1	0	0.87	574	1124
>tr|E2R5H9|E2R5H9_CANLF Blood vessel epicardial substance OS=Canis lupus familiaris OX=9615 GN=BVES PE=3 SV=1	0.4	0.13	63.2	353287637.6	15	1	0	1.67	360	8594
>tr|E2QRT5|E2QRT5_CANLF Structural maintenance of chromosomes protein OS=Canis lupus familiaris OX=9615 GN=SMC1B PE=3 SV=1	0.86	0.44	64.4	496319666.9	22	1	0	0.49	1235	2380
>tr|A0A5F4CM35|A0A5F4CM35_CANLF Glycerophosphodiester phosphodiesterase domain containing 2 OS=Canis lupus familiaris OX=9615 GN=GDPD2 PE=3 SV=1	0.1	0.01	64.7	41155352.5	4	1	1	0.65	614	16708

>tr|J9P5T2|J9P5T2_CANLF Non-specific serine/threonine protein kinase OS=Canis lupus familiaris OX=9615 GN=WNK3 PE=4 SV=2	0.1	0	65.7	7615324	6	1	0	0.22	2294	5229
>tr|J9NSK0|J9NSK0_CANLF Basic helix-loop-helix family member b9 OS=Canis lupus familiaris OX=9615 GN=BHLHB9 PE=3 SV=1	0.39	0.37	67.3	12627010.9	2	1	0	1.09	550	35937
>tr|F1PPN1|F1PPN1_CANLF Scaffold attachment factor B2 OS=Canis lupus familiaris OX=9615 GN=SAFB2 PE=4 SV=3	0.1	0	69.7	113741557.9	6	1	0	0.63	954	22763
>tr|J9NSS6|J9NSS6_CANLF DNA helicase OS=Canis lupus familiaris OX=9615 GN=CHD2 PE=4 SV=2	0.1	0.08	70	4569948.7	6	1	0	0.28	1780	1264
>sp|Q863Z4|MTPN_CANLF Myotrophin OS=Canis lupus familiaris OX=9615 GN=MTPN PE=3 SV=3	0.43	0.43	70.8	1791867	2	1	0	4.24	118	229
>tr|A0A5F4BSC2|A0A5F4BSC2_CANLF Galectin OS=Canis lupus familiaris OX=9615 GN=LGALS4 PE=4 SV=1	0.1	0	74.2	55878627.6	6	1	0	1.62	309	19997
>tr|F1PBJ1|F1PBJ1_CANLF Methylcytosine dioxygenase TET OS=Canis lupus familiaris OX=9615 GN=TET3 PE=3 SV=2	0.1	0	74.2	15265630.4	10	1	0	0.28	1795	1529
>tr|F1PGK9|F1PGK9_CANLF ADAM metallopeptidase with thrombospondin type 1 motif 5 OS=Canis lupus familiaris OX=9615 GN=ADAMTS5 PE=4 SV=3	0.12	0.02	75.8	41480860.2	27	1	0	0.59	845	11956
>tr|A0A5F4DKM7|A0A5F4DKM7_CANLF Terminal uridylyl transferase 4 OS=Canis lupus familiaris OX=9615 GN=TUT4 PE=4 SV=1	0.1	0.05	77.6	16269702.7	3	1	0	0.19	1611	941
>tr|A0A5F4CMY6|A0A5F4CMY6_CANLF Rho-associated protein kinase 2 OS=Canis lupus familiaris OX=9615 GN=ROCK2 PE=3 SV=1	0.1	0	82.4	1903807.5	2	1	0	0.35	1145	1527

>tr|A0A5F4CCF5|A0A5F4CCF5_CANLF Interleukin 6 signal transducer OS=Canis lupus familiaris OX=9615 GN=IL6ST PE=4 SV=1	0.1	0.02	83.6	12292416.5	2	1	1	34.85	66	3968
>tr|A0A5F4C7Q7|A0A5F4C7Q7_CANLF IQ motif and Sec7 domain ArfGEF 1 OS=Canis lupus familiaris OX=9615 GN=IQSEC1 PE=3 SV=1	0.17	0.02	83.8	101797261.8	10	1	0	0.51	971	4269
>sp|A2IBY8|MIP_CANLF Lens fiber major intrinsic protein OS=Canis lupus familiaris OX=9615 GN=MIP PE=2 SV=1	0.12	0.08	85.3	28513209.4	13	1	0	1.9	263	112
>tr|A0A5F4CPU3|A0A5F4CPU3_CANLF SEC24 homolog D, COPII coat complex component OS=Canis lupus familiaris OX=9615 GN=SEC24D PE=3 SV=1	0.15	0.02	89.3	23044839.7	24	1	0	0.53	946	2125
>tr|A0A5F4CQE4|A0A5F4CQE4_CANLF LARGE xylosyl- and glucuronyltransferase 1 OS=Canis lupus familiaris OX=9615 GN=LARGE1 PE=4 SV=1	0.1	0	91.9	119276057	15	1	0	0.7	714	7538
>tr|F1Q0P9|F1Q0P9_CANLF AT-hook containing transcription factor 1 OS=Canis lupus familiaris OX=9615 GN=AHCTF1 PE=4 SV=3	0.23	0.03	93.2	81588408.8	13	2	0	0.26	2321	1521
>tr|A0A5F4C0S7|A0A5F4C0S7_CANLF HEAT repeat containing 5A OS=Canis lupus familiaris OX=9615 GN=HEATR5A PE=3 SV=1	0.1	0.02	93.8	20628990.4	15	1	0	0.25	1995	1753
>tr|E2RE16|E2RE16_CANLF Non-specific serine/threonine protein kinase OS=Canis lupus familiaris OX=9615 GN=PAK4 PE=4 SV=1	0.34	0.08	95.3	52479900.2	41	1	0	0.84	592	12735
>tr|A0A5F4CT13|A0A5F4CT13_CANLF Anion exchange protein OS=Canis lupus familiaris OX=9615 GN=SLC4A2 PE=3 SV=1	0.1	0	97.3	4850691.2	1	1	0	1.5	1263	4624
>tr|A0A5F4CUD4|A0A5F4CUD4_CANLF Transcription initiation factor TFIID subunit OS=Canis lupus familiaris OX=9615 GN=TAF1 PE=3 SV=1	0.61	0.12	101.2	379105437.2	33	2	0	0.53	1897	4265

>sp|Q2PQH8|GDE_CANLF Glycogen debranching enzyme OS=Canis lupus familiaris OX=9615 GN=AGL PE=2 SV=1	0.42	0.09	104.8	92985336	15	2	0	0.26	1533	23
>tr|A0A5F4DJV1|A0A5F4DJV1_CANLF ATP synthase subunit beta OS=Canis lupus familiaris OX=9615 GN=ATP5F1B PE=3 SV=1	0.1	0	106.5	5126973	1	1	0	2.42	619	6667
>tr|E2R186|E2R186_CANLF Fibroblast growth factor receptor OS=Canis lupus familiaris OX=9615 GN=FGFR1 PE=3 SV=3	0.1	0	116.7	43892865.4	8	1	0	0.59	853	9797
>tr|F1PLW8|F1PLW8_CANLF CUB and Sushi multiple domains 3 OS=Canis lupus familiaris OX=9615 GN=CSMD3 PE=4 SV=2	0.1	0	118.5	5474559.1	1	1	1	0.24	3707	17762
>tr|A0A5F4CW57|A0A5F4CW57_CANLF Alsin Rho guanine nucleotide exchange factor ALS2 OS=Canis lupus familiaris OX=9615 GN=ALS2 PE=4 SV=1	0.1	0.02	125	4998044.8	1	1	0	1.31	1523	1985
>tr|A0A5F4BXA7|A0A5F4BXA7_CANLF Leucine rich repeat containing 7 OS=Canis lupus familiaris OX=9615 GN=LRRC7 PE=3 SV=1	0.13	0.05	130.2	41644681.3	5	1	0	0.39	1542	2603
>tr|Q2A652|Q2A652_CANLF G-protein coupled receptor OS=Canis lupus familiaris OX=9615 GN=PTGDR2 PE=2 SV=1	0.1	0	131.2	22483401.3	3	1	0	1.25	400	41469
>tr|J9P434|J9P434_CANLF Myotubularin related protein 14 OS=Canis lupus familiaris OX=9615 GN=MTMR14 PE=4 SV=2	0.1	0	140.5	4695683.5	1	1	0	1.51	596	17413
>tr|F1P8J6|F1P8J6_CANLF RNA helicase OS=Canis lupus familiaris OX=9615 GN=DDX55 PE=3 SV=3	0.1	0	141.7	5985029.3	1	1	0	1.58	568	8934
>tr|A0A5F4DL64|A0A5F4DL64_CANLF Tyrosine-protein kinase receptor OS=Canis lupus familiaris OX=9615 GN=ROS1 PE=3 SV=1	0.1	0	143.3	3787080.6	1	1	0	0.22	2272	14391

>tr|F6PKZ1|F6PKZ1_CANLF Arylsulfatase A OS=Canis lupus familiaris OX=9615 GN=ARSA PE=3 SV=2	0.1	0	144.6	5287670.8	1	1	0	2.26	487	20206
>tr|A0A5F4C2X8|A0A5F4C2X8_CANLF Fibrosin like 1 OS=Canis lupus familiaris OX=9615 GN=FBRSL1 PE=4 SV=1	0.1	0	149.5	10397764.3	2	1	0	0.71	985	1104
>tr|A0A5F4D020|A0A5F4D020_CANLF VWFA domain-containing protein OS=Canis lupus familiaris OX=9615 PE=3 SV=1	0.1	0	150.6	12192369	2	1	0	1	798	27451
>tr|E2RSI6|E2RSI6_CANLF Ezrin OS=Canis lupus familiaris OX=9615 GN=EZR PE=4 SV=1	0.1	0	152.8	5439995	1	1	0	1.54	586	15650
>tr|E2RRP3|E2RRP3_CANLF LIM homeobox 5 OS=Canis lupus familiaris OX=9615 GN=LHX5 PE=4 SV=1	0.1	0	154.4	6313101.1	1	1	0	2.24	402	19607
>tr|E2R8N9|E2R8N9_CANLF WD repeat domain 87 OS=Canis lupus familiaris OX=9615 GN=WDR87 PE=4 SV=3	0.1	0.06	155.6	5147049.2	1	1	0	0.32	2806	14637
>tr|E2RN16|E2RN16_CANLF Mitogen-activated protein kinase kinase kinase 2 OS=Canis lupus familiaris OX=9615 GN=MAP3K2 PE=4 SV=2	0.1	0.01	157.8	8039775.1	1	1	0	0.97	620	34325
>tr|F1PZ46|F1PZ46_CANLF Tudor domain containing 12 OS=Canis lupus familiaris OX=9615 GN=TDRD12 PE=4 SV=3	0.1	0	159.6	11262139.3	2	1	0	0.23	1320	3323
>tr|A0A5F4CQH1|A0A5F4CQH1_CANLF Na(+)/H(+) exchange regulatory cofactor NHE-RF OS=Canis lupus familiaris OX=9615 GN=SLC9A3R1 PE=4 SV=1	0.1	0.01	163.6	4945695.2	1	1	0	2.23	359	1876
>tr|A0A5F4CQ96|A0A5F4CQ96_CANLF ATP binding cassette subfamily G member 8 OS=Canis lupus familiaris OX=9615 GN=ABCG8 PE=3 SV=1	0.1	0.03	163.8	5491250.2	1	1	0	1.84	707	17201

>tr|A0A5F4BT89|A0A5F4BT89_CANLF Olfactory receptor OS=Canis lupus familiaris OX=9615 GN=OR5W6 PE=3 SV=1	0.1	0	163.9	5372311.7	1	1	1	6.95	302	29923
>tr|E2R6E0|E2R6E0_CANLF Lipocln_cytosolic_FA-bd_dom domain-containing protein OS=Canis lupus familiaris OX=9615 GN=LCNL1 PE=3 SV=2	0.39	0.37	164.5	11428515.8	2	1	0	3.01	299	1932
>tr|A0A5F4DI20|A0A5F4DI20_CANLF Aryl hydrocarbon receptor nuclear translocator OS=Canis lupus familiaris OX=9615 GN=ARNT PE=4 SV=1	0.1	0.03	169.3	6591554.4	1	1	0	0.4	753	1214
>tr|A0A5F4D9L3|A0A5F4D9L3_CANLF Plexin C1 OS=Canis lupus familiaris OX=9615 GN=PLXNC1 PE=3 SV=1	0.1	0	172.8	5907173.8	1	1	0	0.19	1580	1260
>sp|Q8HYV8|ASB17_CANLF Ankyrin repeat and SOCS box protein 17 OS=Canis lupus familiaris OX=9615 GN=ASB17 PE=2 SV=1	0.1	0	172.9	21245230.7	4	1	0	1.02	295	404
>tr|A0A5F4C1D8|A0A5F4C1D8_CANLF Acrosin OS=Canis lupus familiaris OX=9615 GN=ACR PE=3 SV=1	0.44	0.44	175.1	2328870.8	2	1	0	2.84	423	43346
>tr|A0A5K1V0D8|A0A5K1V0D8_CANLF Sulfatase 2 OS=Canis lupus familiaris OX=9615 GN=SULF2 PE=3 SV=1	0.1	0	180	21470834.1	4	1	0	0.35	859	1192
>tr|A0A5F4CY61|A0A5F4CY61_CANLF 2-phospho-D-glycerate hydro-lyase OS=Canis lupus familiaris OX=9615 PE=3 SV=1	0.1	0.04	181.9	5887641.8	1	1	0	6.5	123	9061
>sp|P49822|ALBU_CANLF Albumin OS=Canis lupus familiaris OX=9615 GN=ALB PE=1 SV=3	0.35	0.35	182.5	5910507.1	1	1	0	1.64	608	490
>tr|A0A5F4CDD9|A0A5F4CDD9_CANLF Proline dehydrogenase OS=Canis lupus familiaris OX=9615 GN=PRODH2 PE=3 SV=1	0.1	0.01	183.8	4763870.7	1	1	1	1.69	415	14972

>sp|Q9TU53|CUBN_CANLF Cubilin OS=Canis lupus familiaris OX=9615 GN=CUBN PE=1 SV=1	0.1	0.05	188.7	17410876.3	3	1	0	0.08	3620	406
>tr|A0A5F4DIG1|A0A5F4DIG1_CANLF Interleukin 4 induced 1 OS=Canis lupus familiaris OX=9615 GN=IL4I1 PE=4 SV=1	0.53	0.45	189.4	30842373.8	5	3	0	5.08	571	21105
>tr|F1PTL9|F1PTL9_CANLF ATP binding cassette subfamily C member 8 OS=Canis lupus familiaris OX=9615 GN=ABCC8 PE=3 SV=3	0.1	0.02	191.9	4114660.3	1	1	0	0.28	1454	4249
>tr|F1P6B8|F1P6B8_CANLF Intraflagellar transport protein 57 homolog OS=Canis lupus familiaris OX=9615 GN=IFT57 PE=3 SV=3	0.1	0.03	197.6	17239961	3	1	0	0.62	482	4757
>tr|F1Q455|F1Q455_CANLF PKHD1 like 1 OS=Canis lupus familiaris OX=9615 GN=PKHD1L1 PE=4 SV=3	0.1	0.05	198.3	11758293.5	2	1	0	0.07	4263	3461
>tr|F1PKW7|F1PKW7_CANLF Tyrosine 3-monooxygenase/tryptophan 5-monooxygenase activation protein beta OS=Canis lupus familiaris OX=9615 GN=YWHAB PE=3 SV=3	0.1	0.02	205.2	4326583.6	1	1	0	2.03	246	2992
>tr|A0A5F4CZ62|A0A5F4CZ62_CANLF Dynein cytoplasmic 1 heavy chain 1 OS=Canis lupus familiaris OX=9615 GN=DYNC1H1 PE=3 SV=1	0.1	0.03	206.6	5836740.3	1	1	0	0.07	4329	3271
>tr|A0A5F4CPY7|A0A5F4CPY7_CANLF von Willebrand factor A domain containing 5A OS=Canis lupus familiaris OX=9615 GN=VWA5A PE=4 SV=1	0.45	0.45	208.9	7332917.7	1	1	0	0.75	803	1309
>tr|F1PD69|F1PD69_CANLF RING-type E3 ubiquitin transferase OS=Canis lupus familiaris OX=9615 GN=ZNRF3 PE=3 SV=2	0.1	0.03	214.5	5227219.4	1	1	0	1.21	827	33223
>sp|Q5TJE1|DAXX_CANLF Death domain-associated protein 6 OS=Canis lupus familiaris OX=9615 GN=DAXX PE=3 SV=1	0.95	0.91	215.2	16793805.3	3	1	0	0.54	737	429

>tr|E2RT65|E2RT65_CANLF Phosphoglycerate mutase OS=Canis lupus familiaris OX=9615 GN=PI4K2A PE=3 SV=2	0.56	0.52	216.8	14213607.7	3	1	0	4.33	254	15155
>sp|Q9TUX8|NOS3_CANLF Nitric oxide synthase, endothelial OS=Canis lupus familiaris OX=9615 GN=NOS3 PE=2 SV=1	0.1	0.03	216.9	5952588.6	1	1	0	0.25	1205	117
>tr|A0A5F4D9S5|A0A5F4D9S5_CANLF Hyaluronoglucosaminidase OS=Canis lupus familiaris OX=9615 GN=CEMIP PE=3 SV=1	0.48	0.46	220.7	13297319.1	2	1	0	0.24	1684	9775
>tr|E2RPK8|E2RPK8_CANLF Phosphatidylethanolamine binding protein 4 OS=Canis lupus familiaris OX=9615 GN=PEBP4 PE=3 SV=2	0.3	0.3	223.5	6067453.6	1	1	0	2.02	247	4725
>tr|F1PB79|F1PB79_CANLF Abhydrolase domain containing 12B OS=Canis lupus familiaris OX=9615 GN=ABHD12B PE=4 SV=3	0.1	0.02	224.1	5354930.4	1	1	0	2.87	349	30573
>tr|F6V7I1|F6V7I1_CANLF Dynein axonemal heavy chain 2 OS=Canis lupus familiaris OX=9615 GN=DNAH2 PE=3 SV=2	0.41	0.41	230.9	7039118.5	1	1	0	0.14	4378	7034
>tr|A0A5F4DIQ5|A0A5F4DIQ5_CANLF Helicase with zinc finger 2 OS=Canis lupus familiaris OX=9615 GN=HELZ2 PE=4 SV=1	0.1	0.03	231.4	11625071.6	2	1	0	0.14	2929	1253
>tr|F1P704|F1P704_CANLF Zona pellucida binding protein OS=Canis lupus familiaris OX=9615 GN=ZPBP PE=3 SV=3	0.57	0.48	252.7	25536808.4	4	2	0	7.73	362	12422
>sp|Q6AW47|EST5A_CANLF Carboxylesterase 5A OS=Canis lupus familiaris OX=9615 GN=CES5A PE=2 SV=1	3.04	1.47	266.7	66636742.9	11	4	0	5.22	575	629
>tr|A0A5F4BVF3|A0A5F4BVF3_CANLF Lactotransferrin OS=Canis lupus familiaris OX=9615 GN=LTF PE=3 SV=1	1.05	0.97	275.1	81961926.1	13	3	0	3.99	626	32850

>tr|J9P9J4|J9P9J4_CANLF Aldehyde dehydrogenase 1 family member A1 OS=Canis lupus familiaris OX=9615 GN=ALDH1A1 PE=3 SV=1	0.36	0.36	275.3	1566400.8	1	1	1	2.07	484	13765
>sp|O18840|ACTB_CANLF Actin, cytoplasmic 1 OS=Canis lupus familiaris OX=9615 GN=ACTB PE=2 SV=3	1.44	1.3	287.5	55329329.6	10	2	0	5.07	375	642
>tr|F1PJ71|F1PJ71_CANLF Glutathione peroxidase OS=Canis lupus familiaris OX=9615 GN=GPX5 PE=3 SV=2	2.53	2	304.6	106948021.5	16	4	0	25.34	221	19009
>tr|Q9XSV4|Q9XSV4_CANLF CE10 protein OS=Canis lupus familiaris OX=9615 GN=ce10 PE=2 SV=1	2.93	1.9	312.3	22146957.7	9	2	0	9.09	110	41542
>tr|A0A5F4C4P0|A0A5F4C4P0_CANLF Zinc finger FYVE domain-containing protein 26 OS=Canis lupus familiaris OX=9615 GN=ZFYVE26 PE=4 SV=1	0.24	0.24	37.2	88482.3	1	1	0	0.43	2339	3561
>tr|E2RIV7|E2RIV7_CANLF Syntrophin alpha 1 OS=Canis lupus familiaris OX=9615 GN=SNTA1 PE=3 SV=3	0.1	0.02	41.3	4832783.8	13	1	1	1.03	486	34454
>tr|E2RRM8|E2RRM8_CANLF Fer-1 like family member 6 OS=Canis lupus familiaris OX=9615 GN=FER1L6 PE=4 SV=3	0.14	0.14	42	631823	1	1	1	0.86	1868	6562
>tr|J9NSS6|J9NSS6_CANLF DNA helicase OS=Canis lupus familiaris OX=9615 GN=CHD2 PE=4 SV=2	0.1	0	46	13315861	30	1	0	0.28	1780	1264
>tr|E2R0D7|E2R0D7_CANLF Collagen type VI alpha 6 chain OS=Canis lupus familiaris OX=9615 GN=COL6A6 PE=4 SV=2	0.23	0.24	46.8	122484.2	1	1	0	0.62	2268	14682
>tr|A0A5F4DDF1|A0A5F4DDF1_CANLF DDB1- and CUL4-associated factor 10 OS=Canis lupus familiaris OX=9615 GN=DCAF10 PE=3 SV=1	0.88	0.88	49.9	122484.2	1	1	0	2.66	526	2777

>sp|Q9XSU7|RL27_CANLF 60S ribosomal protein L27 OS=Canis lupus familiaris OX=9615 GN=RPL27 PE=2 SV=3	0.1	0	52.7	16415776.7	38	1	0	3.68	136	314
>tr|E2RE16|E2RE16_CANLF Non-specific serine/threonine protein kinase OS=Canis lupus familiaris OX=9615 GN=PAK4 PE=4 SV=1	0.1	0	57	14718612.4	34	1	0	0.84	592	12735
>tr|F1P721|F1P721_CANLF Kinase suppressor of ras 2 OS=Canis lupus familiaris OX=9615 GN=KSR2 PE=4 SV=3	0.14	0.12	58	5298983.1	2	1	0	0.53	950	2276
>tr|F1PRU0|F1PRU0_CANLF WD_REPEATS_REGION domain-containing protein OS=Canis lupus familiaris OX=9615 GN=TLE7 PE=3 SV=2	0.14	0.12	58	5298983.1	2	1	0	1.16	431	22849
>tr|F1PA94|F1PA94_CANLF FRY like transcription coactivator OS=Canis lupus familiaris OX=9615 GN=FRYL PE=4 SV=3	1.05	1.05	61.2	97525.8	1	1	0	0.3	3014	11447
>tr|J9P432|J9P432_CANLF Glutamine--fructose-6-phosphate transaminase (isomerizing) OS=Canis lupus familiaris OX=9615 GN=GFPT1 PE=4 SV=2	0.71	0.71	67.8	2882998.9	1	1	0	1.18	677	7191
>tr|F1Q3S8|F1Q3S8_CANLF Transmembrane protease serine OS=Canis lupus familiaris OX=9615 GN=TMPRSS11A PE=3 SV=3	0.27	0.27	79.6	710362.8	1	1	1	2.12	424	1890
>sp|Q8WN22|PRKDC_CANLF DNA-dependent protein kinase catalytic subunit OS=Canis lupus familiaris OX=9615 GN=PRKDC PE=2 SV=1	1.07	0.72	83.4	70510292.8	9	3	0	0.19	4144	338
>tr|F1PH76|F1PH76_CANLF Olfactory receptor OS=Canis lupus familiaris OX=9615 GN=OR4H12 PE=3 SV=2	0.12	0.12	94.6	7559293.4	1	1	0	2.91	309	20031
>sp|P49822|ALBU_CANLF Albumin OS=Canis lupus familiaris OX=9615 GN=ALB PE=1 SV=3	1.27	0.75	97.6	37265884.2	6	2	0	4.77	608	490

>tr|F1P6B8|F1P6B8_CANLF Intraflagellar transport protein 57 homolog OS=Canis lupus familiaris OX=9615 GN=IFT57 PE=3 SV=3	0.15	0.15	103.7	6262225.7	1	1	0	0.83	482	4757
>sp|Q2PQH8|GDE_CANLF Glycogen debranching enzyme OS=Canis lupus familiaris OX=9615 GN=AGL PE=2 SV=1	0.98	0.39	106.2	60463153.8	13	5	0	0.65	1533	23
>tr|A0A5F4C7P9|A0A5F4C7P9_CANLF Beta-2-microglobulin OS=Canis lupus familiaris OX=9615 GN=B2M PE=4 SV=1	0.4	0.37	107.2	17663412.7	3	1	0	12.15	107	10611
>tr|J9P2T7|J9P2T7_CANLF 26S proteasome non-ATPase regulatory subunit 5 OS=Canis lupus familiaris OX=9615 PE=4 SV=1	0.21	0.19	111.7	10219823.1	2	1	0	1.95	461	23870
>tr|J9P3D0|J9P3D0_CANLF Solute carrier family 4 member 9 OS=Canis lupus familiaris OX=9615 GN=SLC4A9 PE=3 SV=2	0.36	0.35	114.2	10719768.4	2	1	0	1.12	893	31921
>tr|F1PBU5|F1PBU5_CANLF Non-specific serine/threonine protein kinase OS=Canis lupus familiaris OX=9615 GN=SMG1 PE=3 SV=3	0.63	0.63	114.8	5257953.9	1	1	0	0.08	3634	6898
>tr|A0A5F4DCW2|A0A5F4DCW2_CANLF F-box and leucine rich repeat protein 18 OS=Canis lupus familiaris OX=9615 GN=FBXL18 PE=4 SV=1	0.24	0.24	122.5	483286.5	1	1	1	1.46	756	5739
>tr|F1PHA9|F1PHA9_CANLF Motile sperm domain containing 2 OS=Canis lupus familiaris OX=9615 GN=MOSPD2 PE=4 SV=3	0.13	0.14	123.6	6466169.2	1	1	0	0.97	518	10201
>tr|E2R6E0|E2R6E0_CANLF Lipocln_cytosolic_FA-bd_dom domain-containing protein OS=Canis lupus familiaris OX=9615 GN=LCNL1 PE=3 SV=2	1.25	1.19	126.5	21536887.8	4	1	0	3.01	299	1932
>tr|J9P199|J9P199_CANLF tRNA (guanine(37)-N1)-methyltransferase OS=Canis lupus familiaris OX=9615 GN=TRMT5 PE=3 SV=2	0.16	0.14	130.3	14347364.2	2	1	0	2.02	495	29429

>tr|A0A5F4D9S5|A0A5F4D9S5_CANLF Hyaluronoglucosaminidase OS=Canis lupus familiaris OX=9615 GN=CEMIP PE=3 SV=1	0.35	0.21	133.4	56062704.2	8	1	0	0.24	1684	9775
>tr|A0A5F4CEM0|A0A5F4CEM0_CANLF Proline rich coiled-coil 2B OS=Canis lupus familiaris OX=9615 GN=PRRC2B PE=4 SV=1	0.1	0.02	142.5	2893515.9	7	1	1	0.58	2235	37089
>tr|E2RBL7|E2RBL7_CANLF DNA polymerase OS=Canis lupus familiaris OX=9615 GN=POLL PE=3 SV=2	0.32	0.3	143.6	10826999.5	2	1	0	1.6	625	22831
>tr|E2R1V3|E2R1V3_CANLF U2 snRNP associated SURP domain containing OS=Canis lupus familiaris OX=9615 GN=U2SURP PE=4 SV=3	0.12	0.12	146	5019941.6	1	1	0	0.78	1029	12656
>tr|A0A5F4DI92|A0A5F4DI92_CANLF DNA helicase OS=Canis lupus familiaris OX=9615 GN=CHD6 PE=3 SV=1	0.16	0.14	152.6	9635686.3	2	1	0	0.26	2685	3470
>tr|E2RDS7|E2RDS7_CANLF Zinc finger protein 398 OS=Canis lupus familiaris OX=9615 GN=ZNF398 PE=4 SV=2	0.16	0.12	157.4	17209915.4	3	1	0	1.4	642	44996
>tr|F1Q2M4|F1Q2M4_CANLF DNA topoisomerase 2 OS=Canis lupus familiaris OX=9615 GN=TOP2A PE=3 SV=3	0.35	0.32	158.8	9534871.7	2	2	0	0.52	1532	1808
>sp|F1PRN2|MYO1D_CANLF Unconventional myosin-Id OS=Canis lupus familiaris OX=9615 GN=MYO1D PE=1 SV=2	1.09	0.57	160.8	52902342.3	9	2	0	0.6	1006	763
>tr|J9JHJ0|J9JHJ0_CANLF Testis expressed 50 OS=Canis lupus familiaris OX=9615 GN=TEX50 PE=4 SV=2	0.12	0.1	162.5	9541580.5	2	1	1	7.34	177	3785
>tr|A0A5F4DGX9|A0A5F4DGX9_CANLF Target of myb1 membrane trafficking protein OS=Canis lupus familiaris OX=9615 GN=TOM1 PE=3 SV=1	0.16	0.08	164.8	39625191	5	1	0	3.35	477	40074

>sp|Q28279|CNGA1_CANLF cGMP-gated cation channel alpha-1 OS=Canis lupus familiaris OX=9615 GN=CNGA1 PE=2 SV=1	0.36	0.34	164.9	4503656.9	2	1	0	0.58	691	455
>tr|A0A5F4DCA4|A0A5F4DCA4_CANLF Reverse transcriptase domain-containing protein OS=Canis lupus familiaris OX=9615 PE=4 SV=1	0.54	0.48	167.2	22970793.4	4	1	0	0.31	978	860
>tr|A0A5F4DKA1|A0A5F4DKA1_CANLF Zinc finger protein 335 OS=Canis lupus familiaris OX=9615 GN=ZNF335 PE=4 SV=1	0.14	0.12	172.8	14857466.3	3	2	0	1.09	1380	32203
>tr|A0A5F4DIZ5|A0A5F4DIZ5_CANLF Kinesin-like protein OS=Canis lupus familiaris OX=9615 GN=KIF9 PE=3 SV=1	0.14	0.06	174.8	47784525.6	5	1	0	1.5	732	7709
>tr|F1PTB2|F1PTB2_CANLF Interleukin 6 signal transducer OS=Canis lupus familiaris OX=9615 GN=IL6ST PE=3 SV=3	0.44	0.42	174.9	16803218.6	2	1	0	0.35	867	4860
>tr|A0A5F4CT98|A0A5F4CT98_CANLF [Heparan sulfate]-glucosamine N-sulfotransferase OS=Canis lupus familiaris OX=9615 GN=NDST1 PE=3 SV=1	0.14	0.14	177.6	34027788.6	5	2	1	2.04	932	9786
>tr|J9P2Y5|J9P2Y5_CANLF MAK16 homolog OS=Canis lupus familiaris OX=9615 GN=MAK16 PE=3 SV=2	0.55	0.55	189.8	7568316.9	2	2	1	1.34	599	17345
>tr|E2R531|E2R531_CANLF Zinc finger protein 462 OS=Canis lupus familiaris OX=9615 GN=ZNF462 PE=4 SV=3	0.57	0.57	198.6	25529808.8	6	2	1	0.58	2398	22062
>tr|J9NTK2|J9NTK2_CANLF J domain-containing protein OS=Canis lupus familiaris OX=9615 GN=DNAJC12 PE=4 SV=2	0.27	0.03	202	68153747.8	13	1	0	4.72	106	2310
>tr|A0A5F4BQW4|A0A5F4BQW4_CANLF Zinc finger protein 654 OS=Canis lupus familiaris OX=9615 GN=ZNF654 PE=4 SV=1	0.11	0.1	203.5	23135482.6	4	1	1	1.03	1170	6910

>tr|F1PEX6|F1PEX6_CANLF Tyrosine-protein kinase OS=Canis lupus familiaris OX=9615 GN=ABL1 PE=3 SV=2	0.17	0.02	219.4	74134182.7	11	1	1	0.7	1150	1051
>tr|E2RCT1|E2RCT1_CANLF WAP domain-containing protein OS=Canis lupus familiaris OX=9615 PE=4 SV=2	1.33	1.31	221.5	548065.3	2	1	0	14.66	116	21717
>tr|A0A5F4D6L9|A0A5F4D6L9_CANLF Sacsin molecular chaperone OS=Canis lupus familiaris OX=9615 GN=SACS PE=4 SV=1	1.02	0.85	229.7	92992927	17	4	1	0.33	4500	1444
>sp|O18840|ACTB_CANLF Actin, cytoplasmic 1 OS=Canis lupus familiaris OX=9615 GN=ACTB PE=2 SV=3	2.55	1.19	236.4	90298869.2	14	3	0	9.87	375	642
>tr|E2RG75|E2RG75_CANLF Inactive ribonuclease-like protein 9 OS=Canis lupus familiaris OX=9615 GN=RNASE9 PE=3 SV=2	3.36	3.18	276.5	28260980.6	7	2	1	8.08	198	41734
>tr|Q9XSV4|Q9XSV4_CANLF CE10 protein OS=Canis lupus familiaris OX=9615 GN=ce10 PE=2 SV=1	5.67	3.77	288	205217653.1	30	3	0	12.73	110	41542
>tr|F1PGF9|F1PGF9_CANLF Rho guanine nucleotide exchange factor 26 OS=Canis lupus familiaris OX=9615 GN=ARHGEF26 PE=4 SV=3	1.39	1.29	320.3	87856755.1	6	1	0	0.84	594	22876
>tr|F1PR54|F1PR54_CANLF Lactotransferrin OS=Canis lupus familiaris OX=9615 GN=LTF PE=3 SV=1	9.42	3.49	352	328762076.3	51	9	1	11.02	708	40436
>sp|Q28895|NPC2_CANLF NPC intracellular cholesterol transporter 2 OS=Canis lupus familiaris OX=9615 GN=NPC2 PE=2 SV=1	2.24	1.19	354.1	542889.1	5	2	0	14.77	149	153
>sp|Q9XS65|PTGDS_CANLF Prostaglandin-H2 D-isomerase OS=Canis lupus familiaris OX=9615 GN=PTGDS PE=2 SV=1	7.98	2.36	377.4	474370331.8	47	5	1	14.14	191	165
>tr|A0A5F4DBL7|A0A5F4DBL7_CANLF LDL receptor related protein 1 OS=Canis lupus familiaris OX=9615 GN=LRP1 PE=3 SV=1	0.42	0.42	32.6	3070857.8	1	1	0	0.22	4133	14011

>sp|A2IBY8|MIP_CANLF Lens fiber major intrinsic protein OS=Canis lupus familiaris OX=9615 GN=MIP PE=2 SV=1	0.2	0.2	44.9	1412721.4	1	1	0	1.9	263	112
>tr|E2RLU6|E2RLU6_CANLF G-protein coupled receptor 139 OS=Canis lupus familiaris OX=9615 GN=GPR139 PE=2 SV=1	0.35	0.35	52.4	18492944.3	1	1	0	6.82	352	41386
>tr|E2R9Y2|E2R9Y2_CANLF GB1/RHD3-type G domain-containing protein OS=Canis lupus familiaris OX=9615 GN=GBP1 PE=3 SV=3	0.39	0.35	70.4	955029	3	1	0	1.52	591	24472
>tr|J9P3D0|J9P3D0_CANLF Solute carrier family 4 member 9 OS=Canis lupus familiaris OX=9615 GN=SLC4A9 PE=3 SV=2	0.47	0.47	76.4	177288.7	1	1	0	1.12	893	31921
>sp|Q2PQH8|GDE_CANLF Glycogen debranching enzyme OS=Canis lupus familiaris OX=9615 GN=AGL PE=2 SV=1	0.7	0.34	78.1	168347768.2	13	2	0	0.26	1533	23
>tr|F1PBJ1|F1PBJ1_CANLF Methylcytosine dioxygenase TET OS=Canis lupus familiaris OX=9615 GN=TET3 PE=3 SV=2	0.16	0.02	78.5	19810784.1	8	1	0	0.28	1795	1529
>tr|A0A5F4BUA6|A0A5F4BUA6_CANLF NCK associated protein 5 OS=Canis lupus familiaris OX=9615 GN=NCKAP5 PE=4 SV=1	0.13	0.14	84.3	18095631.5	1	1	0	0.66	1977	5785
>tr|A0A5F4D0U7|A0A5F4D0U7_CANLF Structural maintenance of chromosomes flexible hinge domain containing 1 OS=Canis lupus familiaris OX=9615 GN=SMCHD1 PE=4 SV=1	0.35	0.35	84.8	977352.5	1	1	0	0.16	3051	9634
>tr|A0A5F4D0W4|A0A5F4D0W4_CANLF Glutathione-dependent dehydroascorbate reductase OS=Canis lupus familiaris OX=9615 GN=GSTO2 PE=3 SV=1	0.17	0.17	87.7	11986125.6	1	1	0	4.15	337	26941

>tr|A0A5F4C546|A0A5F4C546_CANLF Solute carrier family 25 member 17 OS=Canis lupus familiaris OX=9615 GN=SLC25A17 PE=3 SV=1	0.38	0.38	97.6	17096286.3	1	1	0	5.83	223	39398
>tr|A0A5F4BYT5|A0A5F4BYT5_CANLF Tyrosine-protein kinase OS=Canis lupus familiaris OX=9615 GN=ZAP70 PE=3 SV=1	0.43	0.35	99.4	47983643.3	5	1	0	3.72	592	15350
>sp|Q5QQ50|XYLT2_CANLF Xylosyltransferase 2 OS=Canis lupus familiaris OX=9615 GN=XYLT2 PE=2 SV=1	0.2	0.18	99.8	19874604.6	2	1	0	0.35	865	388
>sp|P62286|ASPM_CANLF Abnormal spindle-like microcephaly-associated protein homolog OS=Canis lupus familiaris OX=9615 GN=ASPM PE=2 SV=2	0.94	0.36	101.2	57685539.8	6	4	0	0.32	3469	677
>tr|A0A5F4DCZ9|A0A5F4DCZ9_CANLF Kinase D interacting substrate 220 OS=Canis lupus familiaris OX=9615 GN=KIDINS220 PE=4 SV=1	0.16	0.16	101.2	12938678.3	1	1	0	0.6	1678	1923
>sp|F1PRN2|MYO1D_CANLF Unconventional myosin-Id OS=Canis lupus familiaris OX=9615 GN=MYO1D PE=1 SV=2	0.37	0.37	102.7	12925775.1	1	1	0	0.3	1006	763
>tr|A0A5F4C0S7|A0A5F4C0S7_CANLF HEAT repeat containing 5A OS=Canis lupus familiaris OX=9615 GN=HEATR5A PE=3 SV=1	0.22	0.12	108.6	6105671.2	6	1	0	0.25	1995	1753
>tr|A0A5F4D824|A0A5F4D824_CANLF Zinc finger RANBP2-type containing 3 OS=Canis lupus familiaris OX=9615 GN=ZRANB3 PE=4 SV=1	0.37	0.37	108.8	8389955.8	1	1	0	0.53	1128	1130
>sp|O46669|SCNAA_CANLF Sodium channel protein type 10 subunit alpha OS=Canis lupus familiaris OX=9615 GN=SCN10A PE=2 SV=1	0.35	0.36	111.5	10351897.6	1	1	0	0.15	1962	90
>tr|A0A5F4CK78|A0A5F4CK78_CANLF NIMA related kinase 9 OS=Canis lupus familiaris OX=9615 GN=NEK9 PE=3 SV=1	0.17	0.15	114.3	3538761.4	2	1	0	1.25	1040	34954

>tr|A0A5F4C730|A0A5F4C730_CANLF Semaphorin 4D OS=Canis lupus familiaris OX=9615 GN=SEMA4D PE=3 SV=1	0.23	0.15	124.2	47685286.2	5	1	0	0.28	1067	1802
>tr|E2QY55|E2QY55_CANLF Isocitrate dehydrogenase [NAD] subunit, mitochondrial OS=Canis lupus familiaris OX=9615 GN=IDH3G PE=3 SV=3	0.15	0.15	134.6	10546175.6	1	1	0	2.3	392	21772
>tr|A0A5F4CMF9|A0A5F4CMF9_CANLF Cilia and flagella associated protein 65 OS=Canis lupus familiaris OX=9615 GN=CFAP65 PE=4 SV=1	0.36	0.36	139.3	6548062.7	1	1	0	0.17	1789	4175
>tr|F1P884|F1P884_CANLF PiggyBac transposable element derived 5 OS=Canis lupus familiaris OX=9615 GN=PGBD5 PE=4 SV=3	0.24	0.12	141.6	107595841.1	7	1	0	2.4	458	28672
>tr|A0A5F4DD58|A0A5F4DD58_CANLF Phosphoinositide phospholipase C OS=Canis lupus familiaris OX=9615 GN=PLCD3 PE=4 SV=1	0.42	0.36	144.7	16940464.9	5	2	0	0.94	741	3088
>tr|A0A5F4BQE1|A0A5F4BQE1_CANLF Vacuolar fusion protein MON1 homolog OS=Canis lupus familiaris OX=9615 GN=MON1B PE=3 SV=1	0.67	0.67	145.6	16642869.2	1	1	0	2.32	561	9974
>sp|P23685|NAC1_CANLF Sodium/calcium exchanger 1 OS=Canis lupus familiaris OX=9615 GN=SLC8A1 PE=1 SV=1	0.35	0.36	147.3	12080140.7	1	1	0	1.03	970	764
>tr|F1PI09|F1PI09_CANLF Aldehyde oxidase OS=Canis lupus familiaris OX=9615 GN=AOX2 PE=3 SV=3	0.41	0.37	147.4	2327470.8	3	1	0	0.67	1347	21650
>tr|F1P8J6|F1P8J6_CANLF RNA helicase OS=Canis lupus familiaris OX=9615 GN=DDX55 PE=3 SV=3	0.28	0.24	147.6	44211263.8	3	1	0	1.58	568	8934
>tr|F1PLT8|F1PLT8_CANLF Sulfhydryl oxidase OS=Canis lupus familiaris OX=9615 GN=QSOX1 PE=3 SV=3	0.11	0.09	148.9	21541774.5	2	1	0	2.64	568	33056

>tr|cc|E2RSV7_CANLF Nuclear pore complex protein Nup93 OS=Canis lupus familiaris OX=9615 GN=NUP93 PE=3 SV=2	0.43	0.36	151.3	17288988.2	5	2	1	2.16	925	13752
>tr|J9P0B4|J9P0B4_CANLF Tudor domain containing 15 OS=Canis lupus familiaris OX=9615 GN=TDRD15 PE=4 SV=2	0.1	0.01	151.5	19087152.4	9	1	0	0.57	2105	4188
>tr|A0A5F4D0B3|A0A5F4D0B3_CANLF Bromodomain containing 1 OS=Canis lupus familiaris OX=9615 GN=BRD1 PE=4 SV=1	0.19	0.17	154.3	34688350.5	2	1	0	0.81	1112	1580
>tr|F1PTW3|F1PTW3_CANLF KIAA0753 OS=Canis lupus familiaris OX=9615 GN=KIAA0753 PE=4 SV=3	0.41	0.37	155.9	8765844.2	3	1	0	0.88	905	33899
>tr|F1PGX9|F1PGX9_CANLF Solute carrier family 2 member 13 OS=Canis lupus familiaris OX=9615 GN=SLC2A13 PE=3 SV=3	0.13	0.13	157.4	14454989	1	1	0	0.96	624	37617
>tr|F1PHQ0|F1PHQ0_CANLF Clathrin heavy chain OS=Canis lupus familiaris OX=9615 GN=CLTC PE=3 SV=3	0.16	0.14	159.4	22360460.1	2	1	1	0.65	1682	24646
>tr|F1Q0U7|F1Q0U7_CANLF ENAH actin regulator OS=Canis lupus familiaris OX=9615 GN=ENAH PE=3 SV=3	0.13	0.13	161.3	11109991.5	1	1	0	1.6	810	976
>tr|A0A5F4D430|A0A5F4D430_CANLF Transcription factor AP-2 gamma OS=Canis lupus familiaris OX=9615 GN=TFAP2C PE=3 SV=1	0.6	0.61	162	2499781.8	1	1	0	1.38	650	9642
>sp|Q6AW47|EST5A_CANLF Carboxylesterase 5A OS=Canis lupus familiaris OX=9615 GN=CES5A PE=2 SV=1	0.59	0.53	164.1	37613594.5	3	2	0	3.83	575	629
>tr|A0A5F4DDS5|A0A5F4DDS5_CANLF CD86 molecule OS=Canis lupus familiaris OX=9615 GN=CD86 PE=4 SV=1	0.49	0.47	164.6	20983189.6	2	1	0	2.22	315	17545

>tr|F1PBU5|F1PBU5_CANLF Non-specific serine/threonine protein kinase OS=Canis lupus familiaris OX=9615 GN=SMG1 PE=3 SV=3	0.36	0.31	167.7	33714348.1	4	1	0	0.08	3634	6898
>tr|A0A5F4D848|A0A5F4D848_CANLF Lipase I OS=Canis lupus familiaris OX=9615 GN=LIPI PE=3 SV=1	0.17	0.15	170.3	26274176.5	2	1	0	2.09	431	1169
>sp|Q5TJE1|DAXX_CANLF Death domain-associated protein 6 OS=Canis lupus familiaris OX=9615 GN=DAXX PE=3 SV=1	0.34	0.33	171.3	12837981.6	2	1	0	0.41	737	429
>tr|A0A5F4CCD0|A0A5F4CCD0_CANLF Cysteine rich secretory protein 2 OS=Canis lupus familiaris OX=9615 GN=CRISP2 PE=3 SV=1	0.13	0.13	174.1	13099870.7	1	1	0	2.25	311	11017
>tr|A0A5F4CHL0|A0A5F4CHL0_CANLF Anoctamin OS=Canis lupus familiaris OX=9615 GN=ANO9 PE=3 SV=1	0.65	0.39	176.5	302257152	14	1	0	1.82	824	37323
>sp|P06625|SRPRA_CANLF Signal recognition particle receptor subunit alpha OS=Canis lupus familiaris OX=9615 GN=SRPRA PE=2 SV=2	0.14	0.05	179.1	43747251.5	5	3	0	2.82	638	369
>tr|A0A5F4DK55|A0A5F4DK55_CANLF Reverse transcriptase domain-containing protein OS=Canis lupus familiaris OX=9615 PE=4 SV=1	0.5	0.23	180.5	81982953.1	7	2	0	0.55	1275	1651
>tr|A0A5K1V0D8|A0A5K1V0D8_CANLF Sulfatase 2 OS=Canis lupus familiaris OX=9615 GN=SULF2 PE=3 SV=1	0.42	0.17	181.6	157954121.7	15	2	0	0.81	859	1192
>tr|F1Q4I7|F1Q4I7_CANLF Beta-1,4-N-acetylgalactosaminyltransferase OS=Canis lupus familiaris OX=9615 GN=B4GALNT3 PE=3 SV=3	0.38	0.38	183.5	10351897.6	1	1	0	0.4	1003	6004
>tr|A0A5F4BXD8|A0A5F4BXD8_CANLF Matrix metallopeptidase 16 OS=Canis lupus familiaris OX=9615 GN=MMP16 PE=3 SV=1	0.45	0.43	183.9	37714034.6	2	1	0	2.65	566	6476

>tr|A0A5F4BZW4|A0A5F4BZW4_CANLF Malonyl-CoA decarboxylase OS=Canis lupus familiaris OX=9615 GN=MLYCD PE=4 SV=1	0.48	0.34	187	103394674.3	8	1	0	1.3	461	4809
>tr|Q9XSV4|Q9XSV4_CANLF CE10 protein OS=Canis lupus familiaris OX=9615 GN=ce10 PE=2 SV=1	1.04	0.4	187.2	47663466.3	15	3	0	12.73	110	41542
>tr|E2RN16|E2RN16_CANLF Mitogen-activated protein kinase kinase kinase 2 OS=Canis lupus familiaris OX=9615 GN=MAP3K2 PE=4 SV=2	0.4	0.34	188.3	49798235.8	4	1	0	0.97	620	34325
>tr|F1PA52|F1PA52_CANLF Hyperpolarization activated cyclic nucleotide gated potassium and sodium channel 2 OS=Canis lupus familiaris OX=9615 GN=HCN2 PE=3 SV=3	0.54	0.54	191.2	13158030.9	1	1	0	0.73	821	34579
>sp|Q9GL25|ESPB1_CANLF Epididymal sperm-binding protein 1 OS=Canis lupus familiaris OX=9615 GN=ELSPBP1 PE=1 SV=1	1.22	1.22	194.2	9429510.7	1	1	0	6.94	245	36
>tr|A0A5F4CI67|A0A5F4CI67_CANLF Mannanase OS=Canis lupus familiaris OX=9615 GN=MANBA PE=4 SV=1	0.64	0.62	197.5	15157777.5	2	1	0	1.45	828	37800
>tr|A0A5F4D9S5|A0A5F4D9S5_CANLF Hyaluronoglucosaminidase OS=Canis lupus familiaris OX=9615 GN=CEMIP PE=3 SV=1	0.81	0.57	198.2	187265362.7	13	1	0	0.24	1684	9775
>tr|E2R6E0|E2R6E0_CANLF Lipocln_cytosolic_FA-bd_dom domain-containing protein OS=Canis lupus familiaris OX=9615 GN=LCNL1 PE=3 SV=2	2.28	2.18	228.1	64505297.6	6	1	0	3.01	299	1932
>tr|E2RN56|E2RN56_CANLF Zinc finger CCCH-type containing 13 OS=Canis lupus familiaris OX=9615 GN=ZC3H13 PE=4 SV=3	0.58	0.54	228.5	32881674.4	3	1	0	0.24	1660	1446
>tr|A0A5F4CJI0|A0A5F4CJI0_CANLF Folate_rec domain-containing protein OS=Canis lupus familiaris OX=9615 GN=FOLR1 PE=3 SV=1	0.61	0.57	231.2	28756277.6	3	1	0	3.14	255	10075

>sp|O18840|ACTB_CANLF Actin, cytoplasmic 1 OS=Canis lupus familiaris OX=9615 GN=ACTB PE=2 SV=3	1.94	1.72	238.1	103375480.6	12	1	0	2.93	375	642
>tr|A0A5F4D6L9|A0A5F4D6L9_CANLF Sacsin molecular chaperone OS=Canis lupus familiaris OX=9615 GN=SACS PE=4 SV=1	1.31	0.38	241.3	305031485.1	27	4	0	0.53	4500	1444
>sp|P49822|ALBU_CANLF Albumin OS=Canis lupus familiaris OX=9615 GN=ALB PE=1 SV=3	3.43	2.97	251.9	72775789.7	7	3	0	6.91	608	490
>tr|A0A5F4D6G2|A0A5F4D6G2_CANLF SMG7 nonsense mediated mRNA decay factor OS=Canis lupus familiaris OX=9615 GN=SMG7 PE=4 SV=1	0.47	0.45	267.8	16124928.9	2	1	0	0.43	1175	2075
>tr|F1PJ71|F1PJ71_CANLF Glutathione peroxidase OS=Canis lupus familiaris OX=9615 GN=GPX5 PE=3 SV=2	1.47	0.58	271.1	122645604.4	14	4	1	15.84	221	19009
>sp|Q9XS65|PTGDS_CANLF Prostaglandin-H2 D-isomerase OS=Canis lupus familiaris OX=9615 GN=PTGDS PE=2 SV=1	2.19	1.42	297.6	149308819	12	2	0	10.47	191	165
>tr|E2RCT1|E2RCT1_CANLF WAP domain-containing protein OS=Canis lupus familiaris OX=9615 PE=4 SV=2	6.73	3.2	315.3	602951558.5	30	2	0	9.48	116	21717
>tr|Q30KS5|Q30KS5_CANLF Beta-defensin 129 OS=Canis lupus familiaris OX=9615 GN=DEFB129 PE=2 SV=1	2.65	2.49	340.7	137028954.2	9	1	0	4.22	166	41730
>tr|A0A5F4BVF3|A0A5F4BVF3_CANLF Lactotransferrin OS=Canis lupus familiaris OX=9615 GN=LTF PE=3 SV=1	6.26	2.91	378.9	949659431.2	67	9	0	15.97	626	32850
>sp|Q28895|NPC2_CANLF NPC intracellular cholesterol transporter 2 OS=Canis lupus familiaris OX=9615 GN=NPC2 PE=2 SV=1	10.34	3.97	448.1	131596555.2	39	5	0	30.2	149	153

>tr|E2QXW6|E2QXW6_CANLF Patatin like phospholipase domain containing 7 OS=Canis lupus familiaris OX=9615 GN=PNPLA7 PE=3 SV=2	0.1	0.06	35.9	33412088.3	3	1	1	1.58	1456	31798
>tr|J9NS28|J9NS28_CANLF RBR-type E3 ubiquitin transferase OS=Canis lupus familiaris OX=9615 GN=ANKIB1 PE=4 SV=2	0.23	0.19	37	34454786.8	3	1	0	2.33	988	26345
>tr|E2RTL2|E2RTL2_CANLF Tubulin tyrosine ligase like 6 OS=Canis lupus familiaris OX=9615 GN=TTLL6 PE=4 SV=3	0.1	0.07	37.3	7928933.2	1	1	0	0.6	827	2703
>tr|A0A5F4DFY1|A0A5F4DFY1_CANLF SHH signaling and ciliogenesis regulator SDCCAG8 OS=Canis lupus familiaris OX=9615 GN=SDCCAG8 PE=4 SV=1	0.1	0.01	39.3	10965869.4	11	1	1	0.59	673	17765
>tr|F1PGL2|F1PGL2_CANLF RNA helicase OS=Canis lupus familiaris OX=9615 GN=DHX16 PE=4 SV=2	0.15	0.13	40.1	16524257	2	1	0	1.73	1042	43089
>tr|A0A5F4C289|A0A5F4C289_CANLF Netrin receptor UNC5 OS=Canis lupus familiaris OX=9615 GN=UNC5D PE=3 SV=1	0.14	0.14	43.7	3257952.1	1	1	1	1.8	945	14524
>tr|E2RG96|E2RG96_CANLF Glutamate receptor OS=Canis lupus familiaris OX=9615 GN=GRIN2C PE=3 SV=3	0.33	0.33	44.7	7335816.2	1	1	0	1.9	1212	31148
>tr|E2R4F0|E2R4F0_CANLF Cadherin EGF LAG seven-pass G-type receptor 2 OS=Canis lupus familiaris OX=9615 GN=CELSR2 PE=3 SV=2	0.21	0.22	46.3	7548313.9	1	1	1	0.65	2919	12898
>tr|E2RIH1|E2RIH1_CANLF DOP1 leucine zipper like protein B OS=Canis lupus familiaris OX=9615 GN=DOP1B PE=3 SV=3	1.18	1.18	47.3	17933746.1	1	1	0	1.2	2259	7751
>tr|A0A5F4D9N8|A0A5F4D9N8_CANLF Procollagen-lysine,2-oxoglutarate 5-dioxygenase 3 OS=Canis lupus familiaris OX=9615 GN=PLOD3 PE=4 SV=1	0.16	0.16	53.3	4849194.1	1	1	0	2.17	785	1958

>tr|E2RQR2|E2RQR2_CANLF Olfactory receptor family 13 subfamily P member 3 OS=Canis lupus familiaris OX=9615 GN=OR13P3 PE=4 SV=2	0.3	0.3	54.5	8458140.2	1	1	0	6.73	312	15310
>tr|A0A5F4CAN7|A0A5F4CAN7_CANLF RBR-type E3 ubiquitin transferase OS=Canis lupus familiaris OX=9615 GN=RNF144A PE=4 SV=1	0.1	0.07	59.2	6725146.3	1	1	1	2.31	347	23811
>tr|F1PIW3|F1PIW3_CANLF Dynein axonemal heavy chain 14 OS=Canis lupus familiaris OX=9615 GN=DNAH14 PE=3 SV=3	0.11	0.1	69.6	3274474.5	2	1	1	0.16	4491	30490
>tr|J9JH98|J9JH98_CANLF Olfactory receptor family 1 subfamily L member 6 OS=Canis lupus familiaris OX=9615 GN=OR1L6 PE=4 SV=1	0.11	0.11	82.6	8909188.5	1	1	1	6.77	310	3096
>sp|P21842|CMA1_CANLF Chymase OS=Canis lupus familiaris OX=9615 GN=CMA1 PE=1 SV=1	0.64	0.6	88.2	6108504.4	3	1	0	0.8	249	34
>tr|F1PI09|F1PI09_CANLF Aldehyde oxidase OS=Canis lupus familiaris OX=9615 GN=AOX2 PE=3 SV=3	0.13	0.13	88.3	1775376.8	1	1	0	0.67	1347	21650
>tr|J9NW72|J9NW72_CANLF Sperm associated antigen 8 OS=Canis lupus familiaris OX=9615 GN=SPAG8 PE=4 SV=1	0.58	0.58	91.7	6338436.5	1	1	0	4.07	442	10371
>sp|Q9MZY0|CP2E1_CANLF Cytochrome P450 2E1 OS=Canis lupus familiaris OX=9615 GN=CYP2E1 PE=2 SV=1	0.1	0.02	92.2	90796602.3	9	1	1	4.05	494	522
>tr|A0A5F4D9Z7|A0A5F4D9Z7_CANLF Zinc finger FYVE-type containing 19 OS=Canis lupus familiaris OX=9615 GN=ZFYVE19 PE=4 SV=1	0.1	0.07	93.4	23186453.4	2	1	0	1.53	392	13875
>tr|F1PEE8|F1PEE8_CANLF Ryanodine receptor 2 OS=Canis lupus familiaris OX=9615 GN=RYR2 PE=4 SV=3	0.16	0.17	95	7402159.8	1	1	0	0.22	4903	13641

>tr|F1PLU0|F1PLU0_CANLF Histone-lysine N-methyltransferase OS=Canis lupus familiaris OX=9615 GN=KMT2A PE=3 SV=3	0.13	0.12	95.3	9933961.8	2	1	1	0.34	3822	11664
>sp|P83509|RHG35_CANLF Rho GTPase-activating protein 35 OS=Canis lupus familiaris OX=9615 GN=ARHGAP35 PE=2 SV=1	0.42	0.4	98.1	10086811.8	2	1	1	0.73	1500	323
>sp|Q8WN22|PRKDC_CANLF DNA-dependent protein kinase catalytic subunit OS=Canis lupus familiaris OX=9615 GN=PRKDC PE=2 SV=1	1.85	1.39	101.6	8643845.6	3	2	0	0.12	4144	338
>tr|J9P0B4|J9P0B4_CANLF Tudor domain containing 15 OS=Canis lupus familiaris OX=9615 GN=TDRD15 PE=4 SV=2	1.48	0.89	105.1	11191609.5	3	2	0	1.85	2105	4188
>tr|E2RK34|E2RK34_CANLF Cytochrome P450 family 2 subfamily S member 1 OS=Canis lupus familiaris OX=9615 GN=CYP2S1 PE=3 SV=3	0.43	0.43	107.3	10797590.7	1	1	0	1.35	669	7130
>tr|F1PPP9|F1PPP9_CANLF Family with sequence similarity 135 member A OS=Canis lupus familiaris OX=9615 GN=FAM135A PE=3 SV=3	0.84	0.78	111.3	7288820.4	4	1	0	1.22	1399	6815
>sp|E2RKA8|RL32_CANLF 60S ribosomal protein L32 OS=Canis lupus familiaris OX=9615 GN=RPL32 PE=1 SV=1	1.24	0.02	111.4	460727872.2	89	2	0	4.44	135	275
>sp|Q9TU69|GHR_CANLF Growth hormone receptor OS=Canis lupus familiaris OX=9615 GN=GHR PE=2 SV=1	0.21	0.19	112.6	16417944.7	2	1	0	1.72	638	541
>tr|J9P432|J9P432_CANLF Glutamine--fructose-6-phosphate transaminase (isomerizing) OS=Canis lupus familiaris OX=9615 GN=GFPT1 PE=4 SV=2	0.33	0.19	113.5	30126452.3	8	1	0	1.18	677	7191
>tr|A0A5F4CI07|A0A5F4CI07_CANLF RAB3 GTPase activating non-catalytic protein subunit 2 OS=Canis lupus familiaris OX=9615 GN=RAB3GAP2 PE=3 SV=1	0.13	0.13	113.7	6213974	1	1	0	0.79	1515	5216

>tr|E2R824|E2R824_CANLF Zinc finger protein 518B OS=Canis lupus familiaris OX=9615 GN=ZNF518B PE=4 SV=3	0.13	0.11	121	11390595.8	2	1	0	0.8	1000	24468
>tr|A0A5F4D8I6|A0A5F4D8I6_CANLF Phospholipase A2 receptor 1 OS=Canis lupus familiaris OX=9615 GN=PLA2R1 PE=4 SV=1	0.53	0.41	121.6	16419922.9	7	1	0	0.5	1394	6796
>tr|A0A5F4D2W3|A0A5F4D2W3_CANLF Growth factor receptor bound protein 2 OS=Canis lupus familiaris OX=9615 GN=GRB2 PE=4 SV=1	0.15	0.13	123.4	15860300	2	2	2	4.95	222	13178
>tr|F1P8E3|F1P8E3_CANLF Glutamate metabotropic receptor 6 OS=Canis lupus familiaris OX=9615 GN=GRM6 PE=3 SV=3	0.16	0.16	130.2	10389980	1	1	0	0.68	876	36035
>tr|Q2A652|Q2A652_CANLF G-protein coupled receptor OS=Canis lupus familiaris OX=9615 GN=PTGDR2 PE=2 SV=1	0.83	0.83	131.5	7904084.5	1	1	0	1.25	400	41469
>tr|A0A5F4CUE8|A0A5F4CUE8_CANLF Senataxin OS=Canis lupus familiaris OX=9615 GN=SETX PE=4 SV=1	0.11	0.11	133.2	1946556.2	1	1	0	0.26	2645	1796
>tr|E2RDB0|E2RDB0_CANLF Phosphate regulating endopeptidase homolog X-linked OS=Canis lupus familiaris OX=9615 GN=PHEX PE=4 SV=2	0.1	0.07	135.9	6320761.7	1	1	1	1.34	749	2968
>tr|E2RCT1|E2RCT1_CANLF WAP domain-containing protein OS=Canis lupus familiaris OX=9615 PE=4 SV=2	1.47	1.4	135.9	40419871.7	3	2	0	9.48	116	21717
>tr|A0A5F4CGE0|A0A5F4CGE0_CANLF Ubiquitin protein ligase E3C OS=Canis lupus familiaris OX=9615 GN=UBE3C PE=4 SV=1	0.36	0.36	137.2	9093807.6	1	1	0	0.97	1238	9018
>tr|A0A5F4CQW0|A0A5F4CQW0_CANLF Polybromo 1 OS=Canis lupus familiaris OX=9615 GN=PBRM1 PE=4 SV=1	0.21	0.19	140.2	20883191.9	2	1	0	0.79	1510	2204

>tr|A0A5F4CLU1|A0A5F4CLU1_CANLF Superoxide dismutase [Cu-Zn] OS=Canis lupus familiaris OX=9615 GN=SOD1 PE=3 SV=1	0.35	0.35	143.8	2338204.7	1	1	0	7.09	141	25417
>tr|F1PFZ5|F1PFZ5_CANLF Milk fat globule EGF and factor V/VIII domain containing OS=Canis lupus familiaris OX=9615 GN=MFGE8 PE=4 SV=3	0.25	0.25	147.5	6719153.7	1	1	0	1.87	428	7079
>tr|A0A5F4CCD0|A0A5F4CCD0_CANLF Cysteine rich secretory protein 2 OS=Canis lupus familiaris OX=9615 GN=CRISP2 PE=3 SV=1	0.1	0.07	152.9	5872668.4	1	1	0	2.25	311	11017
>tr|A0A5F4DCA4|A0A5F4DCA4_CANLF Reverse transcriptase domain-containing protein OS=Canis lupus familiaris OX=9615 PE=4 SV=1	1.26	1.26	176.4	1998374.1	1	1	0	0.31	978	860
>tr|F1PBU5|F1PBU5_CANLF Non-specific serine/threonine protein kinase OS=Canis lupus familiaris OX=9615 GN=SMG1 PE=3 SV=3	1.51	0.81	177.2	40793140.9	10	2	0	0.17	3634	6898
>sp|Q9GL25|ESPB1_CANLF Epididymal sperm-binding protein 1 OS=Canis lupus familiaris OX=9615 GN=ELSPBP1 PE=1 SV=1	1.76	1.74	182.3	4263954.6	2	1	0	4.49	245	36
>sp|P38377|S61A1_CANLF Protein transport protein Sec61 subunit alpha isoform 1 OS=Canis lupus familiaris OX=9615 GN=SEC61A1 PE=1 SV=3	0.82	0.8	182.9	7286196.4	2	1	0	0.84	476	358
>tr|A0A5F4D7J3|A0A5F4D7J3_CANLF Non-specific serine/threonine protein kinase OS=Canis lupus familiaris OX=9615 GN=CDC42BPA PE=3 SV=1	0.8	0.8	183.6	4334414.5	1	1	0	0.22	1794	1069
>tr|J9NS29|J9NS29_CANLF Cystatin domain-containing protein OS=Canis lupus familiaris OX=9615 GN=LOC607874 PE=4 SV=2	0.77	0.75	186.8	13060868.3	2	1	0	6.39	313	30016
>tr|A0A5F4D9S5|A0A5F4D9S5_CANLF Hyaluronoglucosaminidase OS=Canis lupus familiaris OX=9615 GN=CEMIP PE=3 SV=1	0.55	0.55	187.3	4458486.7	1	1	0	0.24	1684	9775

>tr|J9NZH4|J9NZH4_CANLF NTR domain-containing protein OS=Canis lupus familiaris OX=9615 GN=LOC102154527 PE=3 SV=2	0.27	0.25	188.5	16404036.1	2	1	0	8.14	221	39505
>tr|E2R6E0|E2R6E0_CANLF Lipocln_cytosolic_FA-bd_dom domain-containing protein OS=Canis lupus familiaris OX=9615 GN=LCNL1 PE=3 SV=2	1.71	1.55	189	37651996.4	9	1	0	3.68	299	1932
>tr|A0A5F4D6G2|A0A5F4D6G2_CANLF SMG7 nonsense mediated mRNA decay factor OS=Canis lupus familiaris OX=9615 GN=SMG7 PE=4 SV=1	0.19	0.19	193.4	4676067.7	1	1	0	0.43	1175	2075
>tr|A0A5F4D7Y5|A0A5F4D7Y5_CANLF Pleckstrin homology, MyTH4 and FERM domain containing H1 OS=Canis lupus familiaris OX=9615 GN=PLEKHH1 PE=4 SV=1	0.34	0.33	194.4	10122263.9	2	1	0	0.3	1342	5979
>tr|A0A5F4CHN1|A0A5F4CHN1_CANLF Serine/arginine repetitive matrix 2 OS=Canis lupus familiaris OX=9615 GN=SRRM2 PE=4 SV=1	1.55	1.24	197.7	11997739.7	6	2	0	0.27	2564	3180
>tr|A0A5F4C1S8|A0A5F4C1S8_CANLF E3 ubiquitin-protein ligase CBL OS=Canis lupus familiaris OX=9615 GN=CBL PE=4 SV=1	1.5	0.48	198.4	330371188.7	50	2	0	0.91	773	1308
>sp|P49822|ALBU_CANLF Albumin OS=Canis lupus familiaris OX=9615 GN=ALB PE=1 SV=3	0.11	0.05	208.2	25738417	4	1	0	2.3	608	490
>sp|E2RED8|AP4M1_CANLF AP-4 complex subunit mu-1 OS=Canis lupus familiaris OX=9615 GN=AP4M1 PE=3 SV=2	0.3	0.23	209.4	21406036.1	5	1	0	0.66	452	634
>sp|B8K1W2|ABCBB_CANLF Bile salt export pump OS=Canis lupus familiaris OX=9615 GN=Abcb11e PE=1 SV=1	0.85	0.81	211	11024350	3	1	0	0.3	1325	527
>tr|A0A5F4BX58|A0A5F4BX58_CANLF Transcription termination factor 1 OS=Canis lupus familiaris OX=9615 GN=TTF1 PE=4 SV=1	0.76	0.74	214.7	3896328.8	2	1	0	0.59	677	12089

>sp|O46607|GPX5_CANLF Epididymal secretory glutathione peroxidase OS=Canis lupus familiaris OX=9615 GN=GPX5 PE=2 SV=1	1.01	0.85	233.1	15096457.5	4	2	0	8.6	221	564
>sp|Q6AW47|EST5A_CANLF Carboxylesterase 5A OS=Canis lupus familiaris OX=9615 GN=CES5A PE=2 SV=1	0.42	0.38	237.1	17411428.4	3	1	0	0.87	575	629
>sp|P25473|CLUS_CANLF Clusterin OS=Canis lupus familiaris OX=9615 GN=CLU PE=2 SV=1	0.98	0.98	240	2016485.8	1	1	0	0.9	445	725
>tr|Q9XSV4|Q9XSV4_CANLF CE10 protein OS=Canis lupus familiaris OX=9615 GN=ce10 PE=2 SV=1	1.99	1.83	273.7	27127890.7	7	2	0	9.09	110	41542
>sp|Q9XS65|PTGDS_CANLF Prostaglandin-H2 D-isomerase OS=Canis lupus familiaris OX=9615 GN=PTGDS PE=2 SV=1	2.01	1.55	288.5	85454601.9	11	2	1	10.47	191	165
>tr|A0A5F4BVF3|A0A5F4BVF3_CANLF Lactotransferrin OS=Canis lupus familiaris OX=9615 GN=LTF PE=3 SV=1	12.5	3.24	332	628759365.5	76	10	0	11.5	626	32850
>sp|O18840|ACTB_CANLF Actin, cytoplasmic 1 OS=Canis lupus familiaris OX=9615 GN=ACTB PE=2 SV=3	4.29	3.13	343.4	208868607.2	18	2	0	5.33	375	642
>sp|Q28894|WFDC2_CANLF WAP four-disulfide core domain protein 2 OS=Canis lupus familiaris OX=9615 GN=WFDC2 PE=2 SV=1	1.79	1.77	348.7	2311834	2	1	0	6.45	124	53
>tr|E2RG75|E2RG75_CANLF Inactive ribonuclease-like protein 9 OS=Canis lupus familiaris OX=9615 GN=RNASE9 PE=3 SV=2	0.84	0.82	353	2240387	2	1	1	4.55	198	41734
>sp|Q28895|NPC2_CANLF NPC intracellular cholesterol transporter 2 OS=Canis lupus familiaris OX=9615 GN=NPC2 PE=2 SV=1	11.28	5.59	422.9	182362786.5	25	4	0	30.2	149	153

>sp|Q9XSU7|RL27_CANLF 60S ribosomal protein L27 OS=Canis lupus familiaris OX=9615 GN=RPL27 PE=2 SV=3	0.1	0.02	35	5589067.7	4	1	0	3.68	136	314
>tr|E2RIV7|E2RIV7_CANLF Syntrophin alpha 1 OS=Canis lupus familiaris OX=9615 GN=SNTA1 PE=3 SV=3	0.1	0	41.5	5638575.8	3	1	1	1.03	486	34454
>tr|F1PS54|F1PS54_CANLF MFS domain-containing protein OS=Canis lupus familiaris OX=9615 GN=SLC17A3 PE=4 SV=3	0.1	0.01	42.5	38123286	2	1	1	7.71	493	988
>tr|J9NS28|J9NS28_CANLF RBR-type E3 ubiquitin transferase OS=Canis lupus familiaris OX=9615 GN=ANKIB1 PE=4 SV=2	0.56	0.5	46.2	86444910.9	4	1	0	2.33	988	26345
>tr|E2R868|E2R868_CANLF [histone H4]-N-methyl-L-lysine20 N-methyltransferase KMT5B OS=Canis lupus familiaris OX=9615 GN=KMT5B PE=4 SV=3	0.1	0.02	46.3	4869575.4	2	2	1	1.13	885	7704
>tr|A0A5F4C745|A0A5F4C745_CANLF HECT-type E3 ubiquitin transferase OS=Canis lupus familiaris OX=9615 GN=HERC2 PE=4 SV=1	0.1	0.02	47	3331439.9	2	1	0	0.1	4862	967
>tr|E2RRF5|E2RRF5_CANLF RNA binding motif protein 19 OS=Canis lupus familiaris OX=9615 GN=RBM19 PE=4 SV=3	0.1	0	56.1	118978307.2	49	2	0	0.72	970	905
>tr|J9P3H8|J9P3H8_CANLF ATM interactor OS=Canis lupus familiaris OX=9615 GN=ATMIN PE=4 SV=2	0.13	0.02	56.2	12633306.2	7	1	0	0.58	863	882
>tr|J9P432|J9P432_CANLF Glutamine--fructose-6-phosphate transaminase (isomerizing) OS=Canis lupus familiaris OX=9615 GN=GFPT1 PE=4 SV=2	0.92	0.92	59.6	4750361.1	1	1	0	1.18	677	7191
>tr|A0A5F4DHH0|A0A5F4DHH0_CANLF ATP binding cassette subfamily A member 1 OS=Canis lupus familiaris OX=9615 GN=ABCA1 PE=4 SV=1	0.17	0.02	60.1	21091293.3	10	1	0	0.23	2175	3709

>tr|A0A5F4CPU3|A0A5F4CPU3_CANLF SEC24 homolog D, COPII coat complex component OS=Canis lupus familiaris OX=9615 GN=SEC24D PE=3 SV=1	0.46	0.02	64	86457550	36	1	0	0.53	946	2125
>tr|A0A5F4C0S7|A0A5F4C0S7_CANLF HEAT repeat containing 5A OS=Canis lupus familiaris OX=9615 GN=HEATR5A PE=3 SV=1	0.33	0.02	65.4	52598492.9	23	1	0	0.25	1995	1753
>tr|F1PBJ1|F1PBJ1_CANLF Methylcytosine dioxygenase TET OS=Canis lupus familiaris OX=9615 GN=TET3 PE=3 SV=2	0.1	0.02	69.9	4573564.7	2	1	0	0.28	1795	1529
>tr|A0A5F4D595|A0A5F4D595_CANLF Zinc finger protein 483 OS=Canis lupus familiaris OX=9615 GN=ZNF483 PE=4 SV=1	0.33	0.33	70.4	26472339.2	1	1	0	3.6	750	3465
>tr|J9PAZ6|J9PAZ6_CANLF Hyperpolarization activated cyclic nucleotide gated potassium channel 4 OS=Canis lupus familiaris OX=9615 GN=HCN4 PE=4 SV=2	0.1	0	71.3	76259828.2	4	1	1	4.91	530	19838
>tr|J9NSS6|J9NSS6_CANLF DNA helicase OS=Canis lupus familiaris OX=9615 GN=CHD2 PE=4 SV=2	0.22	0.15	73.2	6784136.8	5	1	0	0.28	1780	1264
>tr|E2RE16|E2RE16_CANLF Non-specific serine/threonine protein kinase OS=Canis lupus familiaris OX=9615 GN=PAK4 PE=4 SV=1	0.65	0.02	80.4	112709410	43	1	0	0.84	592	12735
>tr|A0A5F4BQA2|A0A5F4BQA2_CANLF Dermatan sulfate epimerase OS=Canis lupus familiaris OX=9615 GN=DSE PE=3 SV=1	0.1	0	84.9	10466782.3	1	1	0	2.6	308	8924
>tr|E2RQR2|E2RQR2_CANLF Olfactory receptor family 13 subfamily P member 3 OS=Canis lupus familiaris OX=9615 GN=OR13P3 PE=4 SV=2	0.37	0.37	93	20957177.5	1	1	0	6.73	312	15310
>sp|P62286|ASPM_CANLF Abnormal spindle-like microcephaly-associated protein homolog OS=Canis lupus familiaris OX=9615 GN=ASPM PE=2 SV=2	1.18	1.1	94.1	258449448.8	29	2	0	0.14	3469	677

>sp|P21842|CMA1_CANLF Chymase OS=Canis lupus familiaris OX=9615 GN=CMA1 PE=1 SV=1	0.95	0.91	100	22126849	3	1	0	0.8	249	34
>tr|F1PGK9|F1PGK9_CANLF ADAM metallopeptidase with thrombospondin type 1 motif 5 OS=Canis lupus familiaris OX=9615 GN=ADAMTS5 PE=4 SV=3	0.34	0.15	105.9	23566169.6	12	1	0	0.59	845	11956
>tr|A0A5F4BT89|A0A5F4BT89_CANLF Olfactory receptor OS=Canis lupus familiaris OX=9615 GN=OR5W6 PE=3 SV=1	0.1	0	106.4	37127417.2	2	1	1	6.95	302	29923
>tr|E2R594|E2R594_CANLF Ring finger protein 167 OS=Canis lupus familiaris OX=9615 GN=RNF167 PE=4 SV=3	0.1	0	108.2	16461454.3	1	1	1	19.23	130	16405
>tr|F1PAJ9|F1PAJ9_CANLF Adenylate cyclase type 5 OS=Canis lupus familiaris OX=9615 GN=ADCY5 PE=3 SV=3	1.4	1.4	111.5	13972157.6	1	1	0	2.24	1027	18306
>tr|A0A5F4DFX0|A0A5F4DFX0_CANLF Exportin-T OS=Canis lupus familiaris OX=9615 GN=XPOT PE=3 SV=1	0.1	0.01	115.4	15713572.6	2	1	0	0.75	938	2329
>tr|J9NZH4|J9NZH4_CANLF NTR domain-containing protein OS=Canis lupus familiaris OX=9615 GN=LOC102154527 PE=3 SV=2	0.1	0.01	116.6	14493173.9	1	1	0	8.6	221	39505
>tr|A0A5F4CUE8|A0A5F4CUE8_CANLF Senataxin OS=Canis lupus familiaris OX=9615 GN=SETX PE=4 SV=1	0.27	0.25	123.7	13270568.7	2	1	0	0.26	2645	1796
>tr|J9P3R7|J9P3R7_CANLF SCO-spondin OS=Canis lupus familiaris OX=9615 GN=SSPO PE=3 SV=2	0.1	0	126.1	11392323.2	5	1	1	0.23	5112	24898
>tr|F1PCU5|F1PCU5_CANLF Methyltransf_11 domain-containing protein OS=Canis lupus familiaris OX=9615 GN=LOC480074 PE=4 SV=3	1.26	1.26	126.2	15398710	1	1	0	2.86	630	1484

>tr|J9NYP3|J9NYP3_CANLF THAP domain containing 7 OS=Canis lupus familiaris OX=9615 GN=THAP7 PE=4 SV=2	0.12	0.01	126.2	130894182.5	44	2	1	2.43	411	5194
>tr|F1PB65|F1PB65_CANLF RAD54 like 2 OS=Canis lupus familiaris OX=9615 GN=RAD54L2 PE=3 SV=2	0.78	0.78	130.3	21151961.3	1	1	0	0.55	1467	11575
>tr|A0A5F4DKE8|A0A5F4DKE8_CANLF Suppression of tumorigenicity 7 like OS=Canis lupus familiaris OX=9615 GN=ST7L PE=3 SV=1	0.18	0.18	133.4	10820650.2	1	1	0	0.78	644	5269
>sp|Q9TU69|GHR_CANLF Growth hormone receptor OS=Canis lupus familiaris OX=9615 GN=GHR PE=2 SV=1	0.1	0.04	138.3	31930926	2	1	0	1.72	638	541
>tr|A0A5F4C0U6|A0A5F4C0U6_CANLF MLLT1 super elongation complex subunit OS=Canis lupus familiaris OX=9615 GN=MLLT1 PE=4 SV=1	0.1	0	138.5	313799140.8	34	2	0	1.17	597	1602
>tr|F1PPP9|F1PPP9_CANLF Family with sequence similarity 135 member A OS=Canis lupus familiaris OX=9615 GN=FAM135A PE=3 SV=3	0.1	0.07	145.1	27171210.3	9	1	0	1.22	1399	6815
>sp|P25473|CLUS_CANLF Clusterin OS=Canis lupus familiaris OX=9615 GN=CLU PE=2 SV=1	0.1	0	145.3	25224754	3	1	0	2.25	445	725
>tr|A0A5F4CLI1|A0A5F4CLI1_CANLF Histone deacetylase 6 OS=Canis lupus familiaris OX=9615 GN=HDAC6 PE=4 SV=1	0.1	0	149.3	32754040.1	2	1	0	0.43	1175	4057
>tr|A0A5F4CGE0|A0A5F4CGE0_CANLF Ubiquitin protein ligase E3C OS=Canis lupus familiaris OX=9615 GN=UBE3C PE=4 SV=1	0.11	0.08	152.1	49667773.2	3	1	0	0.97	1238	9018
>tr|A0A5F4DCA4|A0A5F4DCA4_CANLF Reverse transcriptase domain-containing protein OS=Canis lupus familiaris OX=9615 PE=4 SV=1	1.22	1.22	155.5	7526476.4	1	1	0	0.31	978	860
>tr|F1P8J6|F1P8J6_CANLF RNA helicase OS=Canis lupus familiaris OX=9615 GN=DDX55 PE=3 SV=3	0.1	0.01	155.8	66489374.3	4	1	0	1.58	568	8934

>tr|A0A5F4C7P9|A0A5F4C7P9_CANLF Beta-2-microglobulin OS=Canis lupus familiaris OX=9615 GN=B2M PE=4 SV=1	0.93	0.93	156.6	14403347.3	1	1	0	12.15	107	10611
>tr|J9NTK2|J9NTK2_CANLF J domain-containing protein OS=Canis lupus familiaris OX=9615 GN=DNAJC12 PE=4 SV=2	0.1	0	164.1	100959245.2	6	1	0	4.72	106	2310
>tr|A0A5F4CS87|A0A5F4CS87_CANLF STE20 related adaptor alpha OS=Canis lupus familiaris OX=9615 GN=STRADA PE=4 SV=1	0.41	0.41	166.9	17648561.6	1	1	0	2.08	336	14044
>tr|J9NYC7|J9NYC7_CANLF Dynein axonemal heavy chain 12 OS=Canis lupus familiaris OX=9615 GN=DNAH12 PE=3 SV=1	0.92	0.92	168.1	18704884.2	1	1	0	0.33	3960	15992
>tr|J9P6I3|J9P6I3_CANLF Chloride intracellular channel protein OS=Canis lupus familiaris OX=9615 GN=CLIC2 PE=3 SV=1	0.1	0	170.6	38559607.2	2	1	0	2.93	239	40482
>tr|F1PBU5|F1PBU5_CANLF Non-specific serine/threonine protein kinase OS=Canis lupus familiaris OX=9615 GN=SMG1 PE=3 SV=3	0.83	0.83	173.3	8268862.3	1	1	0	0.08	3634	6898
>tr|E2RN16|E2RN16_CANLF Mitogen-activated protein kinase kinase kinase 2 OS=Canis lupus familiaris OX=9615 GN=MAP3K2 PE=4 SV=2	0.2	0.2	173.5	15468692.3	1	1	0	0.97	620	34325
>tr|A0A5F4CMF9|A0A5F4CMF9_CANLF Cilia and flagella associated protein 65 OS=Canis lupus familiaris OX=9615 GN=CFAP65 PE=4 SV=1	1.07	1.08	174.6	8276036.7	1	1	0	0.17	1789	4175
>tr|A0A5F4BZW4|A0A5F4BZW4_CANLF Malonyl-CoA decarboxylase OS=Canis lupus familiaris OX=9615 GN=MLYCD PE=4 SV=1	0.1	0.02	174.6	31434672.5	2	1	0	1.3	461	4809
>tr|J9NS29|J9NS29_CANLF Cystatin domain-containing protein OS=Canis lupus familiaris OX=9615 GN=LOC607874 PE=4 SV=2	0.79	0.75	178.8	43209619.1	3	1	0	6.39	313	30016

>tr|A0A5F4CCE2|A0A5F4CCE2_CANLF Pappalysin 2 OS=Canis lupus familiaris OX=9615 GN=PAPPA2 PE=3 SV=1	0.27	0.27	180	16348970.5	1	1	0	0.81	1722	20443
>tr|F1PJY1|F1PJY1_CANLF Mannosyl-glycoprotein endo-beta-N-acetylglucosaminidase OS=Canis lupus familiaris OX=9615 GN=ENGASE PE=3 SV=3	0.1	0	183	11493761.4	5	2	2	1.74	690	32761
>sp|Q6AW47|EST5A_CANLF Carboxylesterase 5A OS=Canis lupus familiaris OX=9615 GN=CES5A PE=2 SV=1	0.86	0.86	186.4	16101669.9	1	1	0	0.87	575	629
>tr|A0A5K1V0D8|A0A5K1V0D8_CANLF Sulfatase 2 OS=Canis lupus familiaris OX=9615 GN=SULF2 PE=3 SV=1	0.67	0.61	201.3	26246759.4	4	1	0	0.35	859	1192
>tr|E2R6E0|E2R6E0_CANLF Lipocln_cytosolic_FA-bd_dom domain-containing protein OS=Canis lupus familiaris OX=9615 GN=LCNL1 PE=3 SV=2	3.49	2.19	207.8	122698749.5	10	4	0	11.37	299	1932
>tr|A0A5F4C730|A0A5F4C730_CANLF Semaphorin 4D OS=Canis lupus familiaris OX=9615 GN=SEMA4D PE=3 SV=1	0.46	0.28	217.7	86184259.5	10	1	0	0.28	1067	1802
>sp|O46607|GPX5_CANLF Epididymal secretory glutathione peroxidase OS=Canis lupus familiaris OX=9615 GN=GPX5 PE=2 SV=1	0.95	0.87	218.7	144258937.3	6	2	0	8.6	221	564
>tr|E2RN56|E2RN56_CANLF Zinc finger CCCH-type containing 13 OS=Canis lupus familiaris OX=9615 GN=ZC3H13 PE=4 SV=3	1.22	1.22	220	7605712.3	1	1	0	0.24	1660	1446
>tr|A0A5F4D7Y5|A0A5F4D7Y5_CANLF Pleckstrin homology, MyTH4 and FERM domain containing H1 OS=Canis lupus familiaris OX=9615 GN=PLEKHH1 PE=4 SV=1	0.82	0.78	234.9	34468734.1	3	1	0	0.3	1342	5979

>tr|A0A5F4D6L9|A0A5F4D6L9_CANLF Sacsin molecular chaperone OS=Canis lupus familiaris OX=9615 GN=SACS PE=4 SV=1	0.84	0.78	236	37550451.5	5	2	0	0.18	4500	1444
>tr|A0A5F4D7J3|A0A5F4D7J3_CANLF Non-specific serine/threonine protein kinase OS=Canis lupus familiaris OX=9615 GN=CDC42BPA PE=3 SV=1	2.57	2.55	250.2	17414356.9	2	1	0	0.22	1794	1069
>sp|Q28895|NPC2_CANLF NPC intracellular cholesterol transporter 2 OS=Canis lupus familiaris OX=9615 GN=NPC2 PE=2 SV=1	8.32	3.38	270.3	121461626.9	15	3	0	30.2	149	153
>tr|A0A5F4CCD0|A0A5F4CCD0_CANLF Cysteine rich secretory protein 2 OS=Canis lupus familiaris OX=9615 GN=CRISP2 PE=3 SV=1	1.47	1.37	285.3	78862207.4	7	1	0	2.25	311	11017
>tr|Q9XSV4|Q9XSV4_CANLF CE10 protein OS=Canis lupus familiaris OX=9615 GN=ce10 PE=2 SV=1	5.15	3.09	306.7	138867227.7	28	2	0	9.09	110	41542
>sp|Q9XS65|PTGDS_CANLF Prostaglandin-H2 D-isomerase OS=Canis lupus familiaris OX=9615 GN=PTGDS PE=2 SV=1	2.71	1.83	322.1	123286338.8	11	2	1	10.47	191	165
>tr|A0A5F4BVF3|A0A5F4BVF3_CANLF Lactotransferrin OS=Canis lupus familiaris OX=9615 GN=LTF PE=3 SV=1	8.01	2.43	352.2	759899559.8	60	10	0	13.9	626	32850
>sp|O18840|ACTB_CANLF Actin, cytoplasmic 1 OS=Canis lupus familiaris OX=9615 GN=ACTB PE=2 SV=3	4.31	2.86	356.4	386319672.7	26	3	0	5.6	375	642
>tr|E2RG75|E2RG75_CANLF Inactive ribonuclease-like protein 9 OS=Canis lupus familiaris OX=9615 GN=RNASE9 PE=3 SV=2	0.92	0.88	399.9	7883394.3	3	1	1	4.55	198	41734
>tr|A0A5F4D304|A0A5F4D304_CANLF Ring finger protein 112 OS=Canis lupus familiaris OX=9615 GN=RNF112 PE=3 SV=1	0.23	0.23	37.2	426238.2	1	1	0	1.49	871	5762

>tr|A0A5F4C4Q5|A0A5F4C4Q5_CANLF ST6 N-acetylgalactosaminide alpha-2,6-sialyltransferase 1 OS=Canis lupus familiaris OX=9615 GN=ST6GALNAC1 PE=3 SV=1	0.29	0.29	63.4	15026002.7	1	1	0	1.76	683	1306
>tr|F1PIP2|F1PIP2_CANLF Guanylate cyclase OS=Canis lupus familiaris OX=9615 PE=3 SV=3	0.25	0.25	83.5	17641837.7	1	1	0	0.83	1085	12638
>tr|J9P2K4|J9P2K4_CANLF Transmembrane protein 117 OS=Canis lupus familiaris OX=9615 GN=TMEM117 PE=4 SV=2	0.25	0.25	86.9	14257134.3	1	1	0	2.56	508	14687
>tr|F1P6L3|F1P6L3_CANLF Junctional cadherin 5 associated OS=Canis lupus familiaris OX=9615 GN=JCAD PE=4 SV=2	0.51	0.51	87.6	335206.3	1	1	1	0.66	1364	44204
>tr|F1PB88|F1PB88_CANLF Nik related kinase OS=Canis lupus familiaris OX=9615 GN=NRK PE=4 SV=3	0.92	0.93	93	3617672	1	1	0	0.46	1522	3609
>tr|A0A5F4DKY4|A0A5F4DKY4_CANLF Dehydrogenase E1 and transketolase domain containing 1 OS=Canis lupus familiaris OX=9615 GN=DHTKD1 PE=3 SV=1	0.1	0.05	104.8	51440914.1	3	1	0	2.4	834	5545
>sp|Q8WMX5|S15A1_CANLF Solute carrier family 15 member 1 OS=Canis lupus familiaris OX=9615 GN=SLC15A1 PE=2 SV=2	1.31	1.26	105.2	32137387.6	3	2	1	1.55	708	5
>sp|Q2PQH8|GDE_CANLF Glycogen debranching enzyme OS=Canis lupus familiaris OX=9615 GN=AGL PE=2 SV=1	2.49	2.28	112.9	79288050	5	2	0	0.26	1533	23
>tr|E2RHG5|E2RHG5_CANLF Nudix hydrolase 3 OS=Canis lupus familiaris OX=9615 GN=NUDT3 PE=4 SV=1	0.3	0.28	113.1	8358541.2	2	1	0	6.4	172	21894
>tr|J9P2T7|J9P2T7_CANLF 26S proteasome non-ATPase regulatory subunit 5 OS=Canis lupus familiaris OX=9615 PE=4 SV=1	1.54	1.54	116	5896750.8	1	1	0	1.95	461	23870

>tr|A0A5F4DHW1|A0A5F4DHW1_CANLF Pappalysin 2 OS=Canis lupus familiaris OX=9615 GN=PAPPA2 PE=4 SV=1	0.56	0.28	122.8	15694497.4	2	2	0	1.79	1680	22479
>tr|J9P432|J9P432_CANLF Glutamine--fructose-6-phosphate transaminase (isomerizing) OS=Canis lupus familiaris OX=9615 GN=GFPT1 PE=4 SV=2	0.34	0.28	126.8	18852000.2	4	1	0	1.18	677	7191
>tr|A0A5F4CQL9|A0A5F4CQL9_CANLF ATP-dependent RNA helicase DDX1 OS=Canis lupus familiaris OX=9615 GN=DDX1 PE=3 SV=1	0.21	0.16	129	64002380.6	4	1	0	1.15	784	7666
>tr|F1PLT8|F1PLT8_CANLF Sulfhydryl oxidase OS=Canis lupus familiaris OX=9615 GN=QSOX1 PE=3 SV=3	1.82	1.74	132.7	42812978.9	5	1	0	3.35	568	33056
>sp|Q5I2M8|TLR9_CANLF Toll-like receptor 9 OS=Canis lupus familiaris OX=9615 GN=TLR9 PE=2 SV=1	0.23	0.21	133.7	8030072	2	1	0	1.16	1032	382
>tr|A0A5F4DCZ9|A0A5F4DCZ9_CANLF Kinase D interacting substrate 220 OS=Canis lupus familiaris OX=9615 GN=KIDINS220 PE=4 SV=1	0.1	0.08	140.5	36026128.8	2	1	0	0.6	1678	1923
>tr|A0A5F4D967|A0A5F4D967_CANLF GLI family zinc finger 1 OS=Canis lupus familiaris OX=9615 GN=GLI1 PE=3 SV=1	0.1	0.03	140.8	13768232.8	4	1	0	0.7	1139	11817
>sp|P48831|ZP3_CANLF Zona pellucida sperm-binding protein 3 OS=Canis lupus familiaris OX=9615 GN=ZP3 PE=2 SV=1	0.3	0.28	142.7	27165280.4	2	1	0	0.7	426	21
>tr|E2QWD0|E2QWD0_CANLF CAP-Gly domain containing linker protein 1 OS=Canis lupus familiaris OX=9615 GN=CLIP1 PE=4 SV=3	0.74	0.74	143.8	16031700.5	1	1	0	0.86	1403	12547
>tr|E2QX33|E2QX33_CANLF Coiled-coil and C2 domain containing 1A OS=Canis lupus familiaris OX=9615 GN=CC2D1A PE=3 SV=1	0.19	0.17	144.5	11795640.6	2	1	1	0.84	951	10961

>tr|A0A5F4C9V7|A0A5F4C9V7_CANLF Roundabout guidance receptor 3 OS=Canis lupus familiaris OX=9615 GN=ROBO3 PE=4 SV=1	0.31	0.31	144.5	7349836.4	1	1	0	0.63	1421	3563
>tr|A0A5F4D8I6|A0A5F4D8I6_CANLF Phospholipase A2 receptor 1 OS=Canis lupus familiaris OX=9615 GN=PLA2R1 PE=4 SV=1	0.71	0.62	146	21266479.1	6	1	0	0.5	1394	6796
>tr|A0A5F4D952|A0A5F4D952_CANLF FAT atypical cadherin 1 OS=Canis lupus familiaris OX=9615 GN=FAT1 PE=4 SV=1	0.29	0.3	150.9	13213103.5	1	1	0	0.26	4614	14224
>sp|Q6F3J0|NFKB1_CANLF Nuclear factor NF-kappa-B p105 subunit OS=Canis lupus familiaris OX=9615 GN=NFKB1 PE=2 SV=2	0.13	0.11	150.9	26230930	2	1	0	0.31	972	124
>tr|F1PJY1|F1PJY1_CANLF Mannosyl-glycoprotein endo-beta-N-acetylglucosaminidase OS=Canis lupus familiaris OX=9615 GN=ENGASE PE=3 SV=3	1.35	1.16	161.9	47342666.3	9	2	1	1.74	690	32761
>tr|A0A5F4C7E7|A0A5F4C7E7_CANLF 3-beta-hydroxysterol Delta (14)-reductase OS=Canis lupus familiaris OX=9615 GN=LBR PE=3 SV=1	0.1	0.05	163.2	41982111.7	3	1	0	1.04	576	1034
>tr|A0A5F4C2A7|A0A5F4C2A7_CANLF SECIS binding protein 2 OS=Canis lupus familiaris OX=9615 GN=SECISBP2 PE=4 SV=1	0.34	0.34	163.5	2081362.1	1	1	0	1.39	863	11966
>tr|A0A5F4CS87|A0A5F4CS87_CANLF STE20 related adaptor alpha OS=Canis lupus familiaris OX=9615 GN=STRADA PE=4 SV=1	0.23	0.23	165.7	15426344.8	1	1	0	2.08	336	14044
>tr|F1PBU5|F1PBU5_CANLF Non-specific serine/threonine protein kinase OS=Canis lupus familiaris OX=9615 GN=SMG1 PE=3 SV=3	0.68	0.33	172	229564678.8	17	2	0	0.17	3634	6898
>tr|F1PQC9|F1PQC9_CANLF Dynein regulatory complex protein 10 OS=Canis lupus familiaris OX=9615 GN=IQCD PE=3 SV=3	0.1	0.1	183.6	14574910.7	1	1	0	0.89	451	1682

>tr|A0A5F4D0B3|A0A5F4D0B3_CANLF Bromodomain containing 1 OS=Canis lupus familiaris OX=9615 GN=BRD1 PE=4 SV=1	0.1	0.05	184.7	70399871.5	4	1	0	0.81	1112	1580
>tr|A0A5F4BQW4|A0A5F4BQW4_CANLF Zinc finger protein 654 OS=Canis lupus familiaris OX=9615 GN=ZNF654 PE=4 SV=1	0.65	0.35	184.7	215177541.3	16	1	1	1.03	1170	6910
>tr|A0A5F4CNP4|A0A5F4CNP4_CANLF ADP ribosylation factor GTPase activating protein 3 OS=Canis lupus familiaris OX=9615 GN=ARFGAP3 PE=4 SV=1	0.29	0.27	186	17577143.1	2	1	0	1.12	714	964
>tr|E2R6E0|E2R6E0_CANLF Lipocln_cytosolic_FA-bd_dom domain-containing protein OS=Canis lupus familiaris OX=9615 GN=LCNL1 PE=3 SV=2	3.2	3.14	187.2	48134410.4	4	1	0	3.01	299	1932
>tr|A0A5F4D7Y5|A0A5F4D7Y5_CANLF Pleckstrin homology, MyTH4 and FERM domain containing H1 OS=Canis lupus familiaris OX=9615 GN=PLEKHH1 PE=4 SV=1	0.25	0.23	194	25247667.4	2	1	0	0.3	1342	5979
>tr|F1Q1J0|F1Q1J0_CANLF DIX domain containing 1 OS=Canis lupus familiaris OX=9615 GN=DIXDC1 PE=4 SV=2	0.23	0.23	207.6	6680988.4	1	1	0	0.73	683	3850
>tr|F1PI09|F1PI09_CANLF Aldehyde oxidase OS=Canis lupus familiaris OX=9615 GN=AOX2 PE=3 SV=3	1.41	1.05	208.3	112062641.8	19	1	0	0.67	1347	21650
>sp|Q9GL25|ESPB1_CANLF Epididymal sperm-binding protein 1 OS=Canis lupus familiaris OX=9615 GN=ELSPBP1 PE=1 SV=1	2.84	1.65	213.6	96733442.7	7	2	0	11.43	245	36
>sp|P25473|CLUS_CANLF Clusterin OS=Canis lupus familiaris OX=9615 GN=CLU PE=2 SV=1	0.29	0.27	235	39800627	2	1	0	0.9	445	725
>tr|A0A5F4CCD0|A0A5F4CCD0_CANLF Cysteine rich secretory protein 2 OS=Canis lupus familiaris OX=9615 GN=CRISP2 PE=3 SV=1	1.56	1.54	244	27758081.1	2	1	0	2.25	311	11017

>sp|Q5JZQ9|CLN5_CANLF Ceroid-lipofuscinosis neuronal protein 5 OS=Canis lupus familiaris OX=9615 GN=CLN5 PE=2 SV=1	0.9	0.86	254.1	49880822.2	3	1	0	1.14	350	651
>tr|E2RPK8|E2RPK8_CANLF Phosphatidylethanolamine binding protein 4 OS=Canis lupus familiaris OX=9615 GN=PEBP4 PE=3 SV=2	3.92	3.25	270.5	68457823.8	5	2	0	7.29	247	4725
>tr|E2RCT1|E2RCT1_CANLF WAP domain-containing protein OS=Canis lupus familiaris OX=9615 PE=4 SV=2	1.97	1.75	278.4	109486759	7	2	0	9.48	116	21717
>sp|O18840|ACTB_CANLF Actin, cytoplasmic 1 OS=Canis lupus familiaris OX=9615 GN=ACTB PE=2 SV=3	8.05	3.92	296.4	390446920.9	21	4	0	13.07	375	642
>tr|Q9XSV4|Q9XSV4_CANLF CE10 protein OS=Canis lupus familiaris OX=9615 GN=ce10 PE=2 SV=1	3.82	3.16	309.9	197948110.7	37	2	0	9.09	110	41542
>tr|F1PGF9|F1PGF9_CANLF Rho guanine nucleotide exchange factor 26 OS=Canis lupus familiaris OX=9615 GN=ARHGEF26 PE=4 SV=3	2.09	2.05	312.3	95855749.2	4	2	1	2.36	594	22876
>tr|F1PR54|F1PR54_CANLF Lactotransferrin OS=Canis lupus familiaris OX=9615 GN=LTF PE=3 SV=1	17.9	4.31	326.6	1652321844	108	12	3	15.68	708	40436
>tr|E2RG75|E2RG75_CANLF Inactive ribonuclease-like protein 9 OS=Canis lupus familiaris OX=9615 GN=RNASE9 PE=3 SV=2	3.63	3.52	331.6	57884252.8	8	2	1	8.08	198	41734
>sp|Q9XS65|PTGDS_CANLF Prostaglandin-H2 D-isomerase OS=Canis lupus familiaris OX=9615 GN=PTGDS PE=2 SV=1	7.49	3.22	345.7	780863794.3	40	4	1	13.09	191	165
>sp|P49822|ALBU_CANLF Albumin OS=Canis lupus familiaris OX=9615 GN=ALB PE=1 SV=3	15.98	4.19	403.1	566233765.5	36	8	0	16.61	608	490
>sp|Q28894|WFDC2_CANLF WAP four-disulfide core domain protein 2 OS=Canis lupus familiaris OX=9615 GN=WFDC2 PE=2 SV=1	4.54	4.38	467.2	50655447.3	9	1	0	6.45	124	53

>sp|Q28895|NPC2_CANLF NPC intracellular cholesterol transporter 2 OS=Canis lupus familiaris OX=9615 GN=NPC2 PE=2 SV=1	11.15	4.65	591.2	305690403.5	52	3	0	30.2	149	153
>tr|A0A5F4C9T7|A0A5F4C9T7_CANLF Telomerase associated protein 1 OS=Canis lupus familiaris OX=9615 GN=TEP1 PE=4 SV=1	0.1	0	32.6	50450702.5	3	1	0	0.16	2507	891
>tr|E2RIV7|E2RIV7_CANLF Syntrophin alpha 1 OS=Canis lupus familiaris OX=9615 GN=SNTA1 PE=3 SV=3	0.1	0	33.9	2913593.7	2	1	1	1.03	486	34454
>sp|A2IBY8|MIP_CANLF Lens fiber major intrinsic protein OS=Canis lupus familiaris OX=9615 GN=MIP PE=2 SV=1	0.1	0	34.4	1437915.6	1	1	0	1.9	263	112
>tr|F1PCZ0|F1PCZ0_CANLF Septin OS=Canis lupus familiaris OX=9615 GN=SEPTIN5 PE=3 SV=2	0.65	0.65	38	486065.2	1	1	0	4.23	378	3212
>tr|E2RA00|E2RA00_CANLF Leucine rich repeat containing 8 VRAC subunit D OS=Canis lupus familiaris OX=9615 GN=LRRC8D PE=3 SV=1	0.1	0	41	20869413.5	1	1	1	1.98	858	42697
>tr|E2R868|E2R868_CANLF [histone H4]-N-methyl-L-lysine20 N-methyltransferase KMT5B OS=Canis lupus familiaris OX=9615 GN=KMT5B PE=4 SV=3	0.1	0	42.5	2848313.2	2	1	0	0.56	885	7704
>tr|E2RQX2|E2RQX2_CANLF Ubiquitin specific peptidase 53 OS=Canis lupus familiaris OX=9615 GN=USP53 PE=4 SV=2	0.65	0.65	43.5	1688846.7	1	1	1	1.35	1112	11373
>tr|J9P3H8|J9P3H8_CANLF ATM interactor OS=Canis lupus familiaris OX=9615 GN=ATMIN PE=4 SV=2	0.2	0.1	44	28658261	11	1	0	0.58	863	882
>tr|A0A5F4C0S7|A0A5F4C0S7_CANLF HEAT repeat containing 5A OS=Canis lupus familiaris OX=9615 GN=HEATR5A PE=3 SV=1	0.1	0	51.6	4620329.6	3	1	0	0.25	1995	1753

>tr|J9P432|J9P432_CANLF Glutamine--fructose-6-phosphate transaminase (isomerizing) OS=Canis lupus familiaris OX=9615 GN=GFPT1 PE=4 SV=2	0.23	0.23	53.5	6457411.5	1	1	0	1.18	677	7191
>tr|F1PGK9|F1PGK9_CANLF ADAM metallopeptidase with thrombospondin type 1 motif 5 OS=Canis lupus familiaris OX=9615 GN=ADAMTS5 PE=4 SV=3	0.29	0.02	62.8	71960179.4	31	1	0	0.59	845	11956
>tr|F1PJP1|F1PJP1_CANLF Dynein axonemal heavy chain 11 OS=Canis lupus familiaris OX=9615 GN=DNAH11 PE=3 SV=3	0.65	0.65	62.9	1056658.7	1	1	0	0.35	4519	42705
>tr|A0A5F4D1D2|A0A5F4D1D2_CANLF Protein-tyrosine-phosphatase OS=Canis lupus familiaris OX=9615 GN=PTPRD PE=3 SV=1	2.53	2.52	63	33485086.8	2	1	0	0.6	1828	2208
>tr|A0A5F4CSE7|A0A5F4CSE7_CANLF TYR_PHOSPHATASE_2 domain-containing protein OS=Canis lupus familiaris OX=9615 GN=PTP4A1 PE=4 SV=1	0.1	0	63.4	24837297.8	2	1	1	5.2	173	25182
>tr|E2R186|E2R186_CANLF Fibroblast growth factor receptor OS=Canis lupus familiaris OX=9615 GN=FGFR1 PE=3 SV=3	0.1	0.02	74.7	17701554.6	10	1	0	0.59	853	9797
>tr|F6XBJ5|F6XBJ5_CANLF Integrator complex subunit 1 OS=Canis lupus familiaris OX=9615 GN=INTS1 PE=4 SV=1	0.68	0.66	75.3	2672468.8	2	1	0	0.73	2188	9613
>tr|A0A5F4D6Q9|A0A5F4D6Q9_CANLF Sialic acid binding Ig like lectin 1 OS=Canis lupus familiaris OX=9615 GN=SIGLEC1 PE=4 SV=1	0.38	0.38	76.9	1673862.5	1	1	1	0.99	1719	15622
>tr|A0A5F4CDG4|A0A5F4CDG4_CANLF Protein phosphatase 1 regulatory subunit 32 OS=Canis lupus familiaris OX=9615 GN=PPP1R32 PE=4 SV=1	0.51	0.51	82.6	19036886	1	1	1	2.56	429	3606

>tr|A0A5F4CPE1|A0A5F4CPE1_CANLF Major facilitator superfamily domain containing 14B OS=Canis lupus familiaris OX=9615 GN=MFSD14B PE=4 SV=1	0.1	0	83.6	38928765.7	2	1	0	3.71	485	28321
>sp|P62286|ASPM_CANLF Abnormal spindle-like microcephaly-associated protein homolog OS=Canis lupus familiaris OX=9615 GN=ASPM PE=2 SV=2	0.25	0.15	86.6	605260377.9	67	3	0	0.29	3469	677
>tr|E2RE16|E2RE16_CANLF Non-specific serine/threonine protein kinase OS=Canis lupus familiaris OX=9615 GN=PAK4 PE=4 SV=1	0.61	0.02	86.7	112965242.4	57	1	0	0.84	592	12735
>tr|F1PPP9|F1PPP9_CANLF Family with sequence similarity 135 member A OS=Canis lupus familiaris OX=9615 GN=FAM135A PE=3 SV=3	0.14	0.14	93.4	1627559.1	1	1	0	1.22	1399	6815
>tr|E2R4U2|E2R4U2_CANLF Proline rich 35 OS=Canis lupus familiaris OX=9615 GN=PRR35 PE=4 SV=2	0.29	0.3	93.9	158230.7	1	1	0	1.49	536	44008
>tr|A0A5F4D9Z7|A0A5F4D9Z7_CANLF Zinc finger FYVE-type containing 19 OS=Canis lupus familiaris OX=9615 GN=ZFYVE19 PE=4 SV=1	0.12	0.12	96.8	40635378.9	2	1	0	1.53	392	13875
>tr|A0A5F4CAH2|A0A5F4CAH2_CANLF RNA polymerase II subunit A C-terminal domain phosphatase OS=Canis lupus familiaris OX=9615 GN=CTDP1 PE=4 SV=1	0.19	0.19	101.5	10684469.1	1	1	0	0.74	945	14396
>tr|J9NTK2|J9NTK2_CANLF J domain-containing protein OS=Canis lupus familiaris OX=9615 GN=DNAJC12 PE=4 SV=2	0.1	0	104.3	33689209.6	2	1	0	4.72	106	2310
>tr|F1P6D8|F1P6D8_CANLF Dynein axonemal heavy chain 5 OS=Canis lupus familiaris OX=9615 GN=DNAH5 PE=3 SV=3	0.1	0	105.4	16532748.4	1	1	0	0.19	4620	37739

>tr|A0A5F4CIJ4|A0A5F4CIJ4_CANLF Transmembrane protein 63A OS=Canis lupus familiaris OX=9615 GN=TMEM63A PE=3 SV=1	0.4	0.4	115.4	264293	1	1	0	0.76	789	13092
>tr|A0A5F4CF57|A0A5F4CF57_CANLF WD repeat domain 90 OS=Canis lupus familiaris OX=9615 GN=WDR90 PE=4 SV=1	0.1	0.02	116.4	13631870.4	1	1	0	0.26	2322	3876
>tr|A0A5F4DIL6|A0A5F4DIL6_CANLF Dpy-19 like C-mannosyltransferase 3 OS=Canis lupus familiaris OX=9615 GN=DPY19L3 PE=3 SV=1	0.3	0.28	117	1796079.9	2	1	1	1.75	742	42539
>tr|A0A5F4CS27|A0A5F4CS27_CANLF Abhydrolase domain containing 14B OS=Canis lupus familiaris OX=9615 GN=ABHD14B PE=4 SV=1	0.1	0	126.3	31942504.9	2	1	0	2.56	234	21786
>tr|F1PKS8|F1PKS8_CANLF Anoctamin OS=Canis lupus familiaris OX=9615 GN=VWF PE=3 SV=3	0.12	0.02	133.6	2612261.7	6	1	1	1.61	992	42310
>tr|E2RTH4|E2RTH4_CANLF Dendrin OS=Canis lupus familiaris OX=9615 GN=DDN PE=4 SV=2	0.65	0.65	133.7	1515276.4	1	1	1	2.84	704	20318
>sp|P23685|NAC1_CANLF Sodium/calcium exchanger 1 OS=Canis lupus familiaris OX=9615 GN=SLC8A1 PE=1 SV=1	0.37	0.37	153.6	982205	1	1	0	1.03	970	764
>tr|F1PB65|F1PB65_CANLF RAD54 like 2 OS=Canis lupus familiaris OX=9615 GN=RAD54L2 PE=3 SV=2	0.3	0.28	158.6	35733148.2	2	1	0	0.55	1467	11575
>tr|A0A5F4D7Y5|A0A5F4D7Y5_CANLF Pleckstrin homology, MyTH4 and FERM domain containing H1 OS=Canis lupus familiaris OX=9615 GN=PLEKHH1 PE=4 SV=1	0.54	0.5	180.1	14432697.4	3	1	0	0.3	1342	5979
>tr|A0A5K1V0D8|A0A5K1V0D8_CANLF Sulfatase 2 OS=Canis lupus familiaris OX=9615 GN=SULF2 PE=3 SV=1	0.1	0.02	189.6	64093192.6	11	1	0	0.35	859	1192

>tr|E2R6E0|E2R6E0_CANLF Lipocln_cytosolic_FA-bd_dom domain-containing protein OS=Canis lupus familiaris OX=9615 GN=LCNL1 PE=3 SV=2	2.12	2.12	199.4	7947178.4	1	1	0	3.68	299	1932
>tr|J9NS29|J9NS29_CANLF Cystatin domain-containing protein OS=Canis lupus familiaris OX=9615 GN=LOC607874 PE=4 SV=2	0.46	0.46	202.4	35486277.3	2	1	0	6.39	313	30016
>tr|A0A5F4D6L9|A0A5F4D6L9_CANLF Sacsin molecular chaperone OS=Canis lupus familiaris OX=9615 GN=SACS PE=4 SV=1	0.84	0.82	206.6	9407695.2	2	1	0	0.09	4500	1444
>sp|Q9XS65|PTGDS_CANLF Prostaglandin-H2 D-isomerase OS=Canis lupus familiaris OX=9615 GN=PTGDS PE=2 SV=1	0.49	0.41	222.2	1260896.8	5	1	1	7.33	191	165
>tr|Q9XSV4|Q9XSV4_CANLF CE10 protein OS=Canis lupus familiaris OX=9615 GN=ce10 PE=2 SV=1	3.28	2.61	253.3	117020949.8	19	3	0	14.55	110	41542
>sp|O18840|ACTB_CANLF Actin, cytoplasmic 1 OS=Canis lupus familiaris OX=9615 GN=ACTB PE=2 SV=3	4.71	3.57	281.8	273998734.5	19	2	0	5.33	375	642
>tr|A0A5F4BVF3|A0A5F4BVF3_CANLF Lactotransferrin OS=Canis lupus familiaris OX=9615 GN=LTF PE=3 SV=1	8.24	2.47	289.5	708660872.9	51	8	0	7.99	626	32850
>sp|Q28895|NPC2_CANLF NPC intracellular cholesterol transporter 2 OS=Canis lupus familiaris OX=9615 GN=NPC2 PE=2 SV=1	6.65	6.35	427	12973006	16	1	0	15.44	149	153
>tr|E2RJR2|E2RJR2_CANLF NADH:ubiquinone oxidoreductase subunit A9 OS=Canis lupus familiaris OX=9615 GN=NDUFA9 PE=4 SV=2	0.1	0.02	31.9	14129329.5	3	1	1	5.31	377	3530
>sp|P50996|ATP4A_CANLF Potassium-transporting ATPase alpha chain 1 OS=Canis lupus familiaris OX=9615 GN=ATP4A PE=2 SV=3	0.55	0.55	38.4	5902147.9	1	1	1	2.9	1034	672

>tr|A0A5F4CP99|A0A5F4CP99_CANLF CD109 molecule OS=Canis lupus familiaris OX=9615 GN=CD109 PE=3 SV=1	0.57	0.57	50.6	6058965.5	1	1	0	1.84	1520	4665
>tr|F6XAZ8|F6XAZ8_CANLF Dispatched RND transporter family member 1 OS=Canis lupus familiaris OX=9615 GN=DISP1 PE=4 SV=2	0.1	0.06	57.9	6150747.1	1	1	1	1.01	1485	5621
>sp|Q697L1|TRPV1_CANLF Transient receptor potential cation channel subfamily V member 1 OS=Canis lupus familiaris OX=9615 GN=TRPV1 PE=2 SV=1	0.33	0.31	73.2	3403778.4	2	1	1	0.36	840	297
>tr|A0A5F4CU74|A0A5F4CU74_CANLF FYVE, RhoGEF and PH domain containing 6 OS=Canis lupus familiaris OX=9615 GN=FGD6 PE=4 SV=1	0.1	0.05	74	5395228.4	1	1	1	1.67	1320	14282
>tr|F1PQU5|F1PQU5_CANLF Synaptic vesicle glycoprotein 2C OS=Canis lupus familiaris OX=9615 GN=SV2C PE=3 SV=2	0.6	0.58	75.9	15675333.5	2	1	0	2.76	724	12575
>tr|J9P822|J9P822_CANLF TPR_REGION domain-containing protein OS=Canis lupus familiaris OX=9615 GN=TTC16 PE=4 SV=2	0.58	0.58	83.6	5461242.9	1	1	0	1.73	866	2823
>tr|A0A5F4CXV0|A0A5F4CXV0_CANLF Mahogunin ring finger 1 OS=Canis lupus familiaris OX=9615 GN=MGRN1 PE=4 SV=1	0.31	0.31	84	4399504.7	1	1	0	2.19	549	4641
>sp|P62286|ASPM_CANLF Abnormal spindle-like microcephaly-associated protein homolog OS=Canis lupus familiaris OX=9615 GN=ASPM PE=2 SV=2	1.96	0.14	85.2	287427350.8	90	3	0	0.32	3469	677
>tr|E2RHZ3|E2RHZ3_CANLF DEAH-box helicase 37 OS=Canis lupus familiaris OX=9615 GN=DHX37 PE=4 SV=1	0.4	0.4	90.4	3802195.2	1	1	1	0.52	1149	44834

>tr|F1PFP6|F1PFP6_CANLF Matrix metallopeptidase 12 OS=Canis lupus familiaris OX=9615 GN=MMP12 PE=3 SV=3	0.1	0.02	95.6	6344627.9	1	1	0	2.44	491	24324
>tr|F6XN72|F6XN72_CANLF Leucine rich repeat containing 71 OS=Canis lupus familiaris OX=9615 GN=LRRC71 PE=4 SV=1	0.15	0.15	97.4	4431542.6	1	1	1	1.96	560	34087
>sp|P23685|NAC1_CANLF Sodium/calcium exchanger 1 OS=Canis lupus familiaris OX=9615 GN=SLC8A1 PE=1 SV=1	0.1	0.05	98.6	3254155.3	1	1	0	1.03	970	764
>tr|F1PWR1|F1PWR1_CANLF Insulin like growth factor 2 receptor OS=Canis lupus familiaris OX=9615 GN=IGF2R PE=4 SV=3	0.1	0.03	105.3	4278844.4	1	1	1	0.85	2474	1073
>tr|E2RR09|E2RR09_CANLF Sodium channel protein OS=Canis lupus familiaris OX=9615 GN=SCN4A PE=3 SV=2	0.33	0.29	106.1	20341988.1	3	1	1	0.33	1837	22889
>tr|A0A5F4C4H6|A0A5F4C4H6_CANLF Biliverdin reductase A OS=Canis lupus familiaris OX=9615 GN=BLVRA PE=4 SV=1	0.1	0.07	106.7	11996867.4	2	1	0	3.49	401	15382
>tr|A0A5F4C8H3|A0A5F4C8H3_CANLF Glycerol-3-phosphate acyltransferase 1, mitochondrial OS=Canis lupus familiaris OX=9615 GN=GPAM PE=3 SV=1	0.79	0.76	106.9	6793061.6	2	2	1	2.63	837	11048
>tr|F1PED7|F1PED7_CANLF Poly [ADP-ribose] polymerase OS=Canis lupus familiaris OX=9615 GN=PARP3 PE=4 SV=2	0.59	0.59	115.1	7523536.3	1	1	0	2.07	531	21143
>tr|A0A5F4CKD5|A0A5F4CKD5_CANLF Polypeptide N-acetylgalactosaminyltransferase OS=Canis lupus familiaris OX=9615 GN=GALNT6 PE=3 SV=1	0.69	0.69	115.9	5950260.1	1	1	0	1.71	644	1617
>tr|E2RN65|E2RN65_CANLF Phosphatidylinositol-glycan biosynthesis class W protein OS=Canis lupus familiaris OX=9615 GN=PIGW PE=3 SV=2	0.39	0.37	116.2	5261859.4	2	1	0	1.38	509	21598

>sp|E2RKA8|RL32_CANLF 60S ribosomal protein L32 OS=Canis lupus familiaris OX=9615 GN=RPL32 PE=1 SV=1	3.52	0.59	116.9	555722532.7	133	2	0	4.44	135	275
>sp|F1PRN2|MYO1D_CANLF Unconventional myosin-Id OS=Canis lupus familiaris OX=9615 GN=MYO1D PE=1 SV=2	0.69	0.63	117.8	7978788.7	4	1	0	0.3	1006	763
>tr|E2RRE4|E2RRE4_CANLF Netrin 5 OS=Canis lupus familiaris OX=9615 GN=NTN5 PE=4 SV=3	0.16	0.14	125.3	6676765	2	1	0	1.02	489	13410
>tr|A0A5F4CRH0|A0A5F4CRH0_CANLF Ceruloplasmin OS=Canis lupus familiaris OX=9615 GN=CP PE=3 SV=1	0.36	0.34	128	11920452.2	2	1	0	1.41	1063	2330
>tr|J9NS29|J9NS29_CANLF Cystatin domain-containing protein OS=Canis lupus familiaris OX=9615 GN=LOC607874 PE=4 SV=2	0.35	0.33	146.8	11441647.2	2	1	0	6.39	313	30016
>tr|F1PHA9|F1PHA9_CANLF Motile sperm domain containing 2 OS=Canis lupus familiaris OX=9615 GN=MOSPD2 PE=4 SV=3	0.57	0.57	148.2	4672931.6	1	1	0	0.97	518	10201
>tr|F1PQM0|F1PQM0_CANLF BAH domain and coiled-coil containing 1 OS=Canis lupus familiaris OX=9615 GN=BAHCC1 PE=4 SV=3	0.61	0.57	154	19044310.8	3	1	1	0.43	2565	10908
>tr|A0A5F4BSI9|A0A5F4BSI9_CANLF Centrosomal protein 350 OS=Canis lupus familiaris OX=9615 GN=CEP350 PE=4 SV=1	0.1	0.04	158.9	4672931.6	1	1	0	0.32	3113	15196
>tr|F1Q2F6|F1Q2F6_CANLF 3-hydroxyacyl-[acyl-carrier-protein] dehydratase OS=Canis lupus familiaris OX=9615 GN=FASN PE=4 SV=3	0.8	0.78	173.9	10047512.1	2	1	0	0.4	2478	22936
>sp|P49822|ALBU_CANLF Albumin OS=Canis lupus familiaris OX=9615 GN=ALB PE=1 SV=3	3.82	0.88	276.2	114292000.1	19	5	1	8.39	608	490
>tr|Q9XSV4|Q9XSV4_CANLF CE10 protein OS=Canis lupus familiaris OX=9615 GN=ce10 PE=2 SV=1	3.86	1.63	281.4	38452000.8	16	3	0	14.55	110	41542

>tr|A0A5F4BVF3|A0A5F4BVF3_CANLF Lactotransferrin OS=Canis lupus familiaris OX=9615 GN=LTF PE=3 SV=1	4.84	2.92	299.4	87110930.6	17	3	0	3.99	626	32850
>sp|O18840|ACTB_CANLF Actin, cytoplasmic 1 OS=Canis lupus familiaris OX=9615 GN=ACTB PE=2 SV=3	5.33	3.46	321.2	111249282.4	15	3	0	5.6	375	642
>tr|A0A5F4CPU3|A0A5F4CPU3_CANLF SEC24 homolog D, COPII coat complex component OS=Canis lupus familiaris OX=9615 GN=SEC24D PE=3 SV=1	0.2	0.13	34.3	29986999	10	1	0	0.53	946	2125
>tr|E2R868|E2R868_CANLF [histone H4]-N-methyl-L-lysine20 N-methyltransferase KMT5B OS=Canis lupus familiaris OX=9615 GN=KMT5B PE=4 SV=3	0.25	0.23	35.3	55400080.5	10	3	2	1.81	885	7704
>tr|F1PGK9|F1PGK9_CANLF ADAM metallopeptidase with thrombospondin type 1 motif 5 OS=Canis lupus familiaris OX=9615 GN=ADAMTS5 PE=4 SV=3	0.23	0.02	35.6	65563484.4	32	1	0	0.59	845	11956
>tr|F1PM73|F1PM73_CANLF Palmitoyltransferase OS=Canis lupus familiaris OX=9615 GN=ZDHHC23 PE=3 SV=3	0.19	0.19	36.6	5484916.3	1	1	0	3.98	427	22460
>tr|E2RIH1|E2RIH1_CANLF DOP1 leucine zipper like protein B OS=Canis lupus familiaris OX=9615 GN=DOP1B PE=3 SV=3	0.81	0.56	37.6	30256843	9	2	0	1.42	2259	7751
>tr|A0A5F4CQY7|A0A5F4CQY7_CANLF Neuregulin 2 OS=Canis lupus familiaris OX=9615 GN=NRG2 PE=4 SV=1	0.26	0.02	39.2	35061327.2	19	1	0	0.62	802	13353
>tr|E2RA54|E2RA54_CANLF Bromodomain and WD repeat domain containing 3 OS=Canis lupus familiaris OX=9615 GN=BRWD3 PE=4 SV=3	0.1	0.02	48.2	6352116	2	1	0	0.29	1750	4294
>tr|F1PS80|F1PS80_CANLF Protein phosphatase 4 regulatory subunit 1 OS=Canis lupus familiaris OX=9615 GN=PPP4R1 PE=4 SV=3	0.37	0.36	51.3	10433783.3	3	1	0	0.56	887	2666

>tr|A0A5F4BZ61|A0A5F4BZ61_CANLF G_PROTEIN_RECEP_F1_2 domain-containing protein OS=Canis lupus familiaris OX=9615 GN=OR5D13 PE=4 SV=1	0.57	0.57	54.2	2896904.8	1	1	0	1.7	294	37537
>tr|J9P5T2|J9P5T2_CANLF Non-specific serine/threonine protein kinase OS=Canis lupus familiaris OX=9615 GN=WNK3 PE=4 SV=2	0.1	0.01	55.1	11038696	4	1	0	0.22	2294	5229
>tr|A0A5F4C0S7|A0A5F4C0S7_CANLF HEAT repeat containing 5A OS=Canis lupus familiaris OX=9615 GN=HEATR5A PE=3 SV=1	0.31	0.13	55.2	51558300.2	17	1	0	0.25	1995	1753
>tr|E2RE16|E2RE16_CANLF Non-specific serine/threonine protein kinase OS=Canis lupus familiaris OX=9615 GN=PAK4 PE=4 SV=1	0.18	0.02	57.6	59242568.1	23	1	0	0.84	592	12735
>tr|J9P9K7|J9P9K7_CANLF Glycylpeptide N-tetradecanoyltransferase OS=Canis lupus familiaris OX=9615 GN=NMT2 PE=3 SV=2	0.32	0.32	59	6229770.5	1	1	0	3.16	507	19368
>tr|F1PBJ1|F1PBJ1_CANLF Methylcytosine dioxygenase TET OS=Canis lupus familiaris OX=9615 GN=TET3 PE=3 SV=2	0.1	0.02	66.1	11476539.9	4	1	0	0.28	1795	1529
>tr|F1PPQ1|F1PPQ1_CANLF Schlafen like 1 OS=Canis lupus familiaris OX=9615 GN=SLFNL1 PE=4 SV=3	0.1	0.01	67.4	1816832	1	1	0	2.31	347	24972
>tr|A0A5K1V5T8|A0A5K1V5T8_CANLF Zinc finger and BTB domain containing 49 OS=Canis lupus familiaris OX=9615 GN=ZBTB49 PE=4 SV=1	0.25	0.21	68.4	40135537.3	3	1	0	1.47	543	5803
>tr|A0A5F4D3R3|A0A5F4D3R3_CANLF Mitogen-activated protein kinase kinase kinase 4 OS=Canis lupus familiaris OX=9615 GN=MAP3K4 PE=4 SV=1	0.29	0.29	79.3	1732983.2	1	1	0	0.55	1630	2223
>tr|A0A5F4DKY4|A0A5F4DKY4_CANLF Dehydrogenase E1 and transketolase domain containing 1 OS=Canis lupus familiaris OX=9615 GN=DHTKD1 PE=3 SV=1	0.18	0.14	81	18683652.4	3	1	0	2.4	834	5545

>sp|A2IBY8|MIP_CANLF Lens fiber major intrinsic protein OS=Canis lupus familiaris OX=9615 GN=MIP PE=2 SV=1	0.64	0.55	81	18104535.5	6	1	0	1.9	263	112
>tr|A0A5F4CCD0|A0A5F4CCD0_CANLF Cysteine rich secretory protein 2 OS=Canis lupus familiaris OX=9615 GN=CRISP2 PE=3 SV=1	0.63	0.61	84.3	12651840.7	2	1	0	4.82	311	11017
>tr|E2R5W0|E2R5W0_CANLF Myosin binding protein C2 OS=Canis lupus familiaris OX=9615 GN=MYBPC2 PE=3 SV=2	1.15	1.15	88.5	4231783.1	1	1	0	4.15	265	19744
>tr|A0A5F4BT89|A0A5F4BT89_CANLF Olfactory receptor OS=Canis lupus familiaris OX=9615 GN=OR5W6 PE=3 SV=1	0.1	0.01	91.8	12092954.6	2	1	1	6.95	302	29923
>tr|A0A5F4D9Z7|A0A5F4D9Z7_CANLF Zinc finger FYVE-type containing 19 OS=Canis lupus familiaris OX=9615 GN=ZFYVE19 PE=4 SV=1	0.19	0.17	100.3	14347306.2	2	1	0	1.53	392	13875
>tr|A0A5F4DDV9|A0A5F4DDV9_CANLF Actin alpha 2, smooth muscle OS=Canis lupus familiaris OX=9615 GN=ACTA2 PE=3 SV=1	0.55	0.55	103	7906270.6	1	1	1	10.16	374	9804
>sp|P62286|ASPM_CANLF Abnormal spindle-like microcephaly-associated protein homolog OS=Canis lupus familiaris OX=9615 GN=ASPM PE=2 SV=2	2.34	0.21	103	349492154.5	94	3	0	0.23	3469	677
>tr|F1PPP9|F1PPP9_CANLF Family with sequence similarity 135 member A OS=Canis lupus familiaris OX=9615 GN=FAM135A PE=3 SV=3	0.2	0.18	105.2	1727197.3	2	1	0	1.22	1399	6815
>tr|A0A5F4C7W7|A0A5F4C7W7_CANLF Phosphatidylinositol-4,5-bisphosphate 3-kinase OS=Canis lupus familiaris OX=9615 GN=PIK3CD PE=3 SV=1	0.2	0.2	106.7	10088526.5	2	1	0	1.52	1051	18533

>tr|J9P432|J9P432_CANLF Glutamine--fructose-6-phosphate transaminase (isomerizing) OS=Canis lupus familiaris OX=9615 GN=GFPT1 PE=4 SV=2	0.79	0.73	107.3	7976954	4	1	0	1.18	677	7191
>sp|E2QRY6|NNRE_CANLF NAD(P)H-hydrate epimerase OS=Canis lupus familiaris OX=9615 GN=NAXE PE=3 SV=1	0.19	0.17	109	357197.7	2	1	0	1.74	288	159
>tr|A0A5F4CUE8|A0A5F4CUE8_CANLF Senataxin OS=Canis lupus familiaris OX=9615 GN=SETX PE=4 SV=1	0.39	0.37	110.6	4835244.7	2	1	0	0.26	2645	1796
>tr|F1P9S9|F1P9S9_CANLF MutS homolog 3 OS=Canis lupus familiaris OX=9615 GN=MSH3 PE=3 SV=3	0.22	0.22	118.1	7514485.2	1	1	0	1.04	1058	29932
>tr|A0A5F4C8H3|A0A5F4C8H3_CANLF Glycerol-3-phosphate acyltransferase 1, mitochondrial OS=Canis lupus familiaris OX=9615 GN=GPAM PE=3 SV=1	0.87	0.87	121.5	4596836.8	1	1	0	1.31	837	11048
>tr|E2RSI6|E2RSI6_CANLF Ezrin OS=Canis lupus familiaris OX=9615 GN=EZR PE=4 SV=1	0.61	0.61	131.8	4896880.6	1	1	0	1.19	586	15650
>sp|P23685|NAC1_CANLF Sodium/calcium exchanger 1 OS=Canis lupus familiaris OX=9615 GN=SLC8A1 PE=1 SV=1	0.25	0.21	133.3	18288613.1	3	1	0	1.03	970	764
>tr|A0A5F4BX19|A0A5F4BX19_CANLF Multidrug and toxin extrusion protein OS=Canis lupus familiaris OX=9615 GN=ALDH3A2 PE=3 SV=1	0.56	0.56	137.7	4451339.3	1	1	0	0.61	983	8236
>tr|A0A5F4DFX0|A0A5F4DFX0_CANLF Exportin-T OS=Canis lupus familiaris OX=9615 GN=XPOT PE=3 SV=1	0.26	0.18	141.7	13567998.2	5	1	0	0.75	938	2329
>tr|A0A5F4C5M9|A0A5F4C5M9_CANLF Contactin associated protein 1 OS=Canis lupus familiaris OX=9615 GN=CNTNAP1 PE=3 SV=1	0.81	0.6	154.4	544591.4	4	2	0	0.65	1388	10827

>tr|J9P758|J9P758_CANLF Sorcin OS=Canis lupus familiaris OX=9615 GN=SRI PE=4 SV=2	0.57	0.57	155.5	11097658.5	1	1	0	2.3	348	7242
>sp|O46607|GPX5_CANLF Epididymal secretory glutathione peroxidase OS=Canis lupus familiaris OX=9615 GN=GPX5 PE=2 SV=1	3.59	1.94	157.9	1119106.1	8	3	0	11.76	221	564
>tr|J9NTK2|J9NTK2_CANLF J domain-containing protein OS=Canis lupus familiaris OX=9615 GN=DNAJC12 PE=4 SV=2	0.23	0.21	160.3	13652893.2	2	1	0	4.72	106	2310
>sp|Q28895|NPC2_CANLF NPC intracellular cholesterol transporter 2 OS=Canis lupus familiaris OX=9615 GN=NPC2 PE=2 SV=1	1.35	1.35	162.3	152904.1	1	1	0	6.04	149	153
>tr|J9NS29|J9NS29_CANLF Cystatin domain-containing protein OS=Canis lupus familiaris OX=9615 GN=LOC607874 PE=4 SV=2	0.68	0.68	183.5	5535283.6	1	1	0	6.39	313	30016
>tr|A0A5K1V0D8|A0A5K1V0D8_CANLF Sulfatase 2 OS=Canis lupus familiaris OX=9615 GN=SULF2 PE=3 SV=1	0.66	0.56	184.5	22817089.7	6	1	0	0.35	859	1192
>sp|Q6AW47|EST5A_CANLF Carboxylesterase 5A OS=Canis lupus familiaris OX=9615 GN=CES5A PE=2 SV=1	0.85	0.79	188.9	825726.6	4	1	0	1.22	575	629
>tr|A0A5F4D6L9|A0A5F4D6L9_CANLF Sacsin molecular chaperone OS=Canis lupus familiaris OX=9615 GN=SACS PE=4 SV=1	0.81	0.54	194.6	140131501.7	44	3	0	0.27	4500	1444
>sp|F1PRN2|MYO1D_CANLF Unconventional myosin-Id OS=Canis lupus familiaris OX=9615 GN=MYO1D PE=1 SV=2	0.37	0.21	206.8	24612720.7	9	1	0	0.3	1006	763
>sp|Q9GL25|ESPB1_CANLF Epididymal sperm-binding protein 1 OS=Canis lupus familiaris OX=9615 GN=ELSPBP1 PE=1 SV=1	1.06	1.02	238	613431.8	3	1	0	4.49	245	36

>tr|E2RG75|E2RG75_CANLF Inactive ribonuclease-like protein 9 OS=Canis lupus familiaris OX=9615 GN=RNASE9 PE=3 SV=2	0.79	0.79	238.6	169807	1	1	0	4.55	198	41734
>sp|Q9XS65|PTGDS_CANLF Prostaglandin-H2 D-isomerase OS=Canis lupus familiaris OX=9615 GN=PTGDS PE=2 SV=1	1.62	1.44	252.2	2203885.9	11	2	1	10.47	191	165
>sp|O18840|ACTB_CANLF Actin, cytoplasmic 1 OS=Canis lupus familiaris OX=9615 GN=ACTB PE=2 SV=3	6.39	3.83	322.2	168494180.5	21	5	0	8.27	375	642
>tr|Q9XSV4|Q9XSV4_CANLF CE10 protein OS=Canis lupus familiaris OX=9615 GN=ce10 PE=2 SV=1	7.57	3.06	363.8	74770867.6	31	4	0	23.64	110	41542
>tr|F1PR54|F1PR54_CANLF Lactotransferrin OS=Canis lupus familiaris OX=9615 GN=LTF PE=3 SV=1	18.69	3.07	397.9	294005519.4	75	15	0	18.22	708	40436
