# Supplementary material for: Potential Proteins Associated with Canine Epididymal Sperm Motility
Source: Cells. 2026 Jan 4;15(1):85. doi: 10.3390/cells15010085 (PMC12786130; doi:10.3390/cells15010085)
Supplement: Supplementary file 1 [file cells-15-00085-s001.zip › Supplementary Table S2.pdf]

Supplementary Table S2. Proteins of the cauda epididymal sperm (ES) of dogs (*Canis lupus familiaris*) with poor sperm motility (PSM).

Description	|Log Prob|	Best |Log Prob|	Best score	Total Intensity	# of spectra	# of unique peptides	# of mod peptides	Coverage %	# AA's in protein	Protein DB number
>tr|A0A5F4DHH0|A0A5F4DHH0_CANLF ATP binding cassette subfamily A member 1 OS=Canis lupus familiaris OX=9615 GN=ABCA1 PE=4 SV=1	0.48	0.46	73.2	5472409.6	2	1	0	0.23	2175	3709
>sp|O18840|ACTB_CANLF Actin, cytoplasmic 1 OS=Canis lupus familiaris OX=9615 GN=ACTB PE=2 SV=3	6.01	3.93	316.4	215049910	16	3	0	10.13	375	642
>tr|F1PGK9|F1PGK9_CANLF ADAM metallopeptidase with thrombospondin type 1 motif 5 OS=Canis lupus familiaris OX=9615 GN=ADAMTS5 PE=4 SV=3	0.1	0.06	50.6	5178752.8	3	1	0	0.59	845	11956
>tr|J9NS28|J9NS28_CANLF RBR-type E3 ubiquitin transferase OS=Canis lupus familiaris OX=9615 GN=ANKIB1 PE=4 SV=2	0.45	0.45	71.4	12776431.9	1	1	0	2.33	988	26345
>sp|E2RED8|AP4M1_CANLF AP-4 complex subunit mu-1 OS=Canis lupus familiaris OX=9615 GN=AP4M1 PE=3 SV=2	0.77	0.61	208.8	29709839.5	9	1	0	0.66	452	634
>tr|F1P920|F1P920_CANLF Apoptosis resistant E3 ubiquitin protein ligase 1 OS=Canis lupus familiaris OX=9615 GN=AREL1 PE=4 SV=3	0.1	0	71.9	1167711957	15	1	0	0.88	793	14252
>tr|A0A5F4CNP4|A0A5F4CNP4_CANLF ADP ribosylation factor GTPase activating protein 3 OS=Canis lupus familiaris OX=9615 GN=ARFGAP3 PE=4 SV=1	0.41	0.37	183.2	20553248.6	3	1	0	1.12	714	964
>tr|A0A5F4D3Q2|A0A5F4D3Q2_CANLF Non-specific serine/threonine protein kinase OS=Canis lupus familiaris OX=9615 GN=ATR PE=3 SV=1	0.1	0.03	112.8	2446716.1	1	1	0	0.35	2583	7077
>tr|F1P9I2|F1P9I2_CANLF Cdk5 and Abl enzyme substrate 1 OS=Canis lupus familiaris OX=9615 GN=CABLES1 PE=4 SV=3	0.1	0.03	139.6	31695250.6	2	1	0	0.92	757	13186

>tr|Q9XSV4|Q9XSV4_CANLF CE10 protein OS=Canis lupus familiaris OX=9615 GN=ce10 PE=2 SV=1	3.99	2.29	391.7	122646525	23	2	0	9.09	110	41542
>tr|A0A5F4D9S5|A0A5F4D9S5_CANLF Hyaluronoglucosaminidase OS=Canis lupus familiaris OX=9615 GN=CEMIP PE=3 SV=1	1.79	1.77	182.5	14236079.5	2	1	0	0.24	1684	9775
>tr|A0A5F4CCD0|A0A5F4CCD0_CANLF Cysteine rich secretory protein 2 OS=Canis lupus familiaris OX=9615 GN=CRISP2 PE=3 SV=1	1.54	1.48	112.2	21777312	4	1	0	4.82	311	11017
>tr|A0A5F4CGB9|A0A5F4CGB9_CANLF Decapping mRNA 1B OS=Canis lupus familiaris OX=9615 GN=DCP1B PE=3 SV=1	0.1	0.03	74.5	13938089.4	4	1	0	0.36	1115	990
>tr|F1P8J6|F1P8J6_CANLF RNA helicase OS=Canis lupus familiaris OX=9615 GN=DDX55 PE=3 SV=3	0.1	0.06	116	17441535	1	1	0	1.58	568	8934
>tr|A0A5F4C887|A0A5F4C887_CANLF O-acyltransferase OS=Canis lupus familiaris OX=9615 GN=DGAT1 PE=3 SV=1	0.1	0.02	51.2	26301544.2	2	1	0	1.93	623	22414
>sp|P01002|IPSG_CANLF Double-headed protease inhibitor, submandibular gland OS=Canis lupus familiaris OX=9615 PE=1 SV=1	1.05	1.03	209.1	19708370.2	2	1	0	7.83	115	434
>tr|E2RQN7|E2RQN7_CANLF RNA helicase OS=Canis lupus familiaris OX=9615 GN=DHX35 PE=4 SV=3	0.1	0.02	37.3	57891199.6	4	1	0	2.95	679	32613
>tr|J9NTK2|J9NTK2_CANLF J domain-containing protein OS=Canis lupus familiaris OX=9615 GN=DNAJC12 PE=4 SV=2	0.1	0.03	121.3	34933309.7	2	1	0	4.72	106	2310
>tr|F1PJY1|F1PJY1_CANLF Mannosyl-glycoprotein endo-beta-N-acetylglucosaminidase OS=Canis lupus familiaris OX=9615 GN=ENGASE PE=3 SV=3	0.1	0.04	137.4	3758465.7	2	1	1	1.74	690	32761
>sp|O46607|GPX5_CANLF Epididymal secretory glutathione peroxidase OS=Canis lupus familiaris OX=9615 GN=GPX5 PE=2 SV=1	0.73	0.71	76	19747744.3	2	1	0	6.79	221	564

>tr|J9P4Q5|J9P4Q5_CANLF HECT-type E3 ubiquitin transferase OS=Canis lupus familiaris OX=9615 GN=HECW1 PE=4 SV=2	0.1	0.03	105.1	16497583.2	1	1	0	1.26	1587	3937
>tr|A0A5F4C7Q7|A0A5F4C7Q7_CANLF IQ motif and Sec7 domain ArfGEF 1 OS=Canis lupus familiaris OX=9615 GN=IQSEC1 PE=3 SV=1	0.11	0.12	45.5	1862384	1	1	0	0.51	971	4269
>tr|E2R6E0|E2R6E0_CANLF Lipocln_cytosolic_FA-bd_dom domain-containing protein OS=Canis lupus familiaris OX=9615 GN=LCNL1 PE=3 SV=2	1.64	1.6	207.9	13803649.9	3	1	0	3.68	299	1932
>tr|J9NS29|J9NS29_CANLF Cystatin domain-containing protein OS=Canis lupus familiaris OX=9615 GN=LOC607874 PE=4 SV=2	4.61	2.46	289.6	114468559	10	3	0	16.29	313	30016
>tr|F1PR54|F1PR54_CANLF Lactotransferrin OS=Canis lupus familiaris OX=9615 GN=LTF PE=3 SV=1	21.93	4.03	351.3	622958393	69	11	0	14.41	708	40436
>tr|E2RDT3|E2RDT3_CANLF Protein Mdm4 OS=Canis lupus familiaris OX=9615 GN=MDM4 PE=3 SV=3	0.1	0.03	61.1	8668513.4	1	1	0	4.48	491	11032
>tr|F1PHA9|F1PHA9_CANLF Motile sperm domain containing 2 OS=Canis lupus familiaris OX=9615 GN=MOSPD2 PE=4 SV=3	0.36	0.36	212	5751740.5	1	1	0	0.97	518	10201
>sp|Q28895|NPC2_CANLF NPC intracellular cholesterol transporter 2 OS=Canis lupus familiaris OX=9615 GN=NPC2 PE=2 SV=1	10.26	4.49	381.7	191672362	21	4	0	30.2	149	153
>tr|A0A5F4C615|A0A5F4C615_CANLF Glucocorticoid receptor OS=Canis lupus familiaris OX=9615 GN=NR3C1 PE=3 SV=1	0.1	0.06	106.9	45155646.5	3	2	1	2.76	725	7049
>tr|A0A5F4BT89|A0A5F4BT89_CANLF Olfactory receptor OS=Canis lupus familiaris OX=9615 GN=OR5W6 PE=3 SV=1	0.1	0.02	111.4	14625569.8	1	1	1	6.95	302	29923

>tr|E2RE16|E2RE16_CANLF Non-specific serine/threonine protein kinase OS=Canis lupus familiaris OX=9615 GN=PAK4 PE=4 SV=1	0.29	0.12	101.6	20491308.1	10	1	0	0.84	592	12735
>tr|A0A5F4DD58|A0A5F4DD58_CANLF Phosphoinositide phospholipase C OS=Canis lupus familiaris OX=9615 GN=PLCD3 PE=4 SV=1	0.9	0.8	205.9	33023756.5	6	1	0	0.4	741	3088
>tr|A0A5F4D7Y5|A0A5F4D7Y5_CANLF Pleckstrin homology, MyTH4 and FERM domain containing H1 OS=Canis lupus familiaris OX=9615 GN=PLEKHH1 PE=4 SV=1	0.1	0.06	128.3	2760122	1	1	0	0.3	1342	5979
>tr|A0A5F4CSE7|A0A5F4CSE7_CANLF TYR_PHOSPHATASE_2 domain-containing protein OS=Canis lupus familiaris OX=9615 GN=PTP4A1 PE=4 SV=1	0.1	0.01	57.8	10216523.6	1	1	1	5.2	173	25182
>tr|A0A5F4CYM0|A0A5F4CYM0_CANLF Quiescin sulfhydryl oxidase 2 OS=Canis lupus familiaris OX=9615 GN=QSOX2 PE=4 SV=1	0.1	0.02	76.9	35835400.1	10	1	0	2.52	636	1464
>tr|E2RRF5|E2RRF5_CANLF RNA binding motif protein 19 OS=Canis lupus familiaris OX=9615 GN=RBM19 PE=4 SV=3	0.1	0.01	101.4	131832453	44	2	0	0.72	970	905
>tr|E2RG75|E2RG75_CANLF Inactive ribonuclease-like protein 9 OS=Canis lupus familiaris OX=9615 GN=RNASE9 PE=3 SV=2	2.36	1.63	372.3	7097027.3	4	2	1	4.55	198	41734
>tr|A0A5F4C9V7|A0A5F4C9V7_CANLF Roundabout guidance receptor 3 OS=Canis lupus familiaris OX=9615 GN=ROBO3 PE=4 SV=1	0.22	0.22	184.3	10638326.1	1	1	0	0.42	1421	3563
>sp|E2RKA8|RL32_CANLF 60S ribosomal protein L32 OS=Canis lupus familiaris OX=9615 GN=RPL32 PE=1 SV=1	1.38	0.04	94	358661370	68	2	0	4.44	135	275

>sp|Q9XT60|SRY_CANLF Sex-determining region Y protein OS=Canis lupus familiaris OX=9615 GN=SRY PE=3 SV=1	0.29	0.29	89.5	1904460.7	1	1	0	3.18	220	347
>tr|E2RH77|E2RH77_CANLF Ferric oxidoreductase domain-containing protein OS=Canis lupus familiaris OX=9615 GN=STEAP1 PE=4 SV=2	0.1	0.02	86.2	17584984.2	1	1	0	2.18	596	22587
>tr|A0A5K1V0D8|A0A5K1V0D8_CANLF Sulfatase 2 OS=Canis lupus familiaris OX=9615 GN=SULF2 PE=3 SV=1	0.82	0.8	179.3	7953313.6	2	1	0	0.35	859	1192
>tr|F1PBJ1|F1PBJ1_CANLF Methylcytosine dioxygenase TET OS=Canis lupus familiaris OX=9615 GN=TET3 PE=3 SV=2	0.1	0.02	32.5	25839500.3	2	1	0	0.28	1795	1529
>tr|J9PA70|J9PA70_CANLF Tigger transposable element derived 7 OS=Canis lupus familiaris OX=9615 GN=TIGD7 PE=3 SV=2	0.67	0.67	101.1	1765648.6	1	1	0	1.46	548	5607
>tr|E2RCT1|E2RCT1_CANLF WAP domain-containing protein OS=Canis lupus familiaris OX=9615 PE=4 SV=2	0.43	0.4	186.1	17984233.1	3	1	0	14.66	116	21717
>tr|F1PM73|F1PM73_CANLF Palmitoyltransferase OS=Canis lupus familiaris OX=9615 GN=ZDHHC23 PE=3 SV=3	0.24	0.16	55.5	41316872.2	5	1	0	3.98	427	22460
>tr|A0A5F4D9Z7|A0A5F4D9Z7_CANLF Zinc finger FYVE-type containing 19 OS=Canis lupus familiaris OX=9615 GN=ZFYVE19 PE=4 SV=1	0.74	0.75	131.7	16130682.1	1	1	0	1.53	392	13875
>tr|A0A5F4DHH0|A0A5F4DHH0_CANLF ATP binding cassette subfamily A member 1 OS=Canis lupus familiaris OX=9615 GN=ABCA1 PE=4 SV=1	0.1	0.02	39	2381312.6	7	1	0	0.23	2175	3709
>tr|J9P7Y2|J9P7Y2_CANLF Angiotensin-converting enzyme OS=Canis lupus familiaris OX=9615 GN=ACE2 PE=3 SV=1	0.1	0.02	134	33954793.5	4	1	0	1.62	804	1041
>sp|O18840|ACTB_CANLF Actin, cytoplasmic 1 OS=Canis lupus familiaris OX=9615 GN=ACTB PE=2 SV=3	0.1	0.06	190.6	26426006.5	3	1	0	2.93	375	642

>tr|A0A5F4C535|A0A5F4C535_CANLF ADAM metallopeptidase with thrombospondin type 1 motif 13 OS=Canis lupus familiaris OX=9615 GN=ADAMTS13 PE=4 SV=1	0.1	0.02	84.5	87599235.6	7	1	0	0.9	444	2664
>tr|F1PGK9|F1PGK9_CANLF ADAM metallopeptidase with thrombospondin type 1 motif 5 OS=Canis lupus familiaris OX=9615 GN=ADAMTS5 PE=4 SV=3	0.1	0.02	41	3440320.3	9	1	0	0.59	845	11956
>tr|A0A5F4CAK8|A0A5F4CAK8_CANLF Ankyrin repeat and sterile alpha motif domain containing 1B OS=Canis lupus familiaris OX=9615 GN=ANKS1B PE=4 SV=1	0.1	0	49.5	114257500	13	2	2	0.86	1281	31927
>tr|A0A5F4DHJ7|A0A5F4DHJ7_CANLF APC-binding protein EB1 OS=Canis lupus familiaris OX=9615 PE=3 SV=1	0.1	0	33.6	156720911	19	2	2	1.67	300	38718
>tr|J9P3H8|J9P3H8_CANLF ATM interactor OS=Canis lupus familiaris OX=9615 GN=ATMIN PE=4 SV=2	0.16	0.02	49.4	2616877.9	12	1	0	0.58	863	882
>tr|E2RHC5|E2RHC5_CANLF Baculoviral IAP repeat containing 7 OS=Canis lupus familiaris OX=9615 GN=BIRC7 PE=3 SV=3	0.1	0.06	139.8	3491016.8	2	1	0	2.95	271	10900
>tr|A0A5F4CR89|A0A5F4CR89_CANLF Voltage-dependent R-type calcium channel subunit alpha OS=Canis lupus familiaris OX=9615 GN=CACNA1E PE=3 SV=1	0.61	0.38	54.1	255892986	13	1	0	0.19	2688	1145
>tr|F1P7Z8|F1P7Z8_CANLF Coiled-coil domain-containing protein 93 OS=Canis lupus familiaris OX=9615 GN=CCDC93 PE=3 SV=2	0.1	0.03	34.8	42083001.7	5	1	0	0.63	631	1813
>tr|Q9XSV4|Q9XSV4_CANLF CE10 protein OS=Canis lupus familiaris OX=9615 GN=ce10 PE=2 SV=1	1.65	0.82	333.9	215728728	23	3	0	14.55	110	41542
>tr|J9NSS6|J9NSS6_CANLF DNA helicase OS=Canis lupus familiaris OX=9615 GN=CHD2 PE=4 SV=2	0.25	0.02	65.6	4531674.9	21	1	0	0.28	1780	1264
>tr|F1PUR2|F1PUR2_CANLF CHK1 checkpoint homolog OS=Canis lupus familiaris OX=9615 GN=CHEK1 PE=3 SV=3	0.1	0	49.3	23535628.7	6	1	0	0.88	454	4485

>tr|A0A5F4C7X0|A0A5F4C7X0_CANLF Centriolin OS=Canis lupus familiaris OX=9615 GN=CNTRL PE=4 SV=1	0.1	0.02	40.7	72513150.7	3	1	0	0.2	2506	3658
>tr|A0A5F4CCD0|A0A5F4CCD0_CANLF Cysteine rich secretory protein 2 OS=Canis lupus familiaris OX=9615 GN=CRISP2 PE=3 SV=1	0.89	0.84	177.5	351994.1	4	1	0	4.82	311	11017
>tr|F1PJH2|F1PJH2_CANLF FAT atypical cadherin 3 OS=Canis lupus familiaris OX=9615 GN=FAT3 PE=4 SV=3	0.1	0	50.4	214399990	30	2	2	0.11	4557	3984
>tr|E2R186|E2R186_CANLF Fibroblast growth factor receptor OS=Canis lupus familiaris OX=9615 GN=FGFR1 PE=3 SV=3	0.12	0.02	62.6	100670644	13	1	0	0.59	853	9797
>tr|E2RSW3|E2RSW3_CANLF Ferric chelate reductase 1 OS=Canis lupus familiaris OX=9615 GN=FRRS1 PE=3 SV=3	0.1	0.02	63.7	16726431.7	2	1	0	0.66	605	40434
>tr|A0A5F4D961|A0A5F4D961_CANLF GLIPR1 like 2 OS=Canis lupus familiaris OX=9615 GN=GLIPR1L2 PE=4 SV=1	0.1	0	49.6	7958313.5	10	1	0	2.4	167	10571
>tr|J9P436|J9P436_CANLF G protein-coupled receptor 45 OS=Canis lupus familiaris OX=9615 GN=GPR45 PE=4 SV=2	0.1	0.07	37.2	63003806.8	9	2	1	1.34	373	7720
>tr|A0A5F4C3M5|A0A5F4C3M5_CANLF IQ motif containing GTPase activating protein 2 OS=Canis lupus familiaris OX=9615 GN=IQGAP2 PE=4 SV=1	0.53	0.53	36.1	5414616	1	1	0	0.38	1577	1056
>tr|F6Y6X6|F6Y6X6_CANLF IQ motif containing GTPase activating protein 3 OS=Canis lupus familiaris OX=9615 GN=IQGAP3 PE=4 SV=2	0.18	0.06	91.1	326817609	19	3	1	0.95	1693	7673
>tr|J9P801|J9P801_CANLF Jade family PHD finger 2 OS=Canis lupus familiaris OX=9615 GN=JADE2 PE=4 SV=1	0.24	0.24	50.1	10235919.7	2	1	1	0.64	781	4296
>tr|A0A5F4CQZ1|A0A5F4CQZ1_CANLF LIM domain 7 OS=Canis lupus familiaris OX=9615 GN=LMO7 PE=4 SV=1	0.1	0	89.1	15154154.7	4	1	1	0.28	1806	10523
>tr|A0A5F4C5B9|A0A5F4C5B9_CANLF Glutathione S-transferase OS=Canis lupus familiaris OX=9615 GN=LOC481841 PE=3 SV=1	0.1	0.02	35.8	1151128.8	4	1	1	1.83	218	22818

>tr|A0A5F4BVF3|A0A5F4BVF3_CANLF Lactotransferrin OS=Canis lupus familiaris OX=9615 GN=LTF PE=3 SV=1	3.85	1.27	312.3	273240797	28	5	0	8.15	626	32850
>tr|J9P816|J9P816_CANLF E3 ubiquitin-protein transferase MAEA OS=Canis lupus familiaris OX=9615 GN=MAEA PE=4 SV=2	0.1	0	31.3	104483.4	1	1	1	1.41	355	1538
>tr|J9NZY7|J9NZY7_CANLF Midasin OS=Canis lupus familiaris OX=9615 GN=MDN1 PE=3 SV=2	0.1	0.02	40.7	72513150.7	3	1	0	0.09	5558	2169
>tr|A0A5F4CPE1|A0A5F4CPE1_CANLF Major facilitator superfamily domain containing 14B OS=Canis lupus familiaris OX=9615 GN=MFSD14B PE=4 SV=1	0.38	0.38	38.3	8631560.7	1	1	0	3.71	485	28321
>tr|A0A5F4DDL6|A0A5F4DDL6_CANLF Molybdenum cofactor sulfurase OS=Canis lupus familiaris OX=9615 GN=MOCOS PE=3 SV=1	0.1	0.05	129.2	30055326.6	3	1	0	0.57	876	14262
>tr|L7MTP6|L7MTP6_CANLF Myosin heavy chain 3 OS=Canis lupus familiaris OX=9615 GN=MYH3 PE=3 SV=1	0.1	0.03	35.9	32077979.8	3	1	0	0.26	1940	17787
>tr|F1PDQ3|F1PDQ3_CANLF Beta-nerve growth factor OS=Canis lupus familiaris OX=9615 GN=NGF PE=3 SV=3	0.1	0.07	37.2	63003806.8	9	3	2	2.08	240	6254
>sp|Q28895|NPC2_CANLF NPC intracellular cholesterol transporter 2 OS=Canis lupus familiaris OX=9615 GN=NPC2 PE=2 SV=1	0.1	0.02	137.5	13609297.8	2	2	0	21.48	149	153
>tr|A0A5F4DCB2|A0A5F4DCB2_CANLF NOP2/Sun RNA methyltransferase 5 OS=Canis lupus familiaris OX=9615 GN=NSUN5 PE=3 SV=1	0.1	0.05	45.7	20836011.4	1	1	0	1.49	469	2010
>tr|E2RE16|E2RE16_CANLF Non-specific serine/threonine protein kinase OS=Canis lupus familiaris OX=9615 GN=PAK4 PE=4 SV=1	0.16	0.02	48.5	5005744.2	22	1	0	0.84	592	12735
>tr|E2RIK1|E2RIK1_CANLF Phosphatidylinositol-4-phosphate 3-kinase OS=Canis lupus familiaris OX=9615 GN=PIK3C2G PE=3 SV=3	0.1	0.01	86.4	67553350.1	3	1	0	0.42	1445	5924

>tr|A0A5F4DD58|A0A5F4DD58_CANLF Phosphoinositide phospholipase C OS=Canis lupus familiaris OX=9615 GN=PLCD3 PE=4 SV=1	0.24	0.02	146.6	656412059	83	4	1	1.48	741	3088
>tr|E2RRM5|E2RRM5_CANLF Ras interacting protein 1 OS=Canis lupus familiaris OX=9615 GN=RASIP1 PE=4 SV=3	0.16	0.16	33.9	265549.4	1	1	0	0.62	1137	25466
>tr|A0A5F4CVL6|A0A5F4CVL6_CANLF RB transcriptional corepressor 1 OS=Canis lupus familiaris OX=9615 GN=RB1 PE=3 SV=1	0.1	0.02	34.7	21674741.6	18	1	1	0.43	927	1490
>tr|F1PQ34|F1PQ34_CANLF RCC1 and BTB domain containing protein 2 OS=Canis lupus familiaris OX=9615 GN=RCBTB2 PE=4 SV=3	0.1	0.02	63.7	9027790.8	1	1	0	1.23	326	6990
>tr|F1PP47|F1PP47_CANLF RE1 silencing transcription factor OS=Canis lupus familiaris OX=9615 GN=REST PE=4 SV=2	0.1	0.03	107.1	41062721.9	5	2	1	0.9	996	17823
>sp|Q9XSU7|RL27_CANLF 60S ribosomal protein L27 OS=Canis lupus familiaris OX=9615 GN=RPL27 PE=2 SV=3	0.54	0.02	42.8	44466788.4	74	1	0	3.68	136	314
>tr|A0A5F4D6L9|A0A5F4D6L9_CANLF Sacsin molecular chaperone OS=Canis lupus familiaris OX=9615 GN=SACS PE=4 SV=1	0.1	0.01	75.3	331955035	43	1	0	0.09	4500	1444
>tr|F1PPN1|F1PPN1_CANLF Scaffold attachment factor B2 OS=Canis lupus familiaris OX=9615 GN=SAFB2 PE=4 SV=3	0.15	0.07	69.4	92373389	5	1	0	0.63	954	22763
>tr|F1Q3Q6|F1Q3Q6_CANLF Secernin 1 OS=Canis lupus familiaris OX=9615 GN=SCRN1 PE=3 SV=2	0.1	0.01	55.9	108714	1	1	1	3.65	438	38892
>tr|A0A5F4D850|A0A5F4D850_CANLF Structural maintenance of chromosomes protein OS=Canis lupus familiaris OX=9615 GN=SMC3 PE=3 SV=1	0.1	0.02	40.7	72513150.7	3	1	0	0.41	1219	7467
>tr|E2RIV7|E2RIV7_CANLF Syntrophin alpha 1 OS=Canis lupus familiaris OX=9615 GN=SNTA1 PE=3 SV=3	0.1	0.07	36.5	5810887	8	1	1	1.03	486	34454

>tr|F1PGF6|F1PGF6_CANLF Sprouty related EVH1 domain containing 1 OS=Canis lupus familiaris OX=9615 GN=SPRED1 PE=4 SV=2	0.24	0.02	107.1	258992145	20	1	0	0.9	443	10620
>tr|F1P6X5|F1P6X5_CANLF ST6 N-acetylgalactosaminide alpha-2,6-sialyltransferase 5 OS=Canis lupus familiaris OX=9615 GN=ST6GALNAC5 PE=3 SV=3	0.22	0.02	84.5	246113204	19	1	0	1.18	338	4902
>tr|F1PBJ1|F1PBJ1_CANLF Methylcytosine dioxygenase TET OS=Canis lupus familiaris OX=9615 GN=TET3 PE=3 SV=2	0.1	0	86.8	53409123.6	5	1	0	0.28	1795	1529
>tr|A0A5F4C9Z9|A0A5F4C9Z9_CANLF UTP20 small subunit processome component OS=Canis lupus familiaris OX=9615 GN=UTP20 PE=4 SV=1	0.15	0.15	72.6	33460592.3	2	2	1	0.44	2758	1779
>sp|Q28894|WFDC2_CANLF WAP four-disulfide core domain protein 2 OS=Canis lupus familiaris OX=9615 GN=WFDC2 PE=2 SV=1	2.29	2.07	427.5	71379011.9	12	1	0	6.45	124	53
>tr|J9P5T2|J9P5T2_CANLF Non-specific serine/threonine protein kinase OS=Canis lupus familiaris OX=9615 GN=WNK3 PE=4 SV=2	0.1	0	45.1	89526200.9	11	1	0	0.22	2294	5229
>tr|J9PAQ2|J9PAQ2_CANLF Cyclin N-terminal domain-containing protein OS=Canis lupus familiaris OX=9615 PE=3 SV=1	0.62	0.38	54.1	255892986	13	1	0	1.25	400	2385
>tr|J9P2T7|J9P2T7_CANLF 26S proteasome non-ATPase regulatory subunit 5 OS=Canis lupus familiaris OX=9615 PE=4 SV=1	0.89	0.89	76.8	13195937.6	1	1	0	1.95	461	23870
>tr|J9P7Y2|J9P7Y2_CANLF Angiotensin-converting enzyme OS=Canis lupus familiaris OX=9615 GN=ACE2 PE=3 SV=1	0.1	0.02	90.8	49895948.3	3	1	0	1.62	804	1041
>sp|O18840|ACTB_CANLF Actin, cytoplasmic 1 OS=Canis lupus familiaris OX=9615 GN=ACTB PE=2 SV=3	1.78	1.56	240.9	226071179	12	2	0	7.73	375	642

>tr|A0A5F4BTW9|A0A5F4BTW9_CANLF Adhesion G protein-coupled receptor L2 OS=Canis lupus familiaris OX=9615 GN=ADGRL2 PE=4 SV=1	0.19	0.19	128.4	17274808.2	1	1	0	0.47	1474	2238
>tr|F1PVA2|F1PVA2_CANLF Adhesion G protein-coupled receptor V1 OS=Canis lupus familiaris OX=9615 GN=ADGRV1 PE=4 SV=3	0.32	0.33	173.4	15282101.3	1	1	0	0.08	6300	19804
>sp|Q2PQH8|GDE_CANLF Glycogen debranching enzyme OS=Canis lupus familiaris OX=9615 GN=AGL PE=2 SV=1	0.51	0.5	81.4	36251439.8	2	1	0	0.13	1533	23
>sp|P49822|ALBU_CANLF Albumin OS=Canis lupus familiaris OX=9615 GN=ALB PE=1 SV=3	10.77	4.02	295.2	624251171	36	7	0	14.8	608	490
>tr|A0A5F4DGF5|A0A5F4DGF5_CANLF Alkaline phosphatase OS=Canis lupus familiaris OX=9615 GN=ALPL PE=3 SV=1	5.03	3.46	347.9	69987456.6	7	3	0	5.77	572	6357
>tr|J9NS28|J9NS28_CANLF RBR-type E3 ubiquitin transferase OS=Canis lupus familiaris OX=9615 GN=ANKIB1 PE=4 SV=2	0.1	0.06	47.2	18191412.1	1	1	0	2.33	988	26345
>tr|F1PI09|F1PI09_CANLF Aldehyde oxidase OS=Canis lupus familiaris OX=9615 GN=AOX2 PE=3 SV=3	2.39	2.29	228.5	96041029.3	6	1	0	0.67	1347	21650
>tr|F1PGF9|F1PGF9_CANLF Rho guanine nucleotide exchange factor 26 OS=Canis lupus familiaris OX=9615 GN=ARHGEF26 PE=4 SV=3	0.57	0.53	266.9	76427460.5	3	1	0	0.84	594	22876
>tr|E2RA54|E2RA54_CANLF Bromodomain and WD repeat domain containing 3 OS=Canis lupus familiaris OX=9615 GN=BRWD3 PE=4 SV=3	0.67	0.56	187.2	97944614.4	11	2	0	0.8	1750	4294
>tr|E2QX33|E2QX33_CANLF Coiled-coil and C2 domain containing 1A OS=Canis lupus familiaris OX=9615 GN=CC2D1A PE=3 SV=1	0.1	0.03	148.2	55979391.6	5	1	1	0.84	951	10961
>tr|Q9XSV4|Q9XSV4_CANLF CE10 protein OS=Canis lupus familiaris OX=9615 GN=ce10 PE=2 SV=1	5.15	3.99	327.8	408366089	35	3	0	12.73	110	41542

>sp|Q6AW47|EST5A_CANLF Carboxylesterase 5A OS=Canis lupus familiaris OX=9615 GN=CES5A PE=2 SV=1	0.7	0.36	189.8	36484739.2	2	2	0	1.57	575	629
>sp|P25473|CLUS_CANLF Clusterin OS=Canis lupus familiaris OX=9615 GN=CLU PE=2 SV=1	0.38	0.34	218.4	53142113.9	3	1	0	0.9	445	725
>tr|E2R8U5|E2R8U5_CANLF Collagen type XXIV alpha 1 chain OS=Canis lupus familiaris OX=9615 GN=COL24A1 PE=4 SV=3	0.1	0.1	124.2	31867195.8	2	2	1	0.82	1709	25162
>tr|A0A5F4CCD0|A0A5F4CCD0_CANLF Cysteine rich secretory protein 2 OS=Canis lupus familiaris OX=9615 GN=CRISP2 PE=3 SV=1	1.32	1.26	213.5	75936743.2	5	2	0	7.07	311	11017
>tr|F1PLV2|F1PLV2_CANLF Peptidyl-prolyl cis-trans isomerase OS=Canis lupus familiaris OX=9615 GN=CSNK1G1 PE=3 SV=3	0.67	0.65	193	45151630.6	3	1	0	5.35	243	4290
>sp|Q9MZY0|CP2E1_CANLF Cytochrome P450 2E1 OS=Canis lupus familiaris OX=9615 GN=CYP2E1 PE=2 SV=1	0.1	0.03	165.9	103304842	6	3	2	4.66	494	522
>tr|A0A5F4DJ83|A0A5F4DJ83_CANLF DExH-box helicase 57 OS=Canis lupus familiaris OX=9615 GN=DHX57 PE=4 SV=1	1.09	1.07	168.4	4849595.7	2	1	0	0.64	1411	5166
>tr|A0A5F4BSQ1|A0A5F4BSQ1_CANLF Dystonin OS=Canis lupus familiaris OX=9615 GN=DST PE=4 SV=1	0.17	0.18	114.1	15848542.5	1	1	0	0.17	7514	9739
>tr|A0A5F4D3J0|A0A5F4D3J0_CANLF Dymeclin OS=Canis lupus familiaris OX=9615 GN=DYM PE=3 SV=1	0.46	0.35	122.8	25111507.9	2	2	2	1.54	712	10307
>tr|F1PJY1|F1PJY1_CANLF Mannosyl-glycoprotein endo-beta-N-acetylglucosaminidase OS=Canis lupus familiaris OX=9615 GN=ENGASE PE=3 SV=3	1.06	0.39	154	146672909	17	3	2	1.74	690	32761
>tr|J9P3V5|J9P3V5_CANLF FAM75 domain-containing protein OS=Canis lupus familiaris OX=9615 PE=4 SV=2	0.1	0.08	173.8	34311408.5	2	1	0	0.36	1370	32063

>tr|A0A5F4CTH2|A0A5F4CTH2_CANLF FA complementation group I OS=Canis lupus familiaris OX=9615 GN=FANCI PE=4 SV=1	0.35	0.35	120.8	17259280.1	1	1	0	0.85	1404	5579
>tr|A0A5F4D952|A0A5F4D952_CANLF FAT atypical cadherin 1 OS=Canis lupus familiaris OX=9615 GN=FAT1 PE=4 SV=1	0.42	0.43	163.9	15585287.4	1	1	0	0.26	4614	14224
>tr|A0A5F4DI80|A0A5F4DI80_CANLF Zinc finger protein Gfi-1 OS=Canis lupus familiaris OX=9615 GN=GFI1 PE=4 SV=1	1.14	1.12	115.4	907642.9	2	1	1	0.99	912	9754
>tr|F1PJ71|F1PJ71_CANLF Glutathione peroxidase OS=Canis lupus familiaris OX=9615 GN=GPX5 PE=3 SV=2	6.5	2.91	302.2	318253471	21	6	1	29.86	221	19009
>tr|A0A5F4CLI1|A0A5F4CLI1_CANLF Histone deacetylase 6 OS=Canis lupus familiaris OX=9615 GN=HDAC6 PE=4 SV=1	0.49	0.45	172.7	54364173.4	3	1	0	0.43	1175	4057
>tr|E2RGH9|E2RGH9_CANLF HECT-type E3 ubiquitin transferase OS=Canis lupus familiaris OX=9615 GN=HECTD1 PE=3 SV=2	0.1	0.06	147.4	33389415.2	2	1	0	0.31	2609	36968
>tr|A0A5F4BPM5|A0A5F4BPM5_CANLF ILK associated serine/threonine phosphatase OS=Canis lupus familiaris OX=9615 GN=ILKAP PE=3 SV=1	1.51	1.45	218.5	12669300.9	4	1	1	2.79	359	7963
>tr|A0A5F4C840|A0A5F4C840_CANLF KIAA1109 OS=Canis lupus familiaris OX=9615 GN=KIAA1109 PE=4 SV=1	0.47	0.46	176.1	19808731.3	2	1	0	0.16	5041	21640
>tr|A0A5F4C7E7|A0A5F4C7E7_CANLF 3-beta-hydroxysterol Delta (14)-reductase OS=Canis lupus familiaris OX=9615 GN=LBR PE=3 SV=1	0.1	0.06	140.9	16271171	1	1	0	1.04	576	1034
>tr|E2R6E0|E2R6E0_CANLF Lipocln_cytosolic_FA-bd_dom domain-containing protein OS=Canis lupus familiaris OX=9615 GN=LCNL1 PE=3 SV=2	2.4	2.36	135.4	54394337.6	3	1	0	3.01	299	1932

>tr|A0A5F4CB08|A0A5F4CB08_CANLF Glutathione transferase OS=Canis lupus familiaris OX=9615 GN=LOC100856518 PE=3 SV=1	0.58	0.56	199.4	71054194.6	4	1	0	3.08	292	4491
>tr|F1PR54|F1PR54_CANLF Lactotransferrin OS=Canis lupus familiaris OX=9615 GN=LTF PE=3 SV=1	19.74	4.25	327.4	1267254089	84	13	1	18.22	708	40436
>sp|Q7YRB7|AOFB_CANLF Amine oxidase [flavin-containing] B OS=Canis lupus familiaris OX=9615 GN=MAOB PE=2 SV=3	0.45	0.45	128.5	17226955.8	1	1	0	0.77	520	646
>sp|F1PRN2|MYO1D_CANLF Unconventional myosin-Id OS=Canis lupus familiaris OX=9615 GN=MYO1D PE=1 SV=2	0.51	0.49	158.6	29831028.2	2	1	0	0.3	1006	763
>sp|Q28895|NPC2_CANLF NPC intracellular cholesterol transporter 2 OS=Canis lupus familiaris OX=9615 GN=NPC2 PE=2 SV=1	18.28	5.26	587.3	788208902	68	4	0	36.91	149	153
>tr|E2RHG5|E2RHG5_CANLF Nudix hydrolase 3 OS=Canis lupus familiaris OX=9615 GN=NUDT3 PE=4 SV=1	0.45	0.39	136	15525663.2	4	1	0	6.4	172	21894
>tr|A0A5F4CCE2|A0A5F4CCE2_CANLF Pappalysin 2 OS=Canis lupus familiaris OX=9615 GN=PAPPA2 PE=3 SV=1	1.63	1.63	155.3	19488411.9	1	1	0	0.81	1722	20443
>sp|Q9XS65|PTGDS_CANLF Prostaglandin-H2 D-isomerase OS=Canis lupus familiaris OX=9615 GN=PTGDS PE=2 SV=1	3.45	1.81	308.7	368463965	17	2	1	10.47	191	165
>tr|A0A5F4DHE4|A0A5F4DHE4_CANLF Protein tyrosine phosphatase non-receptor type 22 OS=Canis lupus familiaris OX=9615 GN=PTPN22 PE=4 SV=1	0.54	0.51	173.3	75764927.7	4	2	1	2.42	784	5054
>sp|F1PTE3|RAB13_CANLF Ras-related protein Rab-13 OS=Canis lupus familiaris OX=9615 GN=RAB13 PE=1 SV=2	0.5	0.23	142.9	95584726	6	2	0	2.46	203	147
>sp|Q5TJE5|RGL2_CANLF Ral guanine nucleotide dissociation stimulator-like 2 OS=Canis lupus familiaris OX=9615 GN=RGL2 PE=3 SV=1	1.32	0.93	101	61436340.9	4	2	0	0.64	780	266

>tr|A0A5F4DCA4|A0A5F4DCA4_CANLF Reverse transcriptase domain-containing protein OS=Canis lupus familiaris OX=9615 PE=4 SV=1	0.51	0.49	144.6	29591909.8	2	1	0	0.31	978	860
>tr|F6XAM4|F6XAM4_CANLF Peptidylprolyl isomerase OS=Canis lupus familiaris OX=9615 GN=SCRN1 PE=4 SV=2	0.2	0.2	122.1	24306589.2	1	1	0	7.05	241	38880
>tr|E2R079|E2R079_CANLF Serpin family B member 2 OS=Canis lupus familiaris OX=9615 GN=SERPINB2 PE=3 SV=2	0.2	0.2	150.1	19354015.4	1	1	0	1.92	416	21385
>tr|F1PBU5|F1PBU5_CANLF Non-specific serine/threonine protein kinase OS=Canis lupus familiaris OX=9615 GN=SMG1 PE=3 SV=3	0.95	0.39	163.6	167474779	11	2	0	0.17	3634	6898
>tr|E2QV77|E2QV77_CANLF SPRY domain containing 4 OS=Canis lupus familiaris OX=9615 GN=SPRYD4 PE=4 SV=2	0.55	0.55	71	511164.3	1	1	0	3.94	254	8010
>tr|A0A5F4CHN1|A0A5F4CHN1_CANLF Serine/arginine repetitive matrix 2 OS=Canis lupus familiaris OX=9615 GN=SRRM2 PE=4 SV=1	0.66	0.66	197.1	19302627.6	1	1	0	0.16	2564	3180
>tr|A0A5F4CK18|A0A5F4CK18_CANLF Transcription elongation factor spt6 OS=Canis lupus familiaris OX=9615 GN=SUPT6H PE=3 SV=1	0.1	0.08	153	15837347.8	1	1	0	0.44	1602	3373
>tr|J9P0B4|J9P0B4_CANLF Tudor domain containing 15 OS=Canis lupus familiaris OX=9615 GN=TDRD15 PE=4 SV=2	1.59	1.43	231.6	186749629	9	1	0	0.57	2105	4188
>tr|A0A5F4D430|A0A5F4D430_CANLF Transcription factor AP-2 gamma OS=Canis lupus familiaris OX=9615 GN=TFAP2C PE=3 SV=1	0.34	0.34	165.2	16336580.4	1	1	0	1.38	650	9642
>sp|Q5I2M8|TLR9_CANLF Toll-like receptor 9 OS=Canis lupus familiaris OX=9615 GN=TLR9 PE=2 SV=1	0.43	0.43	140.2	5220431.7	1	1	0	1.16	1032	382

>sp|Q28894|WFDC2_CANLF WAP four-disulfide core domain protein 2 OS=Canis lupus familiaris OX=9615 GN=WFDC2 PE=2 SV=1	3.64	3.62	426.7	13881147.4	2	1	0	6.45	124	53
>tr|F1P9J6|F1P9J6_CANLF Zinc finger protein 16 OS=Canis lupus familiaris OX=9615 GN=ZNF16 PE=4 SV=3	0.38	0.36	158.4	38657674.5	2	1	0	1.47	680	39751
>tr|A0A5F4DHH0|A0A5F4DHH0_CANLF ATP binding cassette subfamily A member 1 OS=Canis lupus familiaris OX=9615 GN=ABCA1 PE=4 SV=1	0.27	0.25	34.8	1829194.8	2	1	0	0.23	2175	3709
>tr|A0A5F4CQ96|A0A5F4CQ96_CANLF ATP binding cassette subfamily G member 8 OS=Canis lupus familiaris OX=9615 GN=ABCG8 PE=3 SV=1	0.19	0.17	185.9	9998253.4	2	1	0	1.84	707	17201
>tr|J9PAF1|J9PAF1_CANLF Actin binding LIM protein family member 2 OS=Canis lupus familiaris OX=9615 GN=ABLIM2 PE=4 SV=2	0.5	0.5	119.6	4474940	1	1	0	1.71	645	4331
>sp|O18840|ACTB_CANLF Actin, cytoplasmic 1 OS=Canis lupus familiaris OX=9615 GN=ACTB PE=2 SV=3	7.57	4.92	325.7	148633566	22	2	0	5.33	375	642
>tr|E2R4A0|E2R4A0_CANLF Actin-like protein 7B OS=Canis lupus familiaris OX=9615 GN=ACTL7B PE=3 SV=2	0.33	0.31	150.8	11667250	2	1	1	2.42	454	3066
>tr|A0A5F4D2X6|A0A5F4D2X6_CANLF AF4/FMR2 family member 3 OS=Canis lupus familiaris OX=9615 GN=AFF3 PE=3 SV=1	1.11	1.11	242.4	7260522.1	1	1	0	0.41	1213	3298
>sp|P49822|ALBU_CANLF Albumin OS=Canis lupus familiaris OX=9615 GN=ALB PE=1 SV=3	0.3	0.25	178.5	33756533.6	7	2	1	2.3	608	490
>tr|J9NS28|J9NS28_CANLF RBR-type E3 ubiquitin transferase OS=Canis lupus familiaris OX=9615 GN=ANKIB1 PE=4 SV=2	0.23	0.17	62.7	27866905.8	4	1	0	2.33	988	26345
>tr|A0A5F4D105|A0A5F4D105_CANLF Amyloid beta precursor protein binding family A member 1 OS=Canis lupus familiaris OX=9615 GN=APBA1 PE=4 SV=1	0.1	0.1	50.4	358932.2	1	1	0	0.56	889	3602

>sp|P83509|RHG35_CANLF Rho GTPase-activating protein 35 OS=Canis lupus familiaris OX=9615 GN=ARHGAP35 PE=2 SV=1	0.55	0.47	115.2	25749029.1	5	1	1	0.73	1500	323
>tr|F1PGF9|F1PGF9_CANLF Rho guanine nucleotide exchange factor 26 OS=Canis lupus familiaris OX=9615 GN=ARHGEF26 PE=4 SV=3	1.24	1.2	296.7	25220664	3	1	0	0.84	594	22876
>sp|P62286|ASPM_CANLF Abnormal spindle-like microcephaly-associated protein homolog OS=Canis lupus familiaris OX=9615 GN=ASPM PE=2 SV=2	0.6	0.49	150.8	394855698	87	3	0	0.23	3469	677
>tr|A0A5F4C1S8|A0A5F4C1S8_CANLF E3 ubiquitin-protein ligase CBL OS=Canis lupus familiaris OX=9615 GN=CBL PE=4 SV=1	0.19	0.17	144.9	6581470.8	2	1	0	0.39	773	1308
>tr|Q9XSV4|Q9XSV4_CANLF CE10 protein OS=Canis lupus familiaris OX=9615 GN=ce10 PE=2 SV=1	1.38	1.12	233.1	19989969.8	6	3	0	14.55	110	41542
>tr|E2R4F0|E2R4F0_CANLF Cadherin EGF LAG seven-pass G-type receptor 2 OS=Canis lupus familiaris OX=9615 GN=CELSR2 PE=3 SV=2	0.14	0.04	72.2	33750901.4	6	3	3	0.65	2919	12898
>tr|A0A5F4D9S5|A0A5F4D9S5_CANLF Hyaluronoglucosaminidase OS=Canis lupus familiaris OX=9615 GN=CEMIP PE=3 SV=1	0.59	0.59	158.6	335957.8	1	1	0	0.24	1684	9775
>tr|A0A5F4CAT6|A0A5F4CAT6_CANLF Centromere protein I OS=Canis lupus familiaris OX=9615 GN=CENPI PE=3 SV=1	0.17	0.17	86.8	4393225.8	1	1	1	1.29	699	20654
>sp|P21842|CMA1_CANLF Chymase OS=Canis lupus familiaris OX=9615 GN=CMA1 PE=1 SV=1	0.21	0.11	89.8	25921456	6	1	0	0.8	249	34
>tr|A0A5F4CCD0|A0A5F4CCD0_CANLF Cysteine rich secretory protein 2 OS=Canis lupus familiaris OX=9615 GN=CRISP2 PE=3 SV=1	0.3	0.3	170.4	4780781.6	1	1	0	2.25	311	11017

>tr|A0A5F4CAH2|A0A5F4CAH2_CANLF RNA polymerase II subunit A C-terminal domain phosphatase OS=Canis lupus familiaris OX=9615 GN=CTDP1 PE=4 SV=1	0.3	0.3	102.7	10002869.5	2	1	0	0.74	945	14396
>tr|Q30KS5|Q30KS5_CANLF Beta-defensin 129 OS=Canis lupus familiaris OX=9615 GN=DEFB129 PE=2 SV=1	1	0.96	279	21465779.2	3	1	0	4.22	166	41730
>tr|J9NYC7|J9NYC7_CANLF Dynein axonemal heavy chain 12 OS=Canis lupus familiaris OX=9615 GN=DNAH12 PE=3 SV=1	0.23	0.19	122.1	13622353.3	3	1	0	0.33	3960	15992
>tr|A0A5F4DIZ9|A0A5F4DIZ9_CANLF Dynein axonemal heavy chain 3 OS=Canis lupus familiaris OX=9615 GN=DNAH3 PE=3 SV=1	0.19	0.17	136.3	6342296.9	3	2	1	0.37	3229	26729
>tr|F1PPP9|F1PPP9_CANLF Family with sequence similarity 135 member A OS=Canis lupus familiaris OX=9615 GN=FAM135A PE=3 SV=3	0.49	0.5	114.1	933504.9	1	1	0	1.22	1399	6815
>tr|E2R186|E2R186_CANLF Fibroblast growth factor receptor OS=Canis lupus familiaris OX=9615 GN=FGFR1 PE=3 SV=3	0.16	0.03	95.3	7524778.9	9	1	0	0.59	853	9797
>tr|A0A5F4CZE8|A0A5F4CZE8_CANLF FAD dependent oxidoreductase domain containing 2 OS=Canis lupus familiaris OX=9615 GN=FOXRED2 PE=4 SV=1	0.19	0.17	34.4	10730748.4	2	1	1	4.84	723	4094
>tr|E2R7J0|E2R7J0_CANLF FRA10A associated CGG repeat 1 OS=Canis lupus familiaris OX=9615 GN=FRA10AC1 PE=4 SV=2	0.13	0.13	117.3	9912961.9	1	1	0	2.24	313	9020
>sp|Q9TU69|GHR_CANLF Growth hormone receptor OS=Canis lupus familiaris OX=9615 GN=GHR PE=2 SV=1	0.12	0.1	140.5	17128942.5	2	1	0	1.72	638	541
>tr|J9NRV0|J9NRV0_CANLF Histone H4 OS=Canis lupus familiaris OX=9615 GN=H4C11 PE=3 SV=2	0.19	0.17	150.1	11236927.7	2	1	1	21.78	101	7932
>tr|F1PQZ1|F1PQZ1_CANLF Hyaluronan binding protein 4 OS=Canis lupus familiaris OX=9615 GN=HABP4 PE=4 SV=2	0.15	0.09	161.8	35234505.3	4	1	0	7.91	215	39227

>tr|J9PAZ6|J9PAZ6_CANLF Hyperpolarization activated cyclic nucleotide gated potassium channel 4 OS=Canis lupus familiaris OX=9615 GN=HCN4 PE=4 SV=2	0.21	0.17	103.4	21839012.4	3	1	1	4.91	530	19838
>tr|J9P870|J9P870_CANLF Insulin like growth factor binding protein 6 OS=Canis lupus familiaris OX=9615 GN=IGFBP6 PE=4 SV=2	0.16	0.16	98.3	5066264	1	1	0	4.29	303	29295
>tr|E2RRP1|E2RRP1_CANLF Butyryl-CoA dehydrogenase OS=Canis lupus familiaris OX=9615 GN=IVD PE=3 SV=2	0.12	0.08	95.5	30783500.3	4	1	0	3.99	426	10739
>tr|E2R6E0|E2R6E0_CANLF Lipocln_cytosolic_FA-bd_dom domain-containing protein OS=Canis lupus familiaris OX=9615 GN=LCNL1 PE=3 SV=2	2.66	1.39	209.5	12838532.2	3	2	0	6.69	299	1932
>tr|E2RRP3|E2RRP3_CANLF LIM homeobox 5 OS=Canis lupus familiaris OX=9615 GN=LHX5 PE=4 SV=1	0.12	0.12	136.3	9045969.1	1	1	0	2.24	402	19607
>tr|F1PKS5|F1PKS5_CANLF IRF tryptophan pentad repeat domain-containing protein OS=Canis lupus familiaris OX=9615 GN=LOC609817 PE=4 SV=3	0.14	0.12	76.9	10753371.3	2	1	1	3.17	441	4897
>tr|A0A5F4BVF3|A0A5F4BVF3_CANLF Lactotransferrin OS=Canis lupus familiaris OX=9615 GN=LTF PE=3 SV=1	8.76	3.38	331.9	192363156	39	6	0	9.42	626	32850
>tr|A0A5F4CNT4|A0A5F4CNT4_CANLF Microtubule actin crosslinking factor 1 OS=Canis lupus familiaris OX=9615 GN=MACF1 PE=4 SV=1	0.11	0.12	59.5	242137.8	1	1	0	0.19	7352	1251
>tr|A0A5F4C951|A0A5F4C951_CANLF F-actin monooxygenase OS=Canis lupus familiaris OX=9615 GN=MICAL3 PE=3 SV=1	0.23	0.23	102.8	10785253.8	1	1	1	0.38	2113	4569
>tr|A0A5F4BZW4|A0A5F4BZW4_CANLF Malonyl-CoA decarboxylase OS=Canis lupus familiaris OX=9615 GN=MLYCD PE=4 SV=1	0.25	0.17	204.2	34887633.1	5	1	0	1.3	461	4809
>sp|E2QRY6|NNRE_CANLF NAD(P)H-hydrate epimerase OS=Canis lupus familiaris OX=9615 GN=NAXE PE=3 SV=1	0.55	0.55	42.4	310792.8	1	1	0	1.74	288	159

>tr|A0A5F4CUI6|A0A5F4CUI6_CANLF NLR family pyrin domain containing 3 OS=Canis lupus familiaris OX=9615 GN=NLRP3 PE=3 SV=1	0.24	0.25	76.2	5814371.9	1	1	1	0.89	1007	9702
>tr|J9P9K7|J9P9K7_CANLF Glycylpeptide N-tetradecanoyltransferase OS=Canis lupus familiaris OX=9615 GN=NMT2 PE=3 SV=2	0.31	0.29	76.1	12076877.6	2	1	0	3.16	507	19368
>sp|Q28895|NPC2_CANLF NPC intracellular cholesterol transporter 2 OS=Canis lupus familiaris OX=9615 GN=NPC2 PE=2 SV=1	0.79	0.68	102.4	460040.4	2	2	0	14.77	149	153
>tr|A0A5F4BT89|A0A5F4BT89_CANLF Olfactory receptor OS=Canis lupus familiaris OX=9615 GN=OR5W6 PE=3 SV=1	0.64	0.64	86.5	7580025.7	1	1	1	6.95	302	29923
>tr|E2RE16|E2RE16_CANLF Non-specific serine/threonine protein kinase OS=Canis lupus familiaris OX=9615 GN=PAK4 PE=4 SV=1	0.17	0.17	58.7	954967.4	1	1	0	0.84	592	12735
>tr|E2RA12|E2RA12_CANLF PH domain and leucine rich repeat protein phosphatase 2 OS=Canis lupus familiaris OX=9615 GN=PHLPP2 PE=4 SV=3	0.16	0.16	123.8	3110773.8	1	1	1	0.44	1351	11085
>tr|A0A5F4D7Y5|A0A5F4D7Y5_CANLF Pleckstrin homology, MyTH4 and FERM domain containing H1 OS=Canis lupus familiaris OX=9615 GN=PLEKHH1 PE=4 SV=1	0.16	0.16	203.3	6699243.3	1	1	0	0.3	1342	5979
>tr|A0A5F4C854|A0A5F4C854_CANLF Proline-serine-threonine phosphatase interacting protein 1 OS=Canis lupus familiaris OX=9615 GN=PSTPIP1 PE=4 SV=1	0.21	0.21	55.2	319542.4	1	1	0	3.55	141	9178
>sp|Q9XS65|PTGDS_CANLF Prostaglandin-H2 D-isomerase OS=Canis lupus familiaris OX=9615 GN=PTGDS PE=2 SV=1	4.2	2.78	330.7	123756321	18	3	1	13.61	191	165

>tr|E2RRF5|E2RRF5_CANLF RNA binding motif protein 19 OS=Canis lupus familiaris OX=9615 GN=RBM19 PE=4 SV=3	0.13	0.11	43.3	3068568.4	2	1	0	0.41	970	905
>sp|E2RKA8|RL32_CANLF 60S ribosomal protein L32 OS=Canis lupus familiaris OX=9615 GN=RPL32 PE=1 SV=1	1.9	0.15	167.3	351563559	87	2	0	4.44	135	275
>tr|A0A5F4DCA4|A0A5F4DCA4_CANLF Reverse transcriptase domain-containing protein OS=Canis lupus familiaris OX=9615 PE=4 SV=1	0.71	0.54	137.4	10686770.4	3	2	0	0.61	978	860
>tr|A0A5F4D6L9|A0A5F4D6L9_CANLF Sacsin molecular chaperone OS=Canis lupus familiaris OX=9615 GN=SACS PE=4 SV=1	0.2	0.14	226.8	30959033.6	9	4	0	0.58	4500	1444
>tr|E2QY94|E2QY94_CANLF Sphingomyelin synthase 2 OS=Canis lupus familiaris OX=9615 GN=SGMS2 PE=3 SV=1	0.17	0.17	60	6833113.2	1	1	1	4.93	365	18937
>tr|A0A5F4BZS6|A0A5F4BZS6_CANLF Solute carrier family 41 member 3 OS=Canis lupus familiaris OX=9615 GN=SLC41A3 PE=3 SV=1	0.26	0.26	54.4	6216044.8	1	1	1	4.36	711	8952
>sp|P23685|NAC1_CANLF Sodium/calcium exchanger 1 OS=Canis lupus familiaris OX=9615 GN=SLC8A1 PE=1 SV=1	0.13	0.09	151.4	18090485.6	3	1	0	1.03	970	764
>tr|F1PBU5|F1PBU5_CANLF Non-specific serine/threonine protein kinase OS=Canis lupus familiaris OX=9615 GN=SMG1 PE=3 SV=3	0.8	0.49	201.1	58165901.4	13	2	0	0.17	3634	6898
>tr|A0A5F4D6G2|A0A5F4D6G2_CANLF SMG7 nonsense mediated mRNA decay factor OS=Canis lupus familiaris OX=9615 GN=SMG7 PE=4 SV=1	0.76	0.76	235.5	5319387.2	1	1	0	0.43	1175	2075
>tr|A0A5F4CLU1|A0A5F4CLU1_CANLF Superoxide dismutase [Cu-Zn] OS=Canis lupus familiaris OX=9615 GN=SOD1 PE=3 SV=1	0.26	0.26	69.1	6925232.1	1	1	0	7.09	141	25417

>tr|A0A5K1V0D8|A0A5K1V0D8_CANLF Sulfatase 2 OS=Canis lupus familiaris OX=9615 GN=SULF2 PE=3 SV=1	0.29	0.13	207.9	37025926.2	9	1	0	0.35	859	1192
>tr|A0A5F4BNS6|A0A5F4BNS6_CANLF Transcription activation suppressor family member 2 OS=Canis lupus familiaris OX=9615 GN=TASOR2 PE=3 SV=1	0.19	0.17	57.8	11952164.7	2	1	0	0.7	2724	1014
>tr|F1PX00|F1PX00_CANLF Transmembrane and coiled-coil domain family 3 OS=Canis lupus familiaris OX=9615 GN=TMCC3 PE=3 SV=2	0.12	0.12	76.5	5051932	1	1	0	4.43	451	8778
>tr|E2RTL2|E2RTL2_CANLF Tubulin tyrosine ligase like 6 OS=Canis lupus familiaris OX=9615 GN=TTLL6 PE=4 SV=3	0.17	0.17	50	3278214.6	1	1	0	0.6	827	2703
>tr|A0A5F4BXB8|A0A5F4BXB8_CANLF Vacuolar protein sorting 13 homolog A OS=Canis lupus familiaris OX=9615 GN=VPS13A PE=3 SV=1	0.12	0.13	85.2	5464980.8	1	1	1	0.38	2859	6548
>tr|E2RCT1|E2RCT1_CANLF WAP domain-containing protein OS=Canis lupus familiaris OX=9615 PE=4 SV=2	3.06	1.64	242.5	74730589.2	12	2	0	9.48	116	21717
>tr|A0A5F4C981|A0A5F4C981_CANLF ADAM metallopeptidase domain 19 OS=Canis lupus familiaris OX=9615 GN=ADAM19 PE=4 SV=1	0.10	0.02	38.60	157728.0	1	1	1	0.33	914	44873
>sp|Q5TJG6|BRD2_CANLF Bromodomain-containing protein 2 OS=Canis lupus familiaris OX=9615 GN=BRD2 PE=3 SV=1	0.10	0.05	62.10	970280.1	1	1	0	0.50	803	400
>tr|A0A5F4BWF3|A0A5F4BWF3_CANLF Chromosome 7 C18orf25 homolog OS=Canis lupus familiaris OX=9615 GN=C7H18orf25 PE=4 SV=1	0.1	0.06	42.6	158519	1	1	0	1.43	350	32344
>tr|J9NSS6|J9NSS6_CANLF DNA helicase OS=Canis lupus familiaris OX=9615 GN=CHD2 PE=4 SV=2	0.15	0.1	65	3837333.6	4	1	0	0.28	1780	1264
>tr|F1P6D8|F1P6D8_CANLF Dynein axonemal heavy chain 5 OS=Canis lupus familiaris OX=9615 GN=DNAH5 PE=3 SV=3	0.56	0.16	86.5	40032442.8	21	1	1	0.15	4620	37739

>sp|O19179|GUC2D_CANLF Retinal guanylyl cyclase 1 OS=Canis lupus familiaris OX=9615 GN=GUCY2D PE=2 SV=1	0.1	0	30.3	8378400.5	5	1	0	0.27	1109	144
>tr|F1P721|F1P721_CANLF Kinase suppressor of ras 2 OS=Canis lupus familiaris OX=9615 GN=KSR2 PE=4 SV=3	0.52	0.16	58.6	42708176.2	19	1	0	0.53	950	2276
>tr|E2R902|E2R902_CANLF NIMA related kinase 10 OS=Canis lupus familiaris OX=9615 GN=NEK10 PE=4 SV=2	0.1	0.07	60.1	167669	1	1	0	0.43	1162	15134
>tr|A0A5F4BZ61|A0A5F4BZ61_CANLF G_PROTEIN_RECEP_F1_2 domain-containing protein OS=Canis lupus familiaris OX=9615 GN=OR5D13 PE=4 SV=1	0.1	0.03	33	914158.3	1	1	0	1.7	294	37537
>tr|E2RE16|E2RE16_CANLF Non-specific serine/threonine protein kinase OS=Canis lupus familiaris OX=9615 GN=PAK4 PE=4 SV=1	0.16	0.1	65	3098752.5	4	1	0	0.84	592	12735
>sp|P52212|PTHY_CANLF Parathyroid hormone OS=Canis lupus familiaris OX=9615 GN=PTH PE=3 SV=1	0.57	0.05	49.6	44022368.4	39	1	0	3.48	115	263
>tr|A0A5F4CCT2|A0A5F4CCT2_CANLF RNA binding motif protein 26 OS=Canis lupus familiaris OX=9615 GN=RBM26 PE=4 SV=1	0.1	0.05	50.8	172717	1	1	0	0.48	1035	7784
>tr|E2RKC5|E2RKC5_CANLF Rho family GTPase 2 OS=Canis lupus familiaris OX=9615 GN=RND2 PE=4 SV=1	0.13	0.03	32.3	17615279.1	6	1	0	1.75	229	1710
>sp|O46669|SCNAA_CANLF Sodium channel protein type 10 subunit alpha OS=Canis lupus familiaris OX=9615 GN=SCN10A PE=2 SV=1	0.14	0.04	58.2	2110332.3	6	2	0	0.25	1962	90
>tr|A0A5F4BY93|A0A5F4BY93_CANLF Serine and arginine rich splicing factor 5 OS=Canis lupus familiaris OX=9615 GN=SRSF5 PE=3 SV=1	0.1	0.06	52.2	169945.7	1	1	0	5	100	6726
>tr|F1PRU0|F1PRU0_CANLF WD_REPEATS_REGION domain-containing protein OS=Canis lupus familiaris OX=9615 GN=TLE7 PE=3 SV=2	0.52	0.16	58.6	42708176.2	19	1	0	1.16	431	22849

>tr|F1PX00|F1PX00_CANLF Transmembrane and coiled-coil domain family 3 OS=Canis lupus familiaris OX=9615 GN=TMCC3 PE=3 SV=2	0.1	0.02	41.6	6445273.1	6	1	0	1.11	451	8778
>sp|O18840|ACTB_CANLF Actin, cytoplasmic 1 OS=Canis lupus familiaris OX=9615 GN=ACTB PE=2 SV=3	4.97	4.18	383.1	113426229	19	2	0	5.33	375	642
>tr|J9NS28|J9NS28_CANLF RBR-type E3 ubiquitin transferase OS=Canis lupus familiaris OX=9615 GN=ANKIB1 PE=4 SV=2	0.13	0.07	35.6	15164556.5	4	1	0	2.33	988	26345
>sp|P62286|ASPM_CANLF Abnormal spindle-like microcephaly-associated protein homolog OS=Canis lupus familiaris OX=9615 GN=ASPM PE=2 SV=2	0.29	0.1	43.3	38400604.6	10	2	0	0.23	3469	677
>tr|Q9XSV4|Q9XSV4_CANLF CE10 protein OS=Canis lupus familiaris OX=9615 GN=ce10 PE=2 SV=1	0.58	0.42	225	14071262.5	7	2	0	9.09	110	41542
>sp|Q05052|OST48_CANLF Dolichyl-diphosphooligosaccharide--protein glycosyltransferase 48 kDa subunit OS=Canis lupus familiaris OX=9615 GN=DDOST PE=1 SV=1	0.91	0.89	72.1	4899209.3	2	1	0	2.47	445	254
>tr|A0A5F4CLN6|A0A5F4CLN6_CANLF DLG associated protein 4 OS=Canis lupus familiaris OX=9615 GN=DLGAP4 PE=3 SV=1	0.27	0.27	35.4	3800979.2	1	1	0	3.04	461	7940
>tr|E2RSI6|E2RSI6_CANLF Ezrin OS=Canis lupus familiaris OX=9615 GN=EZR PE=4 SV=1	0.41	0.41	111.4	3975779.6	1	1	0	1.54	586	15650
>tr|E2RM62|E2RM62_CANLF FAT atypical cadherin 2 OS=Canis lupus familiaris OX=9615 GN=FAT2 PE=4 SV=1	0.23	0.23	65	3771599.7	1	1	0	0.34	4354	8419
>tr|J9P432|J9P432_CANLF Glutamine--fructose-6-phosphate transaminase (isomerizing) OS=Canis lupus familiaris OX=9615 GN=GFPT1 PE=4 SV=2	0.33	0.29	58.2	5884831.8	3	1	0	1.18	677	7191
>tr|F1P8S0|F1P8S0_CANLF GPRIN family member 3 OS=Canis lupus familiaris OX=9615 GN=GPRIN3 PE=4 SV=2	0.39	0.4	55.7	4261456.3	1	1	0	1.42	776	30471

>tr|E2R264|E2R264_CANLF HAUS augmin like complex subunit 8 OS=Canis lupus familiaris OX=9615 GN=HAUS8 PE=4 SV=2	0.3	0.3	96.8	4797075.3	1	1	0	2.93	376	14074
>tr|H9GWH3|H9GWH3_CANLF LRRC37AB_C domain-containing protein OS=Canis lupus familiaris OX=9615 GN=LOC491436 PE=4 SV=3	0.24	0.24	137.3	2675078.4	1	1	0	0.75	796	5712
>tr|A0A5F4BVF3|A0A5F4BVF3_CANLF Lactotransferrin OS=Canis lupus familiaris OX=9615 GN=LTF PE=3 SV=1	2.97	0.77	245.2	85106957.9	21	8	0	12.78	626	32850
>tr|A0A5F4C2W9|A0A5F4C2W9_CANLF Methyl-CpG binding domain protein 1 OS=Canis lupus familiaris OX=9615 GN=MBD1 PE=4 SV=1	0.26	0.26	114.5	3212872.2	1	1	0	1.71	703	14519
>sp|Q28895|NPC2_CANLF NPC intracellular cholesterol transporter 2 OS=Canis lupus familiaris OX=9615 GN=NPC2 PE=2 SV=1	0.7	0.64	172.6	19345909.6	4	1	0	15.44	149	153
>tr|F1PYS8|F1PYS8_CANLF Olfactory receptor OS=Canis lupus familiaris OX=9615 GN=OR5P6 PE=3 SV=3	0.19	0.19	65	4442480.3	1	1	1	6.69	314	20945
>tr|A0A5F4D7Y5|A0A5F4D7Y5_CANLF Pleckstrin homology, MyTH4 and FERM domain containing H1 OS=Canis lupus familiaris OX=9615 GN=PLEKHH1 PE=4 SV=1	0.26	0.22	86.5	7286984.7	3	1	0	0.3	1342	5979
>tr|E2QWB2|E2QWB2_CANLF Plastin 1 OS=Canis lupus familiaris OX=9615 GN=PLS1 PE=4 SV=2	0.41	0.39	134.2	8639749.5	2	1	0	1.91	629	25458
>tr|A0A5F4D0F5|A0A5F4D0F5_CANLF Ring finger protein 24 OS=Canis lupus familiaris OX=9615 GN=RNF24 PE=4 SV=1	0.39	0.39	74.5	4122591	1	1	0	7.64	144	7273
>sp|P23685|NAC1_CANLF Sodium/calcium exchanger 1 OS=Canis lupus familiaris OX=9615 GN=SLC8A1 PE=1 SV=1	0.26	0.27	73.9	4423626.8	1	1	0	1.03	970	764

>tr|A0A5F4CLU1|A0A5F4CLU1_CANLF Superoxide dismutase [Cu-Zn] OS=Canis lupus familiaris OX=9615 GN=SOD1 PE=3 SV=1	0.4	0.4	79.2	2410644.2	1	1	0	7.09	141	25417
>tr|A0A5F4CFJ7|A0A5F4CFJ7_CANLF A-kinase anchoring protein 9 OS=Canis lupus familiaris OX=9615 GN=AKAP9 PE=4 SV=1	0.1	0.02	119.4	33642546.1	2	1	1	0.19	3721	9720
>sp|Q9TSZ6|DAG1_CANLF Dystroglycan OS=Canis lupus familiaris OX=9615 GN=DAG1 PE=3 SV=1	0.13	0.02	66.6	99809474.4	12	2	0	1.12	892	611
>tr|J9P0A6|J9P0A6_CANLF Developmentally regulated GTP binding protein 1 OS=Canis lupus familiaris OX=9615 GN=DRG1 PE=4 SV=1	0.1	0.02	37.4	5397138.4	1	1	1	1.63	367	9802
>tr|A0A5F4DC63|A0A5F4DC63_CANLF Transcription elongation factor 1 homolog OS=Canis lupus familiaris OX=9615 GN=ELOF1 PE=3 SV=1	0.1	0.02	52.7	59893366.4	4	1	1	7.23	83	29575
>tr|F6UXI8|F6UXI8_CANLF Inosine-5'-monophosphate dehydrogenase OS=Canis lupus familiaris OX=9615 GN=IMPDH2 PE=3 SV=2	0.1	0.02	39	6735843.2	1	1	0	0.97	514	12628
>tr|A0A5F4D3U8|A0A5F4D3U8_CANLF Multiple C2 and transmembrane domain containing 1 OS=Canis lupus familiaris OX=9615 GN=MCTP1 PE=4 SV=1	0.1	0.02	142.7	3643695.3	1	1	0	1.62	679	45081
>tr|A0A5F4D4W9|A0A5F4D4W9_CANLF Nucleoporin 210 like OS=Canis lupus familiaris OX=9615 GN=NUP210L PE=3 SV=1	0.1	0.02	77.9	33438578.5	2	1	1	0.46	1736	31558
>tr|E2RNR9|E2RNR9_CANLF Osteomodulin OS=Canis lupus familiaris OX=9615 GN=OMD PE=4 SV=3	0.1	0.02	56.4	16579777.3	1	1	1	1.69	414	32378
>tr|F1PPZ3|F1PPZ3_CANLF Olfactory receptor OS=Canis lupus familiaris OX=9615 GN=OR6S1 PE=3 SV=2	0.1	0.01	47.2	131347352	13	1	1	1.21	331	30247

>tr|A0A5F4CYK1|A0A5F4CYK1_CANLF 1-phosphatidylinositol 4,5-bisphosphate phosphodiesterase gamma OS=Canis lupus familiaris OX=9615 GN=PLCG2 PE=4 SV=1	0.1	0.02	101.8	16090283.8	2	1	1	0.65	1235	30558
>tr|A0A5F4DFY1|A0A5F4DFY1_CANLF SHH signaling and ciliogenesis regulator SDCCAG8 OS=Canis lupus familiaris OX=9615 GN=SDCCAG8 PE=4 SV=1	0.1	0	40.7	2108881.3	1	1	1	0.59	673	17765
>tr|E2RL65|E2RL65_CANLF Splicing factor 3b subunit 2 OS=Canis lupus familiaris OX=9615 GN=SF3B2 PE=4 SV=2	0.1	0.02	68.6	60952722.1	4	1	1	0.67	895	33110
>tr|F1PTH8|F1PTH8_CANLF Secreted frizzled related protein 5 OS=Canis lupus familiaris OX=9615 GN=SFRP5 PE=3 SV=2	0.1	0.02	44.1	24970147.2	2	1	1	5.59	179	42558
>tr|E2QRT5|E2QRT5_CANLF Structural maintenance of chromosomes protein OS=Canis lupus familiaris OX=9615 GN=SMC1B PE=3 SV=1	0.1	0.02	89	34505894.1	2	1	1	0.65	1235	2380
>tr|A0A5F4DEC9|A0A5F4DEC9_CANLF Telomerase reverse transcriptase OS=Canis lupus familiaris OX=9615 GN=TERT PE=3 SV=1	0.1	0.02	82.9	16257348.9	1	1	1	0.66	1215	11298
>tr|A0A5F4BY10|A0A5F4BY10_CANLF Palmitoyltransferase OS=Canis lupus familiaris OX=9615 GN=ZDHHC9 PE=3 SV=1	0.1	0.02	44.6	13303508.3	2	1	0	1.64	365	28149
>tr|A0A5F4D9K1|A0A5F4D9K1_CANLF Zinc finger protein 385D OS=Canis lupus familiaris OX=9615 GN=ZNF385D PE=4 SV=1	0.1	0.02	68.8	8164123.9	1	1	1	2.22	360	17255
>tr|F1PQN7|F1PQN7_CANLF Ankyrin repeat and BTB domain containing 2 OS=Canis lupus familiaris OX=9615 GN=ABTB2 PE=4 SV=2	0.18	0.16	133.2	15514641	2	1	0	0.67	1188	42888
>sp|O18840|ACTB_CANLF Actin, cytoplasmic 1 OS=Canis lupus familiaris OX=9615 GN=ACTB PE=2 SV=3	4.27	3.7	334.8	137279041	13	2	0	4.53	375	642

>sp|P49822|ALBU_CANLF Albumin OS=Canis lupus familiaris OX=9615 GN=ALB PE=1 SV=3	9.81	3.92	287.8	12969291.6	13	6	1	11.51	608	490
>sp|P62286|ASPM_CANLF Abnormal spindle-like microcephaly-associated protein homolog OS=Canis lupus familiaris OX=9615 GN=ASPM PE=2 SV=2	0.33	0.13	114.8	45431255.6	17	2	0	0.23	3469	677
>tr|E2QWH7|E2QWH7_CANLF Coiled-coil domain containing 127 OS=Canis lupus familiaris OX=9615 GN=CCDC127 PE=4 SV=3	0.1	0.09	66.8	7137123.7	1	1	0	3.34	479	28521
>tr|A0A5F4D7J3|A0A5F4D7J3_CANLF Non-specific serine/threonine protein kinase OS=Canis lupus familiaris OX=9615 GN=CDC42BPA PE=3 SV=1	0.1	0.02	61.1	7884672.7	3	1	1	0.17	1794	1069
>tr|Q9XSV4|Q9XSV4_CANLF CE10 protein OS=Canis lupus familiaris OX=9615 GN=ce10 PE=2 SV=1	2.59	1.77	249.1	24116860.5	12	3	0	14.55	110	41542
>tr|A0A5F4D9S5|A0A5F4D9S5_CANLF Hyaluronoglucosaminidase OS=Canis lupus familiaris OX=9615 GN=CEMIP PE=3 SV=1	0.44	0.42	140.8	5517350.8	2	1	0	0.24	1684	9775
>tr|A0A5F4C5M9|A0A5F4C5M9_CANLF Contactin associated protein 1 OS=Canis lupus familiaris OX=9615 GN=CNTNAP1 PE=3 SV=1	0.31	0.31	44	288154.4	1	1	0	0.36	1388	10827
>tr|A0A5F4CCD0|A0A5F4CCD0_CANLF Cysteine rich secretory protein 2 OS=Canis lupus familiaris OX=9615 GN=CRISP2 PE=3 SV=1	1.67	1.67	175	703679.8	1	1	0	4.82	311	11017
>tr|F1PCL0|F1PCL0_CANLF DNL-type zinc finger OS=Canis lupus familiaris OX=9615 GN=DNLZ PE=4 SV=2	0.1	0.05	104.9	10584036.3	2	2	2	5.26	171	2400
>tr|A0A5F4D4W8|A0A5F4D4W8_CANLF Dedicator of cytokinesis 1 OS=Canis lupus familiaris OX=9615 GN=DOCK1 PE=3 SV=1	0.1	0.09	69.4	4734497.8	1	1	1	1.02	1855	11311
>sp|Q9GL25|ESPB1_CANLF Epididymal sperm-binding protein 1 OS=Canis lupus familiaris OX=9615 GN=ELSPBP1 PE=1 SV=1	1.64	1.6	214.7	944907.5	3	1	0	4.49	245	36

>tr|A0A5F4DMP2|A0A5F4DMP2_CANLF Heterogeneous nuclear ribonucleoprotein M OS=Canis lupus familiaris OX=9615 GN=HNRNPM PE=4 SV=1	0.53	0.39	102.8	41232650.6	8	1	0	0.55	731	1228
>tr|F1PQ45|F1PQ45_CANLF Integrin subunit alpha 11 OS=Canis lupus familiaris OX=9615 GN=ITGA11 PE=3 SV=3	0.12	0.12	68.8	4113875.1	1	1	1	1.52	1183	26268
>tr|J9NS29|J9NS29_CANLF Cystatin domain-containing protein OS=Canis lupus familiaris OX=9615 GN=LOC607874 PE=4 SV=2	0.21	0.21	111	5867607.9	1	1	0	6.39	313	30016
>tr|F6XN72|F6XN72_CANLF Leucine rich repeat containing 71 OS=Canis lupus familiaris OX=9615 GN=LRRC71 PE=4 SV=1	0.1	0.06	105.2	5739592.9	1	1	1	1.96	560	34087
>tr|F1PR54|F1PR54_CANLF Lactotransferrin OS=Canis lupus familiaris OX=9615 GN=LTF PE=3 SV=1	18.92	3.64	313.9	109770182	47	12	0	15.96	708	40436
>tr|A0A5F4CR67|A0A5F4CR67_CANLF Methyl-CpG-binding protein 2 OS=Canis lupus familiaris OX=9615 GN=MECP2 PE=4 SV=1	0.1	0.09	126.1	6188853.7	1	1	1	2.41	498	1334
>tr|F1PFP6|F1PFP6_CANLF Matrix metallopeptidase 12 OS=Canis lupus familiaris OX=9615 GN=MMP12 PE=3 SV=3	0.3	0.28	63.7	12011017	2	1	0	2.44	491	24324
>tr|E2RJF6|E2RJF6_CANLF Mortality factor 4 like 1 OS=Canis lupus familiaris OX=9615 GN=MORF4L1 PE=4 SV=3	0.15	0.13	56	18084682.8	2	1	0	4.56	439	18584
>tr|F1PHA9|F1PHA9_CANLF Motile sperm domain containing 2 OS=Canis lupus familiaris OX=9615 GN=MOSPD2 PE=4 SV=3	0.33	0.31	158.3	11391947.3	2	1	0	0.97	518	10201
>tr|A0A5F4BQ68|A0A5F4BQ68_CANLF Interferon-induced GTP-binding protein Mx1 OS=Canis lupus familiaris OX=9615 GN=MX2 PE=3 SV=1	0.1	0.06	102.5	19008940.4	3	2	2	2.47	729	13816

>sp|F1PRN2|MYO1D_CANLF Unconventional myosin-Id OS=Canis lupus familiaris OX=9615 GN=MYO1D PE=1 SV=2	0.65	0.65	112	3386784.9	1	1	0	0.3	1006	763
>sp|Q28895|NPC2_CANLF NPC intracellular cholesterol transporter 2 OS=Canis lupus familiaris OX=9615 GN=NPC2 PE=2 SV=1	6.55	2.18	337.1	22906105.3	14	4	0	38.93	149	153
>tr|E2RRE4|E2RRE4_CANLF Netrin 5 OS=Canis lupus familiaris OX=9615 GN=NTN5 PE=4 SV=3	0.25	0.13	133.9	35168475.2	7	1	0	1.02	489	13410
>tr|A0A5F4C7X6|A0A5F4C7X6_CANLF Nucleoporin 58 OS=Canis lupus familiaris OX=9615 GN=NUP58 PE=4 SV=1	0.1	0.09	34.1	9211896.3	1	1	1	3.12	544	8520
>tr|F6Y091|F6Y091_CANLF Olfactory receptor OS=Canis lupus familiaris OX=9615 GN=OR13C2 PE=3 SV=1	0.1	0.01	31.1	10029599.6	8	1	1	1.26	318	2117
>tr|F1PL54|F1PL54_CANLF Olfactory receptor OS=Canis lupus familiaris OX=9615 GN=OR52E2B PE=3 SV=2	0.1	0.03	78.8	15785972.3	2	1	1	2.25	311	5515
>tr|F1PZN6|F1PZN6_CANLF Olfactory receptor OS=Canis lupus familiaris OX=9615 PE=3 SV=3	0.13	0.13	48.6	4598782	1	1	1	6.77	310	37737
>sp|P79149|PININ_CANLF Pinin OS=Canis lupus familiaris OX=9615 GN=PNN PE=2 SV=3	0.37	0.37	64.1	428019.6	1	1	0	0.65	773	156
>sp|Q9XS65|PTGDS_CANLF Prostaglandin-H2 D-isomerase OS=Canis lupus familiaris OX=9615 GN=PTGDS PE=2 SV=1	1.04	0.72	234.2	3595633.3	7	2	1	10.47	191	165
>tr|A0A5F4BY19|A0A5F4BY19_CANLF RELT like 1 OS=Canis lupus familiaris OX=9615 GN=RELL1 PE=3 SV=1	0.2	0.18	158.5	2270381.7	2	1	1	4.65	258	11917
>sp|E2RKA8|RL32_CANLF 60S ribosomal protein L32 OS=Canis lupus familiaris OX=9615 GN=RPL32 PE=1 SV=1	0.29	0.03	110.2	41596467.8	17	1	0	2.22	135	275
>tr|E2RS58|E2RS58_CANLF Solute carrier family 35 member B1 OS=Canis lupus familiaris OX=9615 GN=SLC35B1 PE=3 SV=2	0.1	0.05	64.7	2610967.5	1	1	1	0.83	360	7421

>tr|F1PY73|F1PY73_CANLF SMG6 nonsense mediated mRNA decay factor OS=Canis lupus familiaris OX=9615 GN=SMG6 PE=4 SV=2	0.34	0.34	82.7	6024611.4	1	1	0	1.42	1412	40090
>tr|J9P0B4|J9P0B4_CANLF Tudor domain containing 15 OS=Canis lupus familiaris OX=9615 GN=TDRD15 PE=4 SV=2	0.67	0.65	153.1	1026905.3	2	1	0	0.57	2105	4188
>tr|J9P539|J9P539_CANLF Telomeric repeat-binding factor OS=Canis lupus familiaris OX=9615 GN=TERF1 PE=4 SV=2	0.1	0.07	98.6	5673217.6	1	1	1	2.66	413	7133
>tr|J9NT31|J9NT31_CANLF Thymocyte selection associated family member 2 OS=Canis lupus familiaris OX=9615 GN=THEMIS2 PE=3 SV=1	0.53	0.39	42.5	10171395	8	1	0	0.78	642	9120
>sp|Q697L1|TRPV1_CANLF Transient receptor potential cation channel subfamily V member 1 OS=Canis lupus familiaris OX=9615 GN=TRPV1 PE=2 SV=1	2.18	0.16	115.7	587367199	118	3	2	0.71	840	297
>tr|A0A5F4DM58|A0A5F4DM58_CANLF Zinc finger MYM-type containing 2 OS=Canis lupus familiaris OX=9615 GN=ZMYM2 PE=4 SV=1	0.13	0.09	98.2	23746174.5	3	1	1	0.85	1295	4956
>tr|E2R824|E2R824_CANLF Zinc finger protein 518B OS=Canis lupus familiaris OX=9615 GN=ZNF518B PE=4 SV=3	0.14	0.14	92.9	4810757.6	1	1	0	0.8	1000	24468
>sp|Q6UR05|MRP1_CANLF Multidrug resistance-associated protein 1 OS=Canis lupus familiaris OX=9615 GN=ABCC1 PE=1 SV=1	0.24	0.2	115.8	6036770.1	3	1	0	0.13	1531	259
>tr|A0A5F4DFU6|A0A5F4DFU6_CANLF Acetyl-CoA acyltransferase 1 OS=Canis lupus familiaris OX=9615 GN=ACAA1 PE=3 SV=1	0.13	0.11	217.7	4646285	2	1	0	0.97	513	2936
>sp|O18840|ACTB_CANLF Actin, cytoplasmic 1 OS=Canis lupus familiaris OX=9615 GN=ACTB PE=2 SV=3	0.3	0.24	150	6127412.6	4	1	0	2.93	375	642

>sp|P49822|ALBU_CANLF Albumin OS=Canis lupus familiaris OX=9615 GN=ALB PE=1 SV=3	3.86	2.52	211.1	188982271	22	4	0	8.39	608	490
>tr|J9P2D3|J9P2D3_CANLF Alpha kinase 3 OS=Canis lupus familiaris OX=9615 GN=ALPK3 PE=4 SV=2	0.32	0.24	190.3	11794790.9	5	1	0	0.31	1616	5104
>tr|E2R4W0|E2R4W0_CANLF Anaphase-promoting complex subunit 11 OS=Canis lupus familiaris OX=9615 GN=ANAPC11 PE=3 SV=2	0.25	0.23	158.9	16436251.7	2	1	0	1.75	458	23846
>tr|F1PI09|F1PI09_CANLF Aldehyde oxidase OS=Canis lupus familiaris OX=9615 GN=AOX2 PE=3 SV=3	0.37	0.36	180.3	1428729.9	2	1	0	0.67	1347	21650
>tr|F1PGF9|F1PGF9_CANLF Rho guanine nucleotide exchange factor 26 OS=Canis lupus familiaris OX=9615 GN=ARHGEF26 PE=4 SV=3	0.74	0.7	304.4	34821887.7	3	1	0	0.84	594	22876
>tr|J9P3H8|J9P3H8_CANLF ATM interactor OS=Canis lupus familiaris OX=9615 GN=ATMIN PE=4 SV=2	0.48	0.02	40.6	20200389.4	24	1	0	0.58	863	882
>tr|A0A5F4C1S8|A0A5F4C1S8_CANLF E3 ubiquitin-protein ligase CBL OS=Canis lupus familiaris OX=9615 GN=CBL PE=4 SV=1	0.5	0.08	153.6	151951513	21	2	0	0.91	773	1308
>tr|A0A5F4D7J3|A0A5F4D7J3_CANLF Non-specific serine/threonine protein kinase OS=Canis lupus familiaris OX=9615 GN=CDC42BPA PE=3 SV=1	1.12	1.03	283.4	37440878	6	1	0	0.22	1794	1069
>tr|Q9XSV4|Q9XSV4_CANLF CE10 protein OS=Canis lupus familiaris OX=9615 GN=ce10 PE=2 SV=1	1.24	0.87	210.7	14879128.5	7	2	0	11.82	110	41542
>tr|A0A5F4D9S5|A0A5F4D9S5_CANLF Hyaluronoglucosaminidase OS=Canis lupus familiaris OX=9615 GN=CEMIP PE=3 SV=1	0.16	0.16	103.4	4195688.3	1	1	0	0.24	1684	9775
>tr|J9NSS6|J9NSS6_CANLF DNA helicase OS=Canis lupus familiaris OX=9615 GN=CHD2 PE=4 SV=2	0.14	0.1	57.5	1031603	3	1	0	0.28	1780	1264
>tr|A0A5F4BRN6|A0A5F4BRN6_CANLF Class II major histocompatibility complex transactivator OS=Canis lupus familiaris OX=9615 GN=CIITA PE=4 SV=1	0.6	0.6	179.4	5590030.8	1	1	0	0.34	1191	5452

>sp|P25473|CLUS_CANLF Clusterin OS=Canis lupus familiaris OX=9615 GN=CLU PE=2 SV=1	5.63	2.22	328.3	211355224	32	7	0	8.99	445	725
>sp|P21842|CMA1_CANLF Chymase OS=Canis lupus familiaris OX=9615 GN=CMA1 PE=1 SV=1	0.45	0.41	92.6	14291715.1	3	1	0	0.8	249	34
>tr|A0A5F4CCD0|A0A5F4CCD0_CANLF Cysteine rich secretory protein 2 OS=Canis lupus familiaris OX=9615 GN=CRISP2 PE=3 SV=1	0.67	0.63	190	15676538.4	3	1	0	2.25	311	11017
>sp|Q5TJE1|DAXX_CANLF Death domain-associated protein 6 OS=Canis lupus familiaris OX=9615 GN=DAXX PE=3 SV=1	0.46	0.44	155.7	10620387.5	2	1	0	0.41	737	429
>tr|J9NYC7|J9NYC7_CANLF Dynein axonemal heavy chain 12 OS=Canis lupus familiaris OX=9615 GN=DNAH12 PE=3 SV=1	0.35	0.32	134.3	503314.2	3	1	0	0.33	3960	15992
>tr|F1P9L4|F1P9L4_CANLF Dual-specificity kinase OS=Canis lupus familiaris OX=9615 GN=DYRK1A PE=3 SV=3	0.44	0.44	88.9	5578141.7	1	1	0	0.52	762	4349
>sp|Q9GL25|ESPB1_CANLF Epididymal sperm-binding protein 1 OS=Canis lupus familiaris OX=9615 GN=ELSPBP1 PE=1 SV=1	3.55	2.26	137.4	32822569.4	5	2	0	11.43	245	36
>tr|F1PJY1|F1PJY1_CANLF Mannosyl-glycoprotein endo-beta-N-acetylglucosaminidase OS=Canis lupus familiaris OX=9615 GN=ENGASE PE=3 SV=3	0.7	0.25	176.2	15431288.8	16	2	1	1.74	690	32761
>tr|F1PPP9|F1PPP9_CANLF Family with sequence similarity 135 member A OS=Canis lupus familiaris OX=9615 GN=FAM135A PE=3 SV=3	0.33	0.1	169.8	16443805.1	16	1	0	1.22	1399	6815
>tr|A0A5F4DGS3|A0A5F4DGS3_CANLF Polypeptide N-acetylgalactosaminyltransferase OS=Canis lupus familiaris OX=9615 GN=GALNT18 PE=3 SV=1	0.35	0.33	101.5	21316920.7	2	1	0	0.99	607	14798
>tr|A0A5F4CJ52|A0A5F4CJ52_CANLF Beta-galactosidase OS=Canis lupus familiaris OX=9615 GN=GLB1 PE=3 SV=1	0.23	0.23	99.9	4705074.6	1	1	0	0.53	946	3892

>tr|F1PJ71|F1PJ71_CANLF Glutathione peroxidase OS=Canis lupus familiaris OX=9615 GN=GPX5 PE=3 SV=2	11.93	3.79	293.4	558589605	68	8	1	30.32	221	19009
>tr|A0A5F4CLI1|A0A5F4CLI1_CANLF Histone deacetylase 6 OS=Canis lupus familiaris OX=9615 GN=HDAC6 PE=4 SV=1	0.63	0.63	250.7	4526077.8	1	1	0	0.43	1175	4057
>tr|E2QW13|E2QW13_CANLF Inhibin subunit beta A OS=Canis lupus familiaris OX=9615 GN=INHBA PE=3 SV=1	0.54	0.48	70.5	34234131.7	4	1	1	2.59	424	5794
>tr|A0A5F4BZ12|A0A5F4BZ12_CANLF Importin 11 OS=Canis lupus familiaris OX=9615 GN=IPO11 PE=4 SV=1	1.21	1.17	268.9	26980253.9	3	1	0	0.72	968	20170
>tr|E2R0S2|E2R0S2_CANLF [Histone H3]-trimethyl-L-lysine(4) demethylase OS=Canis lupus familiaris OX=9615 GN=KDM5C PE=3 SV=3	0.54	0.53	105.7	16949610.2	2	1	0	0.46	1523	25191
>sp|Q30DN6|KDM5D_CANLF Lysine-specific demethylase 5D OS=Canis lupus familiaris OX=9615 GN=KDM5D PE=2 SV=1	0.35	0.35	93.4	1805107.1	1	1	0	0.13	1545	213
>tr|E2R6E0|E2R6E0_CANLF Lipocln_cytosolic_FA-bd_dom domain-containing protein OS=Canis lupus familiaris OX=9615 GN=LCNL1 PE=3 SV=2	3.03	2.97	193.1	30089137	4	1	0	3.01	299	1932
>tr|F1PG90|F1PG90_CANLF Leucine rich repeat LGI family member 3 OS=Canis lupus familiaris OX=9615 GN=LGI3 PE=4 SV=3	0.1	0.07	74	5562585.2	1	1	0	0.73	548	1276
>tr|E2R0G7|E2R0G7_CANLF DNA ligase OS=Canis lupus familiaris OX=9615 GN=LIG4 PE=3 SV=1	0.14	0.1	45.3	15723919.3	3	1	0	0.55	911	29979
>tr|E2RMC9|E2RMC9_CANLF DUF4515 domain-containing protein OS=Canis lupus familiaris OX=9615 GN=LOC100688167 PE=4 SV=3	0.15	0.04	182	36108884.8	11	2	0	2.08	432	29175
>tr|J9NZH4|J9NZH4_CANLF NTR domain-containing protein OS=Canis lupus familiaris OX=9615 GN=LOC102154527 PE=3 SV=2	0.1	0.1	53.1	6165021.7	1	1	0	8.6	221	39505

>tr|J9NS29|J9NS29_CANLF Cystatin domain-containing protein OS=Canis lupus familiaris OX=9615 GN=LOC607874 PE=4 SV=2	0.32	0.28	111.9	30625104.5	3	1	0	4.79	313	30016
>tr|E2R9Z9|E2R9Z9_CANLF Leucine rich repeat containing 8 VRAC subunit C OS=Canis lupus familiaris OX=9615 GN=LRRC8C PE=3 SV=1	0.12	0.12	65.7	651475.4	1	1	0	0.62	803	9247
>tr|F1PR54|F1PR54_CANLF Lactotransferrin OS=Canis lupus familiaris OX=9615 GN=LTF PE=3 SV=1	23.56	4.15	274.1	575486227	90	14	1	22.03	708	40436
>tr|E2RN16|E2RN16_CANLF Mitogen-activated protein kinase kinase kinase 2 OS=Canis lupus familiaris OX=9615 GN=MAP3K2 PE=4 SV=2	0.26	0.1	219.1	106763901	10	1	0	0.97	620	34325
>tr|F1PR47|F1PR47_CANLF DNA replication licensing factor MCM2 OS=Canis lupus familiaris OX=9615 GN=MCM2 PE=3 SV=3	0.28	0.24	196.5	8440132.5	3	1	0	0.57	881	9370
>tr|F1PFZ5|F1PFZ5_CANLF Milk fat globule EGF and factor V/VIII domain containing OS=Canis lupus familiaris OX=9615 GN=MFGE8 PE=4 SV=3	0.49	0.43	157.4	31552216.5	4	1	0	1.87	428	7079
>tr|J9P4U7|J9P4U7_CANLF Alpha-1,6-mannosyl-glycoprotein 6-beta-N-acetylglucosaminyltransferase OS=Canis lupus familiaris OX=9615 GN=MGAT5B PE=3 SV=2	0.17	0.13	65.9	30557673.3	3	1	1	1.39	792	13595
>tr|F6V8I0|F6V8I0_CANLF Melanophilin OS=Canis lupus familiaris OX=9615 GN=MLPH PE=4 SV=2	0.11	0.09	148.9	7616201.3	2	1	0	1.46	549	10015
>sp|Q28895|NPC2_CANLF NPC intracellular cholesterol transporter 2 OS=Canis lupus familiaris OX=9615 GN=NPC2 PE=2 SV=1	3.96	1.84	408.1	56541705.1	29	4	0	36.91	149	153
>tr|A0A5F4CQY7|A0A5F4CQY7_CANLF Neuregulin 2 OS=Canis lupus familiaris OX=9615 GN=NRG2 PE=4 SV=1	0.1	0.02	85.4	23042448.7	35	1	0	0.62	802	13353
>tr|F1PB68|F1PB68_CANLF Olfactomedin 4 OS=Canis lupus familiaris OX=9615 GN=OLFM4 PE=4 SV=3	0.23	0.19	92.4	16620344	3	1	0	4.4	477	17246

>tr|A0A5F4DD58|A0A5F4DD58_CANLF Phosphoinositide phospholipase C OS=Canis lupus familiaris OX=9615 GN=PLCD3 PE=4 SV=1	0.51	0.31	230.4	232039411	140	3	0	1.48	741	3088
>tr|F1PS80|F1PS80_CANLF Protein phosphatase 4 regulatory subunit 1 OS=Canis lupus familiaris OX=9615 GN=PPP4R1 PE=4 SV=3	0.11	0.07	50.3	23137086.9	3	1	0	0.56	887	2666
>sp|Q8WN22|PRKDC_CANLF DNA-dependent protein kinase catalytic subunit OS=Canis lupus familiaris OX=9615 GN=PRKDC PE=2 SV=1	1.9	1.18	185.4	33005844.1	6	3	0	0.22	4144	338
>tr|A0A5F4CEM0|A0A5F4CEM0_CANLF Proline rich coiled-coil 2B OS=Canis lupus familiaris OX=9615 GN=PRRC2B PE=4 SV=1	0.16	0.02	127.9	135370940	13	1	1	0.58	2235	37089
>sp|Q9XS65|PTGDS_CANLF Prostaglandin-H2 D-isomerase OS=Canis lupus familiaris OX=9615 GN=PTGDS PE=2 SV=1	5.28	2.2	294.9	434245415	55	6	1	24.08	191	165
>tr|F1PLT8|F1PLT8_CANLF Sulfhydryl oxidase OS=Canis lupus familiaris OX=9615 GN=QSOX1 PE=3 SV=3	0.15	0.13	141.5	9369012.1	2	1	0	2.46	568	33056
>tr|J9P3J0|J9P3J0_CANLF R3H domain and coiled-coil containing 1 OS=Canis lupus familiaris OX=9615 GN=R3HCC1 PE=4 SV=1	0.23	0.23	99.9	4705074.6	1	1	0	1.09	458	1410
>tr|E2RG76|E2RG76_CANLF Inactive ribonuclease-like protein 10 OS=Canis lupus familiaris OX=9615 GN=RNASE10 PE=3 SV=2	0.22	0.1	202.9	59813431	8	1	0	3.7	297	8212
>sp|Q9XSU7|RL27_CANLF 60S ribosomal protein L27 OS=Canis lupus familiaris OX=9615 GN=RPL27 PE=2 SV=3	0.1	0.03	46.7	9007219.1	10	1	0	3.68	136	314
>sp|Q28346|RL4_CANLF 60S ribosomal protein L4 OS=Canis lupus familiaris OX=9615 GN=RPL4 PE=1 SV=2	0.35	0.31	47.9	12659890.7	3	1	0	0.48	421	168
>tr|A0A5F4DCA4|A0A5F4DCA4_CANLF Reverse transcriptase domain-containing protein OS=Canis lupus familiaris OX=9615 PE=4 SV=1	0.32	0.3	125.5	10372354.2	2	1	0	0.31	978	860

>tr|A0A5F4DDH9|A0A5F4DDH9_CANLF Saccharopine dehydrogenase (putative) OS=Canis lupus familiaris OX=9615 GN=SCCPDH PE=4 SV=1	0.27	0.27	61.7	102848.6	1	1	0	3.09	421	6763
>tr|A0A5F4C730|A0A5F4C730_CANLF Semaphorin 4D OS=Canis lupus familiaris OX=9615 GN=SEMA4D PE=3 SV=1	0.35	0.31	202.4	26436220.2	3	1	0	0.28	1067	1802
>tr|A0A5F4D6G2|A0A5F4D6G2_CANLF SMG7 nonsense mediated mRNA decay factor OS=Canis lupus familiaris OX=9615 GN=SMG7 PE=4 SV=1	0.73	0.71	153.6	11313777.9	2	1	0	0.43	1175	2075
>tr|A0A5K1V0D8|A0A5K1V0D8_CANLF Sulfatase 2 OS=Canis lupus familiaris OX=9615 GN=SULF2 PE=3 SV=1	0.16	0.02	128.4	40089978	9	1	0	0.35	859	1192
>tr|A0A5F4C9T7|A0A5F4C9T7_CANLF Telomerase associated protein 1 OS=Canis lupus familiaris OX=9615 GN=TEP1 PE=4 SV=1	0.61	0.57	214.4	12659890.7	3	1	0	0.16	2507	891
>tr|F1PBJ1|F1PBJ1_CANLF Methylcytosine dioxygenase TET OS=Canis lupus familiaris OX=9615 GN=TET3 PE=3 SV=2	0.4	0.1	44.8	62836488.3	16	1	0	0.28	1795	1529
>tr|A0A5F4D9R8|A0A5F4D9R8_CANLF Transcription factor CP2 OS=Canis lupus familiaris OX=9615 GN=TFCP2 PE=3 SV=1	0.9	0.9	31.5	7139238.5	1	1	0	3.15	508	12674
>tr|F1Q432|F1Q432_CANLF Tenascin XB OS=Canis lupus familiaris OX=9615 GN=TNXB PE=3 SV=3	0.53	0.49	279.5	32357737.3	3	1	0	0.13	3966	30371
>tr|A0A5F4DKM7|A0A5F4DKM7_CANLF Terminal uridylyl transferase 4 OS=Canis lupus familiaris OX=9615 GN=TUT4 PE=4 SV=1	0.47	0.47	120.6	5294282.5	1	1	0	0.19	1611	941
>tr|A0A5F4C9R3|A0A5F4C9R3_CANLF Ubiquitin protein ligase E3 component n-recognin 5 OS=Canis lupus familiaris OX=9615 GN=UBR5 PE=4 SV=1	0.7	0.71	150	5770903.4	1	1	0	0.18	2714	3284
>tr|E2RCT1|E2RCT1_CANLF WAP domain-containing protein OS=Canis lupus familiaris OX=9615 PE=4 SV=2	0.62	0.28	173.6	27296972.7	6	3	0	24.14	116	21717

>tr|J9NZJ2|J9NZJ2_CANLF Protein Wnt OS=Canis lupus familiaris OX=9615 GN=WNT6 PE=3 SV=2	0.23	0.21	214.9	17561941.3	2	1	0	1.47	339	11134
>tr|E2R824|E2R824_CANLF Zinc finger protein 518B OS=Canis lupus familiaris OX=9615 GN=ZNF518B PE=4 SV=3	0.15	0.02	115.7	45048357.4	41	2	1	1.3	1000	24468
