# Supplementary material for: Potential Proteins Associated with Canine Epididymal Sperm Motility
Source: Cells. 2026 Jan 4;15(1):85. doi: 10.3390/cells15010085 (PMC12786130; doi:10.3390/cells15010085)
Supplement: Supplementary file 1 [file cells-15-00085-s001.zip › Supplementary Table S3.pdf]

Supplementary Table S3. The set of canine epididymal sperm (ES) proteins that constitute <1% in the good sperm motility (GSM) group.

Description	|Log Prob|	Best |Log Prob|	Best score	Total Intensity	# of spectra	# of unique peptides	# of mod peptides	Coverage %	# AA's in protein	Protein DB number
>sp|O18840|ACTB_CANLF Actin, cytoplasmic 1 OS=Canis lupus familiaris OX=9615 GN=ACTB PE=2 SV=3	2.1	1.13	223.6	1148831.5	6	3	0	12	375	642
>sp|O18840|ACTB_CANLF Actin, cytoplasmic 1 OS=Canis lupus familiaris OX=9615 GN=ACTB PE=2 SV=3	1.44	1.3	287.5	55329329.6	10	2	0	5.07	375	642
>sp|O18840|ACTB_CANLF Actin, cytoplasmic 1 OS=Canis lupus familiaris OX=9615 GN=ACTB PE=2 SV=3	2.55	1.19	236.4	90298869.2	14	3	0	9.87	375	642
>sp|O18840|ACTB_CANLF Actin, cytoplasmic 1 OS=Canis lupus familiaris OX=9615 GN=ACTB PE=2 SV=3	1.94	1.72	238.1	103375481	12	1	0	2.93	375	642
>sp|O18840|ACTB_CANLF Actin, cytoplasmic 1 OS=Canis lupus familiaris OX=9615 GN=ACTB PE=2 SV=3	4.29	3.13	343.4	208868607	18	2	0	5.33	375	642
>sp|O18840|ACTB_CANLF Actin, cytoplasmic 1 OS=Canis lupus familiaris OX=9615 GN=ACTB PE=2 SV=3	4.31	2.86	356.4	386319673	26	3	0	5.6	375	642
>sp|O18840|ACTB_CANLF Actin, cytoplasmic 1 OS=Canis lupus familiaris OX=9615 GN=ACTB PE=2 SV=3	8.05	3.92	296.4	390446921	21	4	0	13.07	375	642
>sp|O18840|ACTB_CANLF Actin, cytoplasmic 1 OS=Canis lupus familiaris OX=9615 GN=ACTB PE=2 SV=3	4.71	3.57	281.8	273998735	19	2	0	5.33	375	642

>sp|O18840|ACTB_CANLF Actin, cytoplasmic 1 OS=Canis lupus familiaris OX=9615 GN=ACTB PE=2 SV=3	5.33	3.46	321.2	111249282	15	3	0	5.6	375	642
>sp|O18840|ACTB_CANLF Actin, cytoplasmic 1 OS=Canis lupus familiaris OX=9615 GN=ACTB PE=2 SV=3	6.39	3.83	322.2	168494181	21	5	0	8.27	375	642
>tr|F1PGK9|F1PGK9_CANLF ADAM metallopeptidase with thrombospondin type 1 motif 5 OS=Canis lupus familiaris OX=9615 GN=ADAMTS5 PE=4 SV=3	0.12	0.02	75.8	41480860.2	27	1	0	0.59	845	11956
>tr|F1PGK9|F1PGK9_CANLF ADAM metallopeptidase with thrombospondin type 1 motif 5 OS=Canis lupus familiaris OX=9615 GN=ADAMTS5 PE=4 SV=3	0.34	0.15	105.9	23566169.6	12	1	0	0.59	845	11956
>tr|F1PGK9|F1PGK9_CANLF ADAM metallopeptidase with thrombospondin type 1 motif 5 OS=Canis lupus familiaris OX=9615 GN=ADAMTS5 PE=4 SV=3	0.29	0.02	62.8	71960179.4	31	1	0	0.59	845	11956
>tr|F1PGK9|F1PGK9_CANLF ADAM metallopeptidase with thrombospondin type 1 motif 5 OS=Canis lupus familiaris OX=9615 GN=ADAMTS5 PE=4 SV=3	0.23	0.02	35.6	65563484.4	32	1	0	0.59	845	11956
>tr|F2Z4Q6|F2Z4Q6_CANLF Alpha fetoprotein OS=Canis lupus familiaris OX=9615 GN=AFP PE=4 SV=2	20.91	4.01	308.9	651306681	130	17	2	33.28	637	24990
>sp|Q2PQH8|GDE_CANLF Glycogen debranching enzyme OS=Canis lupus familiaris OX=9615 GN=AGL PE=2 SV=1	0.42	0.09	104.8	92985336	15	2	0	0.26	1533	23
>sp|Q2PQH8|GDE_CANLF Glycogen debranching enzyme OS=Canis lupus familiaris OX=9615 GN=AGL PE=2 SV=1	0.98	0.39	106.2	60463153.8	13	5	0	0.65	1533	23
>sp|Q2PQH8|GDE_CANLF Glycogen debranching enzyme OS=Canis lupus familiaris OX=9615 GN=AGL PE=2 SV=1	0.7	0.34	78.1	168347768	13	2	0	0.26	1533	23

>sp|Q2PQH8|GDE_CANLF Glycogen debranching enzyme OS=Canis lupus familiaris OX=9615 GN=AGL PE=2 SV=1	2.49	2.28	112.9	79288050	5	2	0	0.26	1533	23
>sp|P49822|ALBU_CANLF Albumin OS=Canis lupus familiaris OX=9615 GN=ALB PE=1 SV=3	13.42	2.87	336.3	18325290.9	58	12	1	23.85	608	490
>sp|P49822|ALBU_CANLF Albumin OS=Canis lupus familiaris OX=9615 GN=ALB PE=1 SV=3	0.35	0.35	182.5	5910507.1	1	1	0	1.64	608	490
>sp|P49822|ALBU_CANLF Albumin OS=Canis lupus familiaris OX=9615 GN=ALB PE=1 SV=3	1.27	0.75	97.6	37265884.2	6	2	0	4.77	608	490
>sp|P49822|ALBU_CANLF Albumin OS=Canis lupus familiaris OX=9615 GN=ALB PE=1 SV=3	3.43	2.97	251.9	72775789.7	7	3	0	6.91	608	490
>sp|P49822|ALBU_CANLF Albumin OS=Canis lupus familiaris OX=9615 GN=ALB PE=1 SV=3	0.11	0.05	208.2	25738417	4	1	0	2.3	608	490
>sp|P49822|ALBU_CANLF Albumin OS=Canis lupus familiaris OX=9615 GN=ALB PE=1 SV=3	15.98	4.19	403.1	566233766	36	8	0	16.61	608	490
>sp|P49822|ALBU_CANLF Albumin OS=Canis lupus familiaris OX=9615 GN=ALB PE=1 SV=3	3.82	0.88	276.2	114292000	19	5	1	8.39	608	490
>tr|A0A5F4CHL0|A0A5F4CHL0_CANLF Anoctamin OS=Canis lupus familiaris OX=9615 GN=ANO9 PE=3 SV=1	0.65	0.39	176.5	302257152	14	1	0	1.82	824	37323
>tr|F1PGF9|F1PGF9_CANLF Rho guanine nucleotide exchange factor 26 OS=Canis lupus familiaris OX=9615 GN=ARHGEF26 PE=4 SV=3	1.39	1.29	320.3	87856755.1	6	1	0	0.84	594	22876
>tr|F1PGF9|F1PGF9_CANLF Rho guanine nucleotide exchange factor 26 OS=Canis lupus familiaris OX=9615 GN=ARHGEF26 PE=4 SV=3	2.09	2.05	312.3	95855749.2	4	2	1	2.36	594	22876
>sp|P62286|ASPM_CANLF Abnormal spindle-like microcephaly-associated protein homolog OS=Canis lupus familiaris OX=9615 GN=ASPM PE=2 SV=2	0.94	0.36	101.2	57685539.8	6	4	0	0.32	3469	677

>sp|P62286|ASPM_CANLF Abnormal spindle-like microcephaly-associated protein homolog OS=Canis lupus familiaris OX=9615 GN=ASPM PE=2 SV=2	1.18	1.1	94.1	258449449	29	2	0	0.14	3469	677
>sp|P62286|ASPM_CANLF Abnormal spindle-like microcephaly-associated protein homolog OS=Canis lupus familiaris OX=9615 GN=ASPM PE=2 SV=2	0.25	0.15	86.6	605260378	67	3	0	0.29	3469	677
>sp|P62286|ASPM_CANLF Abnormal spindle-like microcephaly-associated protein homolog OS=Canis lupus familiaris OX=9615 GN=ASPM PE=2 SV=2	1.96	0.14	85.2	287427351	90	3	0	0.32	3469	677
>sp|P62286|ASPM_CANLF Abnormal spindle-like microcephaly-associated protein homolog OS=Canis lupus familiaris OX=9615 GN=ASPM PE=2 SV=2	2.34	0.21	103	349492155	94	3	0	0.23	3469	677
>tr|E2R5H9|E2R5H9_CANLF Blood vessel epicardial substance OS=Canis lupus familiaris OX=9615 GN=BVES PE=3 SV=1	0.4	0.13	63.2	353287638	15	1	0	1.67	360	8594
>tr|A0A5F4CR89|A0A5F4CR89_CANLF Voltage-dependent R-type calcium channel subunit alpha OS=Canis lupus familiaris OX=9615 GN=CACNA1E PE=3 SV=1	0.1	0.01	58.2	195241904	11	1	0	0.19	2688	1145
>tr|A0A5F4C1S8|A0A5F4C1S8_CANLF E3 ubiquitin-protein ligase CBL OS=Canis lupus familiaris OX=9615 GN=CBL PE=4 SV=1	1.5	0.48	198.4	330371189	50	2	0	0.91	773	1308
>tr|Q9XSV4|Q9XSV4_CANLF CE10 protein OS=Canis lupus familiaris OX=9615 GN=ce10 PE=2 SV=1	5.37	3.36	320.2	223705143	44	3	0	12.73	110	41542
>tr|Q9XSV4|Q9XSV4_CANLF CE10 protein OS=Canis lupus familiaris OX=9615 GN=ce10 PE=2 SV=1	2.93	1.9	312.3	22146957.7	9	2	0	9.09	110	41542
>tr|Q9XSV4|Q9XSV4_CANLF CE10 protein OS=Canis lupus familiaris OX=9615 GN=ce10 PE=2 SV=1	5.67	3.77	288	205217653	30	3	0	12.73	110	41542
>tr|Q9XSV4|Q9XSV4_CANLF CE10 protein OS=Canis lupus familiaris OX=9615 GN=ce10 PE=2 SV=1	1.04	0.4	187.2	47663466.3	15	3	0	12.73	110	41542

>tr|Q9XSV4|Q9XSV4_CANLF CE10 protein OS=Canis lupus familiaris OX=9615 GN=ce10 PE=2 SV=1	1.99	1.83	273.7	27127890.7	7	2	0	9.09	110	41542
>tr|Q9XSV4|Q9XSV4_CANLF CE10 protein OS=Canis lupus familiaris OX=9615 GN=ce10 PE=2 SV=1	5.15	3.09	306.7	138867228	28	2	0	9.09	110	41542
>tr|Q9XSV4|Q9XSV4_CANLF CE10 protein OS=Canis lupus familiaris OX=9615 GN=ce10 PE=2 SV=1	3.82	3.16	309.9	197948111	37	2	0	9.09	110	41542
>tr|Q9XSV4|Q9XSV4_CANLF CE10 protein OS=Canis lupus familiaris OX=9615 GN=ce10 PE=2 SV=1	3.28	2.61	253.3	117020950	19	3	0	14.55	110	41542
>tr|Q9XSV4|Q9XSV4_CANLF CE10 protein OS=Canis lupus familiaris OX=9615 GN=ce10 PE=2 SV=1	3.86	1.63	281.4	38452000.8	16	3	0	14.55	110	41542
>tr|Q9XSV4|Q9XSV4_CANLF CE10 protein OS=Canis lupus familiaris OX=9615 GN=ce10 PE=2 SV=1	7.57	3.06	363.8	74770867.6	31	4	0	23.64	110	41542
>tr|A0A5F4D9S5|A0A5F4D9S5_CANLF Hyaluronoglucosaminidase OS=Canis lupus familiaris OX=9615 GN=CEMIP PE=3 SV=1	0.48	0.46	220.7	13297319.1	2	1	0	0.24	1684	9775
>tr|A0A5F4D9S5|A0A5F4D9S5_CANLF Hyaluronoglucosaminidase OS=Canis lupus familiaris OX=9615 GN=CEMIP PE=3 SV=1	0.35	0.21	133.4	56062704.2	8	1	0	0.24	1684	9775
>tr|A0A5F4D9S5|A0A5F4D9S5_CANLF Hyaluronoglucosaminidase OS=Canis lupus familiaris OX=9615 GN=CEMIP PE=3 SV=1	0.81	0.57	198.2	187265363	13	1	0	0.24	1684	9775
>tr|A0A5F4D9S5|A0A5F4D9S5_CANLF Hyaluronoglucosaminidase OS=Canis lupus familiaris OX=9615 GN=CEMIP PE=3 SV=1	0.55	0.55	187.3	4458486.7	1	1	0	0.24	1684	9775
>tr|J9PAQ2|J9PAQ2_CANLF Cyclin N-terminal domain-containing protein OS=Canis lupus familiaris OX=9615 PE=3 SV=1	0.1	0.01	58.2	195241904	11	1	0	1.25	400	2385
>tr|J9NTK2|J9NTK2_CANLF J domain-containing protein OS=Canis lupus familiaris OX=9615 GN=DNAJC12 PE=4 SV=2	0.27	0.03	202	68153747.8	13	1	0	4.72	106	2310

>tr|J9NTK2|J9NTK2_CANLF J domain-containing protein OS=Canis lupus familiaris OX=9615 GN=DNAJC12 PE=4 SV=2	0.1	0	164.1	100959245	6	1	0	4.72	106	2310
>tr|J9NTK2|J9NTK2_CANLF J domain-containing protein OS=Canis lupus familiaris OX=9615 GN=DNAJC12 PE=4 SV=2	0.1	0	104.3	33689209.6	2	1	0	4.72	106	2310
>tr|J9NTK2|J9NTK2_CANLF J domain-containing protein OS=Canis lupus familiaris OX=9615 GN=DNAJC12 PE=4 SV=2	0.23	0.21	160.3	13652893.2	2	1	0	4.72	106	2310
>tr|F1PJ71|F1PJ71_CANLF Glutathione peroxidase OS=Canis lupus familiaris OX=9615 GN=GPX5 PE=3 SV=2	0.63	0.57	215.9	4480646.2	4	1	1	6.33	221	19009
>tr|F1PJ71|F1PJ71_CANLF Glutathione peroxidase OS=Canis lupus familiaris OX=9615 GN=GPX5 PE=3 SV=2	2.53	2	304.6	106948022	16	4	0	25.34	221	19009
>tr|F1PJ71|F1PJ71_CANLF Glutathione peroxidase OS=Canis lupus familiaris OX=9615 GN=GPX5 PE=3 SV=2	1.47	0.58	271.1	122645604	14	4	1	15.84	221	19009
>sp|O46607|GPX5_CANLF Epididymal secretory glutathione peroxidase OS=Canis lupus familiaris OX=9615 GN=GPX5 PE=2 SV=1	1.01	0.85	233.1	15096457.5	4	2	0	8.6	221	564
>sp|O46607|GPX5_CANLF Epididymal secretory glutathione peroxidase OS=Canis lupus familiaris OX=9615 GN=GPX5 PE=2 SV=1	0.95	0.87	218.7	144258937	6	2	0	8.6	221	564
>sp|O46607|GPX5_CANLF Epididymal secretory glutathione peroxidase OS=Canis lupus familiaris OX=9615 GN=GPX5 PE=2 SV=1	3.59	1.94	157.9	1119106.1	8	3	0	11.76	221	564
>tr|E2R6E0|E2R6E0_CANLF Lipocln_cytosolic_FA-bd_dom domain-containing protein OS=Canis lupus familiaris OX=9615 GN=LCNL1 PE=3 SV=2	2.36	1.74	95.8	17961992.8	3	2	0	7.69	299	1932

>tr|E2R6E0|E2R6E0_CANLF Lipocln_cytosolic_FA-bd_dom domain-containing protein OS=Canis lupus familiaris OX=9615 GN=LCNL1 PE=3 SV=2	0.39	0.37	164.5	11428515.8	2	1	0	3.01	299	1932
>tr|E2R6E0|E2R6E0_CANLF Lipocln_cytosolic_FA-bd_dom domain-containing protein OS=Canis lupus familiaris OX=9615 GN=LCNL1 PE=3 SV=2	1.25	1.19	126.5	21536887.8	4	1	0	3.01	299	1932
>tr|E2R6E0|E2R6E0_CANLF Lipocln_cytosolic_FA-bd_dom domain-containing protein OS=Canis lupus familiaris OX=9615 GN=LCNL1 PE=3 SV=2	2.28	2.18	228.1	64505297.6	6	1	0	3.01	299	1932
>tr|E2R6E0|E2R6E0_CANLF Lipocln_cytosolic_FA-bd_dom domain-containing protein OS=Canis lupus familiaris OX=9615 GN=LCNL1 PE=3 SV=2	1.71	1.55	189	37651996.4	9	1	0	3.68	299	1932
>tr|E2R6E0|E2R6E0_CANLF Lipocln_cytosolic_FA-bd_dom domain-containing protein OS=Canis lupus familiaris OX=9615 GN=LCNL1 PE=3 SV=2	3.49	2.19	207.8	122698750	10	4	0	11.37	299	1932
>tr|E2R6E0|E2R6E0_CANLF Lipocln_cytosolic_FA-bd_dom domain-containing protein OS=Canis lupus familiaris OX=9615 GN=LCNL1 PE=3 SV=2	3.2	3.14	187.2	48134410.4	4	1	0	3.01	299	1932
>tr|E2R6E0|E2R6E0_CANLF Lipocln_cytosolic_FA-bd_dom domain-containing protein OS=Canis lupus familiaris OX=9615 GN=LCNL1 PE=3 SV=2	2.12	2.12	199.4	7947178.4	1	1	0	3.68	299	1932
>tr|F1PR54|F1PR54_CANLF Lactotransferrin OS=Canis lupus familiaris OX=9615 GN=LTF PE=3 SV=1	5.24	1.63	235.1	127971924	27	8	0	12.71	708	40436
>tr|A0A5F4BVF3|A0A5F4BVF3_CANLF Lactotransferrin OS=Canis lupus familiaris OX=9615 GN=LTF PE=3 SV=1	1.05	0.97	275.1	81961926.1	13	3	0	3.99	626	32850
>tr|F1PR54|F1PR54_CANLF Lactotransferrin OS=Canis lupus familiaris OX=9615 GN=LTF PE=3 SV=1	9.42	3.49	352	328762076	51	9	1	11.02	708	40436

>tr|A0A5F4BVF3|A0A5F4BVF3_CANLF Lactotransferrin OS=Canis lupus familiaris OX=9615 GN=LTF PE=3 SV=1	6.26	2.91	378.9	949659431	67	9	0	15.97	626	32850
>tr|A0A5F4BVF3|A0A5F4BVF3_CANLF Lactotransferrin OS=Canis lupus familiaris OX=9615 GN=LTF PE=3 SV=1	12.5	3.24	332	628759366	76	10	0	11.5	626	32850
>tr|A0A5F4BVF3|A0A5F4BVF3_CANLF Lactotransferrin OS=Canis lupus familiaris OX=9615 GN=LTF PE=3 SV=1	8.01	2.43	352.2	759899560	60	10	0	13.9	626	32850
>tr|F1PR54|F1PR54_CANLF Lactotransferrin OS=Canis lupus familiaris OX=9615 GN=LTF PE=3 SV=1	17.9	4.31	326.6	1652321844	108	12	3	15.68	708	40436
>tr|A0A5F4BVF3|A0A5F4BVF3_CANLF Lactotransferrin OS=Canis lupus familiaris OX=9615 GN=LTF PE=3 SV=1	8.24	2.47	289.5	708660873	51	8	0	7.99	626	32850
>tr|A0A5F4BVF3|A0A5F4BVF3_CANLF Lactotransferrin OS=Canis lupus familiaris OX=9615 GN=LTF PE=3 SV=1	4.84	2.92	299.4	87110930.6	17	3	0	3.99	626	32850
>tr|F1PR54|F1PR54_CANLF Lactotransferrin OS=Canis lupus familiaris OX=9615 GN=LTF PE=3 SV=1	18.69	3.07	397.9	294005519	75	15	0	18.22	708	40436
>tr|A0A5F4C0U6|A0A5F4C0U6_CANLF MLLT1 super elongation complex subunit OS=Canis lupus familiaris OX=9615 GN=MLLT1 PE=4 SV=1	0.1	0	138.5	313799141	34	2	0	1.17	597	1602
>sp|Q28895|NPC2_CANLF NPC intracellular cholesterol transporter 2 OS=Canis lupus familiaris OX=9615 GN=NPC2 PE=2 SV=1	8.08	3.25	414	187319399	27	4	0	30.2	149	153
>sp|Q28895|NPC2_CANLF NPC intracellular cholesterol transporter 2 OS=Canis lupus familiaris OX=9615 GN=NPC2 PE=2 SV=1	2.24	1.19	354.1	542889.1	5	2	0	14.77	149	153

>sp|Q28895|NPC2_CANLF NPC intracellular cholesterol transporter 2 OS=Canis lupus familiaris OX=9615 GN=NPC2 PE=2 SV=1	10.34	3.97	448.1	131596555	39	5	0	30.2	149	153
>sp|Q28895|NPC2_CANLF NPC intracellular cholesterol transporter 2 OS=Canis lupus familiaris OX=9615 GN=NPC2 PE=2 SV=1	11.28	5.59	422.9	182362787	25	4	0	30.2	149	153
>sp|Q28895|NPC2_CANLF NPC intracellular cholesterol transporter 2 OS=Canis lupus familiaris OX=9615 GN=NPC2 PE=2 SV=1	8.32	3.38	270.3	121461627	15	3	0	30.2	149	153
>sp|Q28895|NPC2_CANLF NPC intracellular cholesterol transporter 2 OS=Canis lupus familiaris OX=9615 GN=NPC2 PE=2 SV=1	11.15	4.65	591.2	305690404	52	3	0	30.2	149	153
>sp|Q28895|NPC2_CANLF NPC intracellular cholesterol transporter 2 OS=Canis lupus familiaris OX=9615 GN=NPC2 PE=2 SV=1	6.65	6.35	427	12973006	16	1	0	15.44	149	153
>sp|Q28895|NPC2_CANLF NPC intracellular cholesterol transporter 2 OS=Canis lupus familiaris OX=9615 GN=NPC2 PE=2 SV=1	1.35	1.35	162.3	152904.1	1	1	0	6.04	149	153
>tr|E2RE16|E2RE16_CANLF Non-specific serine/threonine protein kinase OS=Canis lupus familiaris OX=9615 GN=PAK4 PE=4 SV=1	0.34	0.08	95.3	52479900.2	41	1	0	0.84	592	12735
>tr|E2RE16|E2RE16_CANLF Non-specific serine/threonine protein kinase OS=Canis lupus familiaris OX=9615 GN=PAK4 PE=4 SV=1	0.1	0	57	14718612.4	34	1	0	0.84	592	12735
>tr|E2RE16|E2RE16_CANLF Non-specific serine/threonine protein kinase OS=Canis lupus familiaris OX=9615 GN=PAK4 PE=4 SV=1	0.65	0.02	80.4	112709410	43	1	0	0.84	592	12735
>tr|E2RE16|E2RE16_CANLF Non-specific serine/threonine protein kinase OS=Canis lupus familiaris OX=9615 GN=PAK4 PE=4 SV=1	0.61	0.02	86.7	112965242	57	1	0	0.84	592	12735

>tr|E2RE16|E2RE16_CANLF Non-specific serine/threonine protein kinase OS=Canis lupus familiaris OX=9615 GN=PAK4 PE=4 SV=1	0.18	0.02	57.6	59242568.1	23	1	0	0.84	592	12735
>sp|Q9XS65|PTGDS_CANLF Prostaglandin-H2 D-isomerase OS=Canis lupus familiaris OX=9615 GN=PTGDS PE=2 SV=1	0.24	0.18	119.4	31259064.7	5	2	1	10.47	191	165
>sp|Q9XS65|PTGDS_CANLF Prostaglandin-H2 D-isomerase OS=Canis lupus familiaris OX=9615 GN=PTGDS PE=2 SV=1	7.98	2.36	377.4	474370332	47	5	1	14.14	191	165
>sp|Q9XS65|PTGDS_CANLF Prostaglandin-H2 D-isomerase OS=Canis lupus familiaris OX=9615 GN=PTGDS PE=2 SV=1	2.19	1.42	297.6	149308819	12	2	0	10.47	191	165
>sp|Q9XS65|PTGDS_CANLF Prostaglandin-H2 D-isomerase OS=Canis lupus familiaris OX=9615 GN=PTGDS PE=2 SV=1	2.01	1.55	288.5	85454601.9	11	2	1	10.47	191	165
>sp|Q9XS65|PTGDS_CANLF Prostaglandin-H2 D-isomerase OS=Canis lupus familiaris OX=9615 GN=PTGDS PE=2 SV=1	2.71	1.83	322.1	123286339	11	2	1	10.47	191	165
>sp|Q9XS65|PTGDS_CANLF Prostaglandin-H2 D-isomerase OS=Canis lupus familiaris OX=9615 GN=PTGDS PE=2 SV=1	7.49	3.22	345.7	780863794	40	4	1	13.09	191	165
>sp|Q9XS65|PTGDS_CANLF Prostaglandin-H2 D-isomerase OS=Canis lupus familiaris OX=9615 GN=PTGDS PE=2 SV=1	0.49	0.41	222.2	1260896.8	5	1	1	7.33	191	165
>sp|Q9XS65|PTGDS_CANLF Prostaglandin-H2 D-isomerase OS=Canis lupus familiaris OX=9615 GN=PTGDS PE=2 SV=1	1.62	1.44	252.2	2203885.9	11	2	1	10.47	191	165
>sp|E2RKA8|RL32_CANLF 60S ribosomal protein L32 OS=Canis lupus familiaris OX=9615 GN=RPL32 PE=1 SV=1	1.24	0.02	111.4	460727872	89	2	0	4.44	135	275

>sp|E2RKA8|RL32_CANLF 60S ribosomal protein L32 OS=Canis lupus familiaris OX=9615 GN=RPL32 PE=1 SV=1	3.52	0.59	116.9	555722533	133	2	0	4.44	135	275
>tr|A0A5F4D6L9|A0A5F4D6L9_CANLF Sacsin molecular chaperone OS=Canis lupus familiaris OX=9615 GN=SACS PE=4 SV=1	1.02	0.85	229.7	92992927	17	4	1	0.33	4500	1444
>tr|A0A5F4D6L9|A0A5F4D6L9_CANLF Sacsin molecular chaperone OS=Canis lupus familiaris OX=9615 GN=SACS PE=4 SV=1	1.31	0.38	241.3	305031485	27	4	0	0.53	4500	1444
>tr|A0A5F4D6L9|A0A5F4D6L9_CANLF Sacsin molecular chaperone OS=Canis lupus familiaris OX=9615 GN=SACS PE=4 SV=1	0.84	0.78	236	37550451.5	5	2	0	0.18	4500	1444
>tr|A0A5F4D6L9|A0A5F4D6L9_CANLF Sacsin molecular chaperone OS=Canis lupus familiaris OX=9615 GN=SACS PE=4 SV=1	0.84	0.82	206.6	9407695.2	2	1	0	0.09	4500	1444
>tr|A0A5F4D6L9|A0A5F4D6L9_CANLF Sacsin molecular chaperone OS=Canis lupus familiaris OX=9615 GN=SACS PE=4 SV=1	0.81	0.54	194.6	140131502	44	3	0	0.27	4500	1444
>tr|E2QRT5|E2QRT5_CANLF Structural maintenance of chromosomes protein OS=Canis lupus familiaris OX=9615 GN=SMC1B PE=3 SV=1	0.86	0.44	64.4	496319667	22	1	0	0.49	1235	2380
>tr|F1PBU5|F1PBU5_CANLF Non-specific serine/threonine protein kinase OS=Canis lupus familiaris OX=9615 GN=SMG1 PE=3 SV=3	0.63	0.63	114.8	5257953.9	1	1	0	0.08	3634	6898
>tr|F1PBU5|F1PBU5_CANLF Non-specific serine/threonine protein kinase OS=Canis lupus familiaris OX=9615 GN=SMG1 PE=3 SV=3	0.36	0.31	167.7	33714348.1	4	1	0	0.08	3634	6898
>tr|F1PBU5|F1PBU5_CANLF Non-specific serine/threonine protein kinase OS=Canis lupus familiaris OX=9615 GN=SMG1 PE=3 SV=3	1.51	0.81	177.2	40793140.9	10	2	0	0.17	3634	6898

>tr|F1PBU5|F1PBU5_CANLF Non-specific serine/threonine protein kinase OS=Canis lupus familiaris OX=9615 GN=SMG1 PE=3 SV=3	0.83	0.83	173.3	8268862.3	1	1	0	0.08	3634	6898
>tr|F1PBU5|F1PBU5_CANLF Non-specific serine/threonine protein kinase OS=Canis lupus familiaris OX=9615 GN=SMG1 PE=3 SV=3	0.68	0.33	172	229564679	17	2	0	0.17	3634	6898
>tr|A0A5K1V0D8|A0A5K1V0D8_CANLF Sulfatase 2 OS=Canis lupus familiaris OX=9615 GN=SULF2 PE=3 SV=1	0.1	0	180	21470834.1	4	1	0	0.35	859	1192
>tr|A0A5K1V0D8|A0A5K1V0D8_CANLF Sulfatase 2 OS=Canis lupus familiaris OX=9615 GN=SULF2 PE=3 SV=1	0.42	0.17	181.6	157954122	15	2	0	0.81	859	1192
>tr|A0A5K1V0D8|A0A5K1V0D8_CANLF Sulfatase 2 OS=Canis lupus familiaris OX=9615 GN=SULF2 PE=3 SV=1	0.67	0.61	201.3	26246759.4	4	1	0	0.35	859	1192
>tr|A0A5K1V0D8|A0A5K1V0D8_CANLF Sulfatase 2 OS=Canis lupus familiaris OX=9615 GN=SULF2 PE=3 SV=1	0.1	0.02	189.6	64093192.6	11	1	0	0.35	859	1192
>tr|A0A5K1V0D8|A0A5K1V0D8_CANLF Sulfatase 2 OS=Canis lupus familiaris OX=9615 GN=SULF2 PE=3 SV=1	0.66	0.56	184.5	22817089.7	6	1	0	0.35	859	1192
>tr|A0A5F4CUD4|A0A5F4CUD4_CANLF Transcription initiation factor TFIID subunit OS=Canis lupus familiaris OX=9615 GN=TAF1 PE=3 SV=1	0.61	0.12	101.2	379105437	33	2	0	0.53	1897	4265
>tr|A0A5F4BU36|A0A5F4BU36_CANLF Titin OS=Canis lupus familiaris OX=9615 GN=TTN PE=3 SV=1	0.52	0.54	42.2	273602.7	1	1	0	0.08	27097	33785
>tr|A0A5F4BU36|A0A5F4BU36_CANLF Titin OS=Canis lupus familiaris OX=9615 GN=TTN PE=3 SV=1	0.23	0.03	38.3	175131771	15	2	0	0.04	27097	33785
>tr|E2RCT1|E2RCT1_CANLF WAP domain-containing protein OS=Canis lupus familiaris OX=9615 PE=4 SV=2	3.82	2.59	249	28068068.5	5	2	0	14.66	116	21717

>tr|E2RCT1|E2RCT1_CANLF WAP domain-containing protein OS=Canis lupus familiaris OX=9615 PE=4 SV=2	1.33	1.31	221.5	548065.3	2	1	0	14.66	116	21717
>tr|E2RCT1|E2RCT1_CANLF WAP domain-containing protein OS=Canis lupus familiaris OX=9615 PE=4 SV=2	6.73	3.2	315.3	602951559	30	2	0	9.48	116	21717
>tr|E2RCT1|E2RCT1_CANLF WAP domain-containing protein OS=Canis lupus familiaris OX=9615 PE=4 SV=2	1.47	1.4	135.9	40419871.7	3	2	0	9.48	116	21717
>tr|E2RCT1|E2RCT1_CANLF WAP domain-containing protein OS=Canis lupus familiaris OX=9615 PE=4 SV=2	1.97	1.75	278.4	109486759	7	2	0	9.48	116	21717
>tr|A0A5F4BQW4|A0A5F4BQW4_CANLF Zinc finger protein 654 OS=Canis lupus familiaris OX=9615 GN=ZNF654 PE=4 SV=1	0.11	0.1	203.5	23135482.6	4	1	1	1.03	1170	6910
>tr|A0A5F4BQW4|A0A5F4BQW4_CANLF Zinc finger protein 654 OS=Canis lupus familiaris OX=9615 GN=ZNF654 PE=4 SV=1	0.65	0.35	184.7	215177541	16	1	1	1.03	1170	6910
