# Supplementary material for: Potential Proteins Associated with Canine Epididymal Sperm Motility
Source: Cells. 2026 Jan 4;15(1):85. doi: 10.3390/cells15010085 (PMC12786130; doi:10.3390/cells15010085)
Supplement: Supplementary file 1 [file cells-15-00085-s001.zip › Supplementary Table S4.pdf]

Supplementary Table S4. The set of canine epididymal sperm (ES) proteins that constitute <1% in the poor sperm motility (PSM) group.

Description	|Log Prob|	Best |Log Prob|	Best score	Total Intensity	# of spectra	# of unique peptides	# of mod peptides	Coverage %	# AA's in protein	Protein DB number
>sp|O18840|ACTB_CANLF Actin, cytoplasmic 1 OS=Canis lupus familiaris OX=9615 GN=ACTB PE=2 SV=3	6.01	3.93	316.4	215049910	16	3	0	10.13	375	642
>sp|O18840|ACTB_CANLF Actin, cytoplasmic 1 OS=Canis lupus familiaris OX=9615 GN=ACTB PE=2 SV=3	0.1	0.06	190.6	26426006.5	3	1	0	2.93	375	642
>sp|O18840|ACTB_CANLF Actin, cytoplasmic 1 OS=Canis lupus familiaris OX=9615 GN=ACTB PE=2 SV=3	1.78	1.56	240.9	226071179	12	2	0	7.73	375	642
>sp|O18840|ACTB_CANLF Actin, cytoplasmic 1 OS=Canis lupus familiaris OX=9615 GN=ACTB PE=2 SV=3	7.57	4.92	325.7	148633566	22	2	0	5.33	375	642
>sp|O18840|ACTB_CANLF Actin, cytoplasmic 1 OS=Canis lupus familiaris OX=9615 GN=ACTB PE=2 SV=3	4.97	4.18	383.1	113426229	19	2	0	5.33	375	642
>sp|O18840|ACTB_CANLF Actin, cytoplasmic 1 OS=Canis lupus familiaris OX=9615 GN=ACTB PE=2 SV=3	4.27	3.7	334.8	137279041	13	2	0	4.53	375	642
>sp|O18840|ACTB_CANLF Actin, cytoplasmic 1 OS=Canis lupus familiaris OX=9615 GN=ACTB PE=2 SV=3	0.3	0.24	150	6127412.6	4	1	0	2.93	375	642
>sp|P49822|ALBU_CANLF Albumin OS=Canis lupus familiaris OX=9615 GN=ALB PE=1 SV=3	10.77	4.02	295.2	624251171	36	7	0	14.8	608	490
>sp|P49822|ALBU_CANLF Albumin OS=Canis lupus familiaris OX=9615 GN=ALB PE=1 SV=3	0.3	0.25	178.5	33756533.6	7	2	1	2.3	608	490

>sp|P49822|ALBU_CANLF Albumin OS=Canis lupus familiaris OX=9615 GN=ALB PE=1 SV=3	9.81	3.92	287.8	12969291.6	13	6	1	11.51	608	490
>sp|P49822|ALBU_CANLF Albumin OS=Canis lupus familiaris OX=9615 GN=ALB PE=1 SV=3	3.86	2.52	211.1	188982271	22	4	0	8.39	608	490
>tr|A0A5F4CAK8|A0A5F4CAK8_CANLF Ankyrin repeat and sterile alpha motif domain containing 1B OS=Canis lupus familiaris OX=9615 GN=ANKS1B PE=4 SV=1	0.1	0	49.5	114257500	13	2	2	0.86	1281	31927
>tr|A0A5F4DHJ7|A0A5F4DHJ7_CANLF APC-binding protein EB1 OS=Canis lupus familiaris OX=9615 PE=3 SV=1	0.1	0	33.6	156720911	19	2	2	1.67	300	38718
>tr|F1P920|F1P920_CANLF Apoptosis resistant E3 ubiquitin protein ligase 1 OS=Canis lupus familiaris OX=9615 GN=AREL1 PE=4 SV=3	0.1	0	71.9	1167711957	15	1	0	0.88	793	14252
>tr|F1PGF9|F1PGF9_CANLF Rho guanine nucleotide exchange factor 26 OS=Canis lupus familiaris OX=9615 GN=ARHGEF26 PE=4 SV=3	0.57	0.53	266.9	76427460.5	3	1	0	0.84	594	22876
>tr|F1PGF9|F1PGF9_CANLF Rho guanine nucleotide exchange factor 26 OS=Canis lupus familiaris OX=9615 GN=ARHGEF26 PE=4 SV=3	1.24	1.2	296.7	25220664	3	1	0	0.84	594	22876
>tr|F1PGF9|F1PGF9_CANLF Rho guanine nucleotide exchange factor 26 OS=Canis lupus familiaris OX=9615 GN=ARHGEF26 PE=4 SV=3	0.74	0.7	304.4	34821887.7	3	1	0	0.84	594	22876
>sp|P62286|ASPM_CANLF Abnormal spindle-like microcephaly-associated protein homolog OS=Canis lupus familiaris OX=9615 GN=ASPM PE=2 SV=2	0.6	0.49	150.8	394855698	87	3	0	0.23	3469	677
>sp|P62286|ASPM_CANLF Abnormal spindle-like microcephaly-associated protein homolog OS=Canis lupus familiaris OX=9615 GN=ASPM PE=2 SV=2	0.29	0.1	43.3	38400604.6	10	2	0	0.23	3469	677

>sp|P62286|ASPM_CANLF Abnormal spindle-like microcephaly-associated protein homolog OS=Canis lupus familiaris OX=9615 GN=ASPM PE=2 SV=2	0.33	0.13	114.8	45431255.6	17	2	0	0.23	3469	677
>tr|A0A5F4CR89|A0A5F4CR89_CANLF Voltage-dependent R-type calcium channel subunit alpha OS=Canis lupus familiaris OX=9615 GN=CACNA1E PE=3 SV=1	0.61	0.38	54.1	255892986	13	1	0	0.19	2688	1145
>tr|A0A5F4C1S8|A0A5F4C1S8_CANLF E3 ubiquitin-protein ligase CBL OS=Canis lupus familiaris OX=9615 GN=CBL PE=4 SV=1	0.19	0.17	144.9	6581470.8	2	1	0	0.39	773	1308
>tr|A0A5F4C1S8|A0A5F4C1S8_CANLF E3 ubiquitin-protein ligase CBL OS=Canis lupus familiaris OX=9615 GN=CBL PE=4 SV=1	0.5	0.08	153.6	151951513	21	2	0	0.91	773	1308
>tr|Q9XSV4|Q9XSV4_CANLF CE10 protein OS=Canis lupus familiaris OX=9615 GN=ce10 PE=2 SV=1	3.99	2.29	391.7	122646525	23	2	0	9.09	110	41542
>tr|Q9XSV4|Q9XSV4_CANLF CE10 protein OS=Canis lupus familiaris OX=9615 GN=ce10 PE=2 SV=1	1.65	0.82	333.9	215728728	23	3	0	14.55	110	41542
>tr|Q9XSV4|Q9XSV4_CANLF CE10 protein OS=Canis lupus familiaris OX=9615 GN=ce10 PE=2 SV=1	5.15	3.99	327.8	408366089	35	3	0	12.73	110	41542
>tr|Q9XSV4|Q9XSV4_CANLF CE10 protein OS=Canis lupus familiaris OX=9615 GN=ce10 PE=2 SV=1	1.38	1.12	233.1	19989969.8	6	3	0	14.55	110	41542
>tr|Q9XSV4|Q9XSV4_CANLF CE10 protein OS=Canis lupus familiaris OX=9615 GN=ce10 PE=2 SV=1	0.58	0.42	225	14071262.5	7	2	0	9.09	110	41542
>tr|Q9XSV4|Q9XSV4_CANLF CE10 protein OS=Canis lupus familiaris OX=9615 GN=ce10 PE=2 SV=1	2.59	1.77	249.1	24116860.5	12	3	0	14.55	110	41542
>tr|Q9XSV4|Q9XSV4_CANLF CE10 protein OS=Canis lupus familiaris OX=9615 GN=ce10 PE=2 SV=1	1.24	0.87	210.7	14879128.5	7	2	0	11.82	110	41542
>sp|P25473|CLUS_CANLF Clusterin OS=Canis lupus familiaris OX=9615 GN=CLU PE=2 SV=1	0.38	0.34	218.4	53142113.9	3	1	0	0.9	445	725

>sp|P25473|CLUS_CANLF Clusterin OS=Canis lupus familiaris OX=9615 GN=CLU PE=2 SV=1	5.63	2.22	328.3	211355224	32	7	0	8.99	445	725
>tr|J9PAQ2|J9PAQ2_CANLF Cyclin N-terminal domain-containing protein OS=Canis lupus familiaris OX=9615 PE=3 SV=1	0.62	0.38	54.1	255892986	13	1	0	1.25	400	2385
>tr|A0A5F4CCD0|A0A5F4CCD0_CANLF Cysteine rich secretory protein 2 OS=Canis lupus familiaris OX=9615 GN=CRISP2 PE=3 SV=1	1.54	1.48	112.2	21777312	4	1	0	4.82	311	11017
>tr|A0A5F4CCD0|A0A5F4CCD0_CANLF Cysteine rich secretory protein 2 OS=Canis lupus familiaris OX=9615 GN=CRISP2 PE=3 SV=1	0.89	0.84	177.5	351994.1	4	1	0	4.82	311	11017
>tr|A0A5F4CCD0|A0A5F4CCD0_CANLF Cysteine rich secretory protein 2 OS=Canis lupus familiaris OX=9615 GN=CRISP2 PE=3 SV=1	1.32	1.26	213.5	75936743.2	5	2	0	7.07	311	11017
>tr|A0A5F4CCD0|A0A5F4CCD0_CANLF Cysteine rich secretory protein 2 OS=Canis lupus familiaris OX=9615 GN=CRISP2 PE=3 SV=1	0.3	0.3	170.4	4780781.6	1	1	0	2.25	311	11017
>tr|A0A5F4CCD0|A0A5F4CCD0_CANLF Cysteine rich secretory protein 2 OS=Canis lupus familiaris OX=9615 GN=CRISP2 PE=3 SV=1	1.67	1.67	175	703679.8	1	1	0	4.82	311	11017
>tr|A0A5F4CCD0|A0A5F4CCD0_CANLF Cysteine rich secretory protein 2 OS=Canis lupus familiaris OX=9615 GN=CRISP2 PE=3 SV=1	0.67	0.63	190	15676538.4	3	1	0	2.25	311	11017
>tr|F1PJY1|F1PJY1_CANLF Mannosyl-glycoprotein endo-beta-N-acetylglucosaminidase OS=Canis lupus familiaris OX=9615 GN=ENGASE PE=3 SV=3	0.1	0.04	137.4	3758465.7	2	1	1	1.74	690	32761
>tr|F1PJY1|F1PJY1_CANLF Mannosyl-glycoprotein endo-beta-N-acetylglucosaminidase OS=Canis lupus familiaris OX=9615 GN=ENGASE PE=3 SV=3	1.06	0.39	154	146672909	17	3	2	1.74	690	32761

>tr|F1PJY1|F1PJY1_CANLF Mannosyl-glycoprotein endo-beta-N-acetylglucosaminidase OS=Canis lupus familiaris OX=9615 GN=ENGASE PE=3 SV=3	0.7	0.25	176.2	15431288.8	16	2	1	1.74	690	32761
>tr|F1PJH2|F1PJH2_CANLF FAT atypical cadherin 3 OS=Canis lupus familiaris OX=9615 GN=FAT3 PE=4 SV=3	0.1	0	50.4	214399990	30	2	2	0.11	4557	3984
>sp|O46607|GPX5_CANLF Epididymal secretory glutathione peroxidase OS=Canis lupus familiaris OX=9615 GN=GPX5 PE=2 SV=1	0.73	0.71	76	19747744.3	2	1	0	6.79	221	564
>tr|F1PJ71|F1PJ71_CANLF Glutathione peroxidase OS=Canis lupus familiaris OX=9615 GN=GPX5 PE=3 SV=2	6.5	2.91	302.2	318253471	21	6	1	29.86	221	19009
>tr|F1PJ71|F1PJ71_CANLF Glutathione peroxidase OS=Canis lupus familiaris OX=9615 GN=GPX5 PE=3 SV=2	11.93	3.79	293.4	558589605	68	8	1	30.32	221	19009
>tr|F6Y6X6|F6Y6X6_CANLF IQ motif containing GTPase activating protein 3 OS=Canis lupus familiaris OX=9615 GN=IQGAP3 PE=4 SV=2	0.18	0.06	91.1	326817609	19	3	1	0.95	1693	7673
>tr|J9NS29|J9NS29_CANLF Cystatin domain-containing protein OS=Canis lupus familiaris OX=9615 GN=LOC607874 PE=4 SV=2	4.61	2.46	289.6	114468559	10	3	0	16.29	313	30016
>tr|J9NS29|J9NS29_CANLF Cystatin domain-containing protein OS=Canis lupus familiaris OX=9615 GN=LOC607874 PE=4 SV=2	0.21	0.21	111	5867607.9	1	1	0	6.39	313	30016
>tr|J9NS29|J9NS29_CANLF Cystatin domain-containing protein OS=Canis lupus familiaris OX=9615 GN=LOC607874 PE=4 SV=2	0.32	0.28	111.9	30625104.5	3	1	0	4.79	313	30016
>tr|F1PR54|F1PR54_CANLF Lactotransferrin OS=Canis lupus familiaris OX=9615 GN=LTF PE=3 SV=1	21.93	4.03	351.3	622958393	69	11	0	14.41	708	40436

>tr|A0A5F4BVF3|A0A5F4BVF3_CANLF Lactotransferrin OS=Canis lupus familiaris OX=9615 GN=LTF PE=3 SV=1	3.85	1.27	312.3	273240797	28	5	0	8.15	626	32850
>tr|F1PR54|F1PR54_CANLF Lactotransferrin OS=Canis lupus familiaris OX=9615 GN=LTF PE=3 SV=1	19.74	4.25	327.4	1267254089	84	13	1	18.22	708	40436
>tr|A0A5F4BVF3|A0A5F4BVF3_CANLF Lactotransferrin OS=Canis lupus familiaris OX=9615 GN=LTF PE=3 SV=1	8.76	3.38	331.9	192363156	39	6	0	9.42	626	32850
>tr|A0A5F4BVF3|A0A5F4BVF3_CANLF Lactotransferrin OS=Canis lupus familiaris OX=9615 GN=LTF PE=3 SV=1	2.97	0.77	245.2	85106957.9	21	8	0	12.78	626	32850
>tr|F1PR54|F1PR54_CANLF Lactotransferrin OS=Canis lupus familiaris OX=9615 GN=LTF PE=3 SV=1	18.92	3.64	313.9	109770182	47	12	0	15.96	708	40436
>tr|F1PR54|F1PR54_CANLF Lactotransferrin OS=Canis lupus familiaris OX=9615 GN=LTF PE=3 SV=1	23.56	4.15	274.1	575486227	90	14	1	22.03	708	40436
>sp|Q28895|NPC2_CANLF NPC intracellular cholesterol transporter 2 OS=Canis lupus familiaris OX=9615 GN=NPC2 PE=2 SV=1	10.26	4.49	381.7	191672362	21	4	0	30.2	149	153
>sp|Q28895|NPC2_CANLF NPC intracellular cholesterol transporter 2 OS=Canis lupus familiaris OX=9615 GN=NPC2 PE=2 SV=1	0.1	0.02	137.5	13609297.8	2	2	0	21.48	149	153
>sp|Q28895|NPC2_CANLF NPC intracellular cholesterol transporter 2 OS=Canis lupus familiaris OX=9615 GN=NPC2 PE=2 SV=1	18.28	5.26	587.3	788208902	68	4	0	36.91	149	153
>sp|Q28895|NPC2_CANLF NPC intracellular cholesterol transporter 2 OS=Canis lupus familiaris OX=9615 GN=NPC2 PE=2 SV=1	0.79	0.68	102.4	460040.4	2	2	0	14.77	149	153

>sp|Q28895|NPC2_CANLF NPC intracellular cholesterol transporter 2 OS=Canis lupus familiaris OX=9615 GN=NPC2 PE=2 SV=1	0.7	0.64	172.6	19345909.6	4	1	0	15.44	149	153
>sp|Q28895|NPC2_CANLF NPC intracellular cholesterol transporter 2 OS=Canis lupus familiaris OX=9615 GN=NPC2 PE=2 SV=1	6.55	2.18	337.1	22906105.3	14	4	0	38.93	149	153
>sp|Q28895|NPC2_CANLF NPC intracellular cholesterol transporter 2 OS=Canis lupus familiaris OX=9615 GN=NPC2 PE=2 SV=1	3.96	1.84	408.1	56541705.1	29	4	0	36.91	149	153
>tr|F1PPZ3|F1PPZ3_CANLF Olfactory receptor OS=Canis lupus familiaris OX=9615 GN=OR6S1 PE=3 SV=2	0.1	0.01	47.2	131347352	13	1	1	1.21	331	30247
>tr|A0A5F4DD58|A0A5F4DD58_CANLF Phosphoinositide phospholipase C OS=Canis lupus familiaris OX=9615 GN=PLCD3 PE=4 SV=1	0.9	0.8	205.9	33023756.5	6	1	0	0.4	741	3088
>tr|A0A5F4DD58|A0A5F4DD58_CANLF Phosphoinositide phospholipase C OS=Canis lupus familiaris OX=9615 GN=PLCD3 PE=4 SV=1	0.24	0.02	146.6	656412059	83	4	1	1.48	741	3088
>tr|A0A5F4DD58|A0A5F4DD58_CANLF Phosphoinositide phospholipase C OS=Canis lupus familiaris OX=9615 GN=PLCD3 PE=4 SV=1	0.51	0.31	230.4	232039411	140	3	0	1.48	741	3088
>tr|A0A5F4CEM0|A0A5F4CEM0_CANLF Proline rich coiled-coil 2B OS=Canis lupus familiaris OX=9615 GN=PRRC2B PE=4 SV=1	0.16	0.02	127.9	135370940	13	1	1	0.58	2235	37089
>sp|Q9XS65|PTGDS_CANLF Prostaglandin-H2 D-isomerase OS=Canis lupus familiaris OX=9615 GN=PTGDS PE=2 SV=1	3.45	1.81	308.7	368463965	17	2	1	10.47	191	165
>sp|Q9XS65|PTGDS_CANLF Prostaglandin-H2 D-isomerase OS=Canis lupus familiaris OX=9615 GN=PTGDS PE=2 SV=1	4.2	2.78	330.7	123756321	18	3	1	13.61	191	165

>sp|Q9XS65|PTGDS_CANLF Prostaglandin-H2 D-isomerase OS=Canis lupus familiaris OX=9615 GN=PTGDS PE=2 SV=1	1.04	0.72	234.2	3595633.3	7	2	1	10.47	191	165
>sp|Q9XS65|PTGDS_CANLF Prostaglandin-H2 D-isomerase OS=Canis lupus familiaris OX=9615 GN=PTGDS PE=2 SV=1	5.28	2.2	294.9	434245415	55	6	1	24.08	191	165
>tr|E2RRF5|E2RRF5_CANLF RNA binding motif protein 19 OS=Canis lupus familiaris OX=9615 GN=RBM19 PE=4 SV=3	0.1	0.01	101.4	131832453	44	2	0	0.72	970	905
>tr|E2RRF5|E2RRF5_CANLF RNA binding motif protein 19 OS=Canis lupus familiaris OX=9615 GN=RBM19 PE=4 SV=3	0.13	0.11	43.3	3068568.4	2	1	0	0.41	970	905
>sp|E2RKA8|RL32_CANLF 60S ribosomal protein L32 OS=Canis lupus familiaris OX=9615 GN=RPL32 PE=1 SV=1	1.38	0.04	94	358661370	68	2	0	4.44	135	275
>sp|E2RKA8|RL32_CANLF 60S ribosomal protein L32 OS=Canis lupus familiaris OX=9615 GN=RPL32 PE=1 SV=1	1.9	0.15	167.3	351563559	87	2	0	4.44	135	275
>sp|E2RKA8|RL32_CANLF 60S ribosomal protein L32 OS=Canis lupus familiaris OX=9615 GN=RPL32 PE=1 SV=1	0.29	0.03	110.2	41596467.8	17	1	0	2.22	135	275
>tr|A0A5F4D6L9|A0A5F4D6L9_CANLF Sacsin molecular chaperone OS=Canis lupus familiaris OX=9615 GN=SACS PE=4 SV=1	0.1	0.01	75.3	331955035	43	1	0	0.09	4500	1444
>tr|A0A5F4D6L9|A0A5F4D6L9_CANLF Sacsin molecular chaperone OS=Canis lupus familiaris OX=9615 GN=SACS PE=4 SV=1	0.2	0.14	226.8	30959033.6	9	4	0	0.58	4500	1444
>tr|F1PBU5|F1PBU5_CANLF Non-specific serine/threonine protein kinase OS=Canis lupus familiaris OX=9615 GN=SMG1 PE=3 SV=3	0.95	0.39	163.6	167474779	11	2	0	0.17	3634	6898

>tr|F1PBU5|F1PBU5_CANLF Non-specific serine/threonine protein kinase OS=Canis lupus familiaris OX=9615 GN=SMG1 PE=3 SV=3	0.8	0.49	201.1	58165901.4	13	2	0	0.17	3634	6898
>tr|F1PGF6|F1PGF6_CANLF Sprouty related EVH1 domain containing 1 OS=Canis lupus familiaris OX=9615 GN=SPRED1 PE=4 SV=2	0.24	0.02	107.1	258992145	20	1	0	0.9	443	10620
>tr|F1P6X5|F1P6X5_CANLF ST6 N-acetylgalactosaminide alpha-2,6-sialyltransferase 5 OS=Canis lupus familiaris OX=9615 GN=ST6GALNAC5 PE=3 SV=3	0.22	0.02	84.5	246113204	19	1	0	1.18	338	4902
>tr|J9P0B4|J9P0B4_CANLF Tudor domain containing 15 OS=Canis lupus familiaris OX=9615 GN=TDRD15 PE=4 SV=2	1.59	1.43	231.6	186749629	9	1	0	0.57	2105	4188
>tr|J9P0B4|J9P0B4_CANLF Tudor domain containing 15 OS=Canis lupus familiaris OX=9615 GN=TDRD15 PE=4 SV=2	0.67	0.65	153.1	1026905.3	2	1	0	0.57	2105	4188
>tr|F1PBJ1|F1PBJ1_CANLF Methylcytosine dioxygenase TET OS=Canis lupus familiaris OX=9615 GN=TET3 PE=3 SV=2	0.1	0.02	32.5	25839500.3	2	1	0	0.28	1795	1529
>tr|F1PBJ1|F1PBJ1_CANLF Methylcytosine dioxygenase TET OS=Canis lupus familiaris OX=9615 GN=TET3 PE=3 SV=2	0.1	0	86.8	53409123.6	5	1	0	0.28	1795	1529
>tr|F1PBJ1|F1PBJ1_CANLF Methylcytosine dioxygenase TET OS=Canis lupus familiaris OX=9615 GN=TET3 PE=3 SV=2	0.4	0.1	44.8	62836488.3	16	1	0	0.28	1795	1529
>sp|Q697L1|TRPV1_CANLF Transient receptor potential cation channel subfamily V member 1 OS=Canis lupus familiaris OX=9615 GN=TRPV1 PE=2 SV=1	2.18	0.16	115.7	587367199	118	3	2	0.71	840	297

>tr|E2RCT1|E2RCT1_CANLF WAP domain-containing protein OS=Canis lupus familiaris OX=9615 PE=4 SV=2	0.43	0.4	186.1	17984233.1	3	1	0	14.66	116	21717
>tr|E2RCT1|E2RCT1_CANLF WAP domain-containing protein OS=Canis lupus familiaris OX=9615 PE=4 SV=2	3.06	1.64	242.5	74730589.2	12	2	0	9.48	116	21717
>tr|E2RCT1|E2RCT1_CANLF WAP domain-containing protein OS=Canis lupus familiaris OX=9615 PE=4 SV=2	0.62	0.28	173.6	27296972.7	6	3	0	24.14	116	21717
